# Evaluate the safety of a novel photohydrolysis technology used to clean and disinfect indoor air: A murine study

**DOI:** 10.1371/journal.pone.0307031

**Published:** 2024-10-09

**Authors:** Claude P. Selitrennikoff, Charles Sylvia, Maria Sanchez, Patricia Lawrence, Kimberly Trosch, Amy Carenza, Carol Meschter

**Affiliations:** 1 Department of Cell and Developmental Biology, University of Colorado School of Medicine, Aurora, Colorado, United States of America; 2 Comparative Biosciences, Inc., Sunnyvale, California, United States of America; 3 Consultant, Dunedin, Florida, United States of America; 4 ActivePure Technologies, Dallas, Texas, United States of America; SKUMS: Shahrekord University of Medical Science, ISLAMIC REPUBLIC OF IRAN

## Abstract

There is a pressing need to develop new technologies that continuously eliminates harmful pollutants and pathogens in occupied indoor spaces without compromising safety. This study was undertaken to test the safety of a novel air cleaning and disinfection technology called Advanced Photohydrolysis. Advanced Photohydrolysis generates a complex mixture of ions and molecules that are released into the air and has been shown to reduce airborne and surface pathogens. Mice (6–8-week-old) were exposed to therapeutic levels of Advanced Photohydrolysis for 90-days. During the study, the Advanced-Photohydrolysis-exposed and control mice were monitored for food consumption, body weight gain, and any overt adverse effects. In addition, at the conclusion of the study, the blood chemistry and hematology values of both groups were determined. Finally, the tissues of the conduction and respiratory portions of the airways of mice from both groups were examined for any pathological changes. The mice of both groups were found to be normal and healthy throughout the 90-day study; there were no differences in the behavior, food consumption and weight gain. Analysis of clinical chemistry values found no differences in hepatocellular function or other markers of cellular and organ function, and clinical hematology values were also unremarkable. Finally, and importantly, histopathology of the upper and lower airway tissues showed no deleterious effects. These results are the first to demonstrate directly the safety of Advanced Photohydrolysis on live mammals and encourage additional studies.

## Introduction

The spread of respiratory pathogens, particularly those that are public health threats (*e*.*g*., the severe acute respiratory syndrome [SARS] outbreak of 2003 and the SARS-Cov-2 pandemic of 2019), has underscored the ever-increasing need to improve indoor air quality and the management of infectious agents [1]. Additionally, other indoor air pollution/contamination has been long recognized to cause a variety of ailments, including allergies, shortness of breath, and Sick Building Syndrome symptoms [2, 3].

Indoor air is complex and extremely dynamic, with abundant sources of contamination [4]. In addition to volatile organic compounds (VOCs) dispersed from cleaning products, construction materials, adhesives, and copy machines, [5], there are constant biological exposures as well. These include microbes that naturally slough-off with skin cells [6–8], respiratory droplets released during speech, coughing, and laughing, [9, 10], plumes of aerosols from flushing toilets, [11, 12] and the omnipresence of molds and fungi [13–15].

To reduce the microbial burden and VOCs in the indoor environment, ventilation systems move air toward an exhaust point [14, 16]. To complicate matters, the time that a pollutant remains suspended in the air depends on its size/weight and desiccation properties [17]. To compensate for these limitations, the new ASHRAE 241 Standard suggests a 3- to 4-fold increase in cubic feet per minute of ventilation per person to reduce transmission risks, requiring significant cost increases to achieve healthier buildings [18].

Current technologies that serve as an adjunct to heating, ventilation, and air conditioning systems include ultraviolet light (UV), ozone, microbicides (*e*.*g*., hydrogen peroxide), and electrostatic precipitation. Unfortunately, each of these comes with their own safety hazards and limitations. The effects of UV technologies are intermittent because once the UV light is removed, the environment becomes re-contaminated soon thereafter [19]. Ozone is another method used to reduce infectious aerosols, but the level of ozone needed to destroy pathogens is hazardous to human health [20]. Similar to UV light, hydrogen peroxide and electrostatic precipitation are episodic and are not intended for use in occupied spaces when transmission risk is the greatest [19]. Photocatalytic oxidation has promising results, but little research has been published on its ability to reduce indoor air pollution [2, 21, 22].

**Fig 1:**
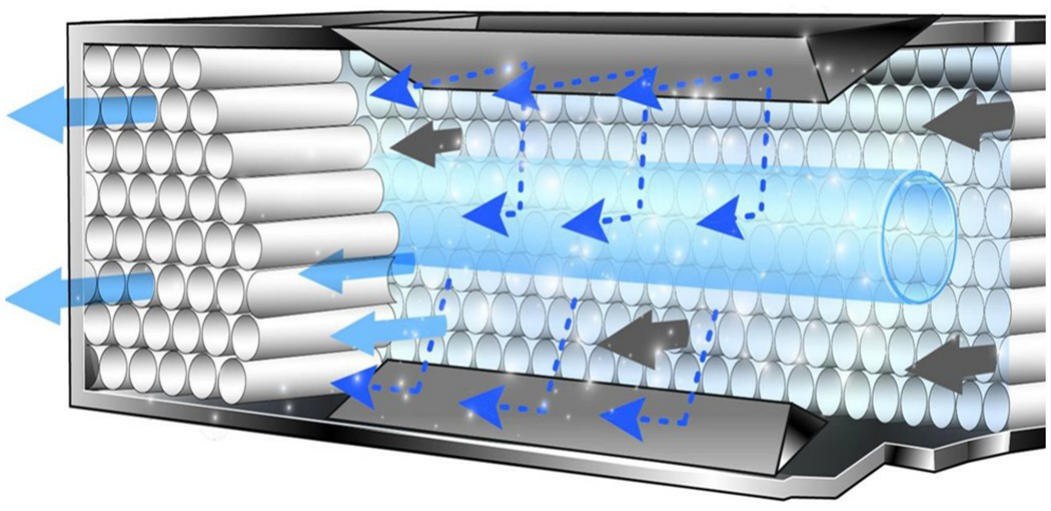
Cross-sectional view of an Advanced Photohydrolysis reactor. As air (grey arrows) flows through the honeycomb-shaped photocatalyst, the energy of the UV light (light blue cylinder) induces a series of photochemical reactions (blue arrows) to produce a complex mixture of molecules and ions (light blue arrows) that are released into the air flow.

Clearly, a technology that can continually eliminate pathogens and clear the air of other pollutants, without harming living mammals, is needed. One technology (*i*.*e*., Advanced Photohydrolysis [AP]) is on the cutting edge of achieving this goal. AP is an innovative and unique process for reducing indoor air pollution. AP has number of advantages over currently-used methods—these include ease of operation, low energy consumption, high efficiency, and no secondary pollution. The AP device consists of a patented cell containing a 253.8 ηm UV bulb surrounded by a proprietary, honeycomb-shaped matrix made from a hydrophilic polycarbonate and coated with a blend of metallic semiconductors and rare earth minerals, including TiO_2_, to function as a UV reactor (this is shown diagrammatically in Fig 1: Please see S1–S3 Files for relevant patents).

As air passes through the AP cell, the photocatalyst augments the energy of the UV light to induce a series of reactions with the H_2_O molecules in the airstream. This photohydrolysis process yields a complex mixture of molecules and ions, including OH, OH-, and O_2_-, that are released into the air and throughout the indoor space.

Importantly, as shown in numerous laboratory and real-world settings, this AP mixture renders VOCs, viruses, bacteria, and fungi inactive in the air and on surfaces [S4–S8 Files]. For example, AP has been used in multiple long-term (21- and 12-months) clinical studies reducing the number of hospital-acquired bacterial infections by greater than 70% [Trosch, K., *et al*., in press].

The AP technology was evaluated by the FDA and awarded clearance in a Class II medical device. The FDA evaluation process included efficacy testing against six challenge pathogens (*Staphylococcus epidermis*, *Erwinia herbicola*, *MS2*, *ϕ-X174*, *Aspergillus niger*, *Bacillus globigii*) and extensive monitoring to ensure there were no harmful byproducts (*e*.*g*., ozone, formaldehyde, acetaldehyde, VOCs) [S9–S12 Files]. However, there are no publications to date that directly test the hypothesis that the AP technology is safe for use in mammalian-occupied indoor spaces. During the study reported here, mice were continuously exposed for 90 days to levels of AP that are effective against viral, bacterial, and fungal pathogens [S10 File]. Clinical observations, including overt signs of adverse effects, changes in dietary/food consumption and body weight, along with any abnormal behavior, were recorded and compared between the mice in a control group and an AP-exposed group. In addition, the blood chemistry and hematology values were determined for both groups. Finally, the histology of the respiratory pathway tissues (nasal, larynx, lung) of mice from the two groups was examined by a board-certified pathologist. As detailed below, no differences between the AP-exposed and the control group were found, that is, the mice of both groups were healthy and normal during the 90-day experiment. Critically, this is the first and, to the authors’ knowledge, the only report that directly shows that the AP technology does not cause any negative effects.

## Materials and methods

The use of animals and all procedures were approved by the Institutional Animal Care and Use Committee (IACUC) of Comparative Biosciences, Inc., and strictly followed NIH and American Veterinary Medical Association Guidelines. Twelve 6–8 weeks old mice (6 males [21.3 ± 2.6 gr;]; 6 females [17.4 ± 1.3 gr], [C57BL]) were acquired from Charles River Laboratories (Wilmington, MA). The mice were acclimated for 3 days under standard housing conditions as defined in the NIH Guide for the Care and Use of Laboratory Animals, 8^th^ Edition. On the fourth day, 3 females and 3 males were placed in two separate animal care cages (28 cm x 18 cm x 11 cm) with a microisolator top. These mice were taken to the test room containing the AP system (Pure and Clean; ActivePure Manufacturing, Bristol, VA). The remaining 6 mice were separated into 2 cages as well (*i*.*e*., 3 males, 3 females) and placed in a control room. Each room was 334 cm long, 272 cm wide, and 274 cm high. All cages were placed 60 cm-120 cm above the floor on a multi-tiered shelfing rack such that there was no direct path of outside HEPA-filtered air from the supply vent into the cages. All mice received ventilation from the room’s environment and each room had 5 air exchanges per hour to keep the concentration of AP at therapeutic levels. The same HVAC system served both animal care rooms.

Mice were provided with water *ad libitum* and were fed each day via an attached feed bottle with Purina LabDiet 5001. Food consumption was assessed as either ‘all’, ‘some’, or ‘none’ of the daily portion of food. All cages were disinfected twice a week. Adequate bedding was provided at each clean out and was made of material conducive to burrowing. Paper neslets were provided for enrichment activity.

Clinical observations of the mice in the test and controls rooms, including any overt signs of adverse effects (*e*.*g*., vomiting, diarrhea, hair loss, coughing, sneezing), any signs of pain and or suffering, and any abnormal behavior (*e*.*g*., aggression, biting, lethargy, immobility), were recorded at least once daily by trained veterinary staff during the acclimation period and throughout the 90-day study. Since none of these was observed by staff, no interventions to alleviate any discomfort and or pain were needed (see Results). Individual body weights of all mice were recorded prior to study start, weekly thereafter, and at the termination of the study.

The AP-levels, in terms of number of negative ions/cc of air, temperature, and relatively humidity in each room was measured daily using an Ion Counter 3200 Pro II (Ion Trading, Tokyo, Japan). Ozone levels were measured using a Teledyne Ozone Analyzer (Model 4300; Teledyne API, San Diego).

At the conclusion of the 90-daystudy, mice were euthanized by trained veterinary staff as approved by the Comparative Bioscience’s IACUC with CO_2_ as described in the American Veterinary Medical Association and NIH guidelines in order to minimize any discomfort/suffering. After death was confirmed by veterinary staff, terminal blood was obtained by cardiac puncture. Hematology tubes containing EDTA and serum tubes containing SST were used to collect 0.25 mL blood samples. The determination of hematology and clinical chemistry values was conducted by Quality Veterinary Laboratory LLC., Davis, CA.

At necropsy, each mouse was perfused transcardially with 10% (v/v) neutral buffered formalin. Nasal, larynx and lung tissues from all mice were excised, trimmed, embedded in paraffin, and stained. All chemicals were reagent grade. The nasopharynx was stained using hematoxylin (H, [SL90-1]) and Eosin-Y (E, [SL98-1]) obtained from Statlab (Mckinney, TX.). The lungs and larynx were stained using a Dako Coverstainer (Agilent [San Diego, CA] with Dako hematoxylin [CS70] and Dako eosin [CS701]). The stained histology sections were examined using a light microscope by a board-certified pathologist.

Statistical analyses were done using Microsoft Excel and Prism version 7 GraphPad Software for unpaired t-test or Mann-Whitney test. For all analyses, P values <0.05 were considered statistically significant.

## Results

### Animal care rooms parameters measurements

The temperature, O_3_, humidity, and AP levels in the control room and the AP-containing room (test room) were measured and recorded daily as described in the Materials and methods. These results were averaged and are summarized in Table 1.

**Table 1:** Animal care room parameters.

	Temperature (°C)	Humidity (RH %)	Ozone (ppb)	AP Levels^a^
***Control Room***	21.5 ± 0.4	36.5 ± 7	4.3 ±3	4 ± 14
***Test Room***	21.3 ± 0.5	35.8 ± 6	5.1 ± 7	645 ± 106

The temperature, relative humidity, O_3_, and AP levels were recorded daily in both rooms, and the average of each value is presented. RH % is relative humidity percent, ppb is parts per billion,

^a^ indicates the AP levels in terms of negative ions/cc of air. ± indicates standard deviation.

Please note that the temperature, humidity, and ozone levels in test and control rooms were not significantly different (P = 0.37, 0.49, and 0.37, respectively). Importantly, the only significant difference between the two rooms was the AP levels between the test room containing the AP unit and the control room (P<0.001). The level of AP measured as ≥350 negative ions per cc of air has been shown to be therapeutic against viruses, bacteria, and fungi [S10 File]. It is important to note that the O_3_ levels in both rooms were not significantly different (P = 0.49), confirming previous results that the AP technology does not produce ozone [S11 and S12 Files].

### Clinical observations

Clinical observations, including overt signs of toxic or harmful effects, including, but not limited to, indications of pain or suffering and any abnormal behavior, were monitored by trained veterinary staff as described in the Materials and methods. There were no adverse clinical signs noted for any animal (*i*.*e*., in both the AP-exposed and control groups) over the entire 90-day course of this study. All animals were found to be healthy and normal.

### Body weights

**Fig 2:**
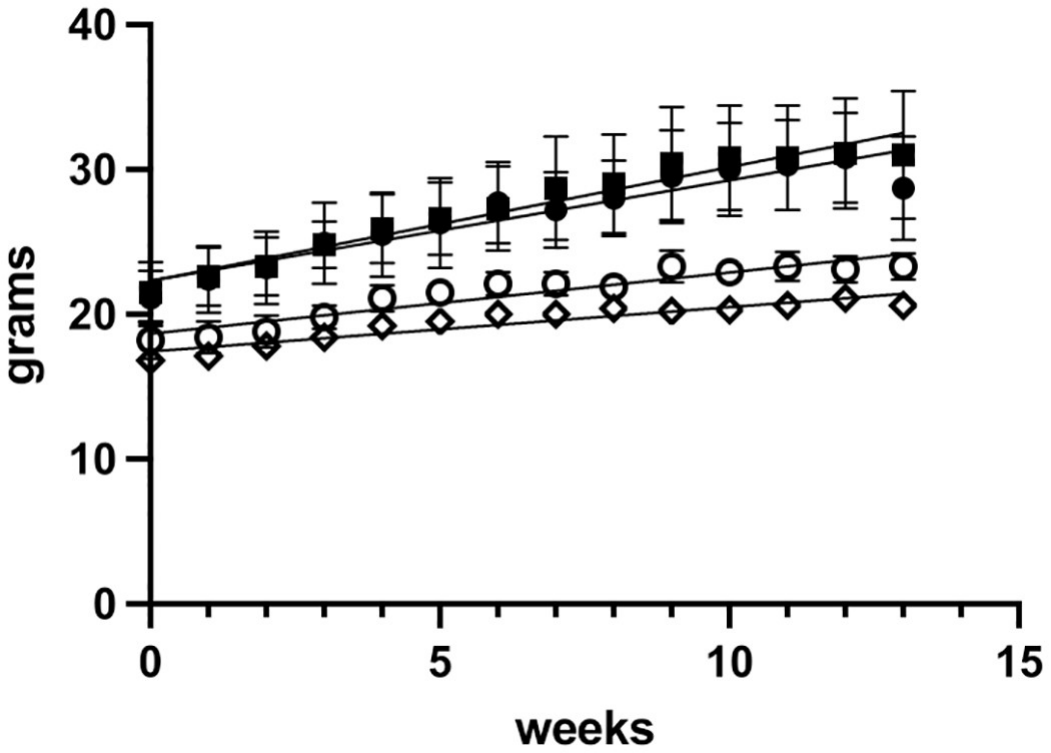
Weight gain of male and female mice during the 90-day study. The weights of male and female mice in the control and AP-exposed groups were determined weekly. Open symbols represent the weights of female mice (circles–control mice; diamonds—AP-exposed mice). Closed symbols represent the weights of male mice (circles–control mice; squares—AP-exposed mice). Error bars represent standard deviation (N = 3).

During the study, body weights were measured and recorded weekly as described in the Materials and methods. The weights of the male and female mice of the AP-exposed and control groups are presented in Fig 2.

It is important to note that AP levels did not affect the weight gain of either male or female mice.

Food consumption was qualitatively assessed daily as described in the Materials and methods. Both the control group and the AP-exposed mice showed normal food consumption during the 90-day study (results not presented). This is also supported by the consistent body weight gain of both groups over the course of the study as shown in Fig 2.

### Clinical chemistry and hematology analyses

At the conclusion of the 90-day study, terminal blood was collected and analyzed for hematology and clinical chemistry as described in the Materials and methods. The average clinical chemistry and hematology values in each group are shown in Tables 2 and 3, respectively.

**Table 2:** Clinical chemistry.

**Parameter**	**Unit**	**Control**	**Test**	**Parameter**	**Unit**	**Control**	**Test**
***ALB***	g/dL	2.6	2.5	***GLU***	mg/dL	213	229
***TP***	g/dL	4.4	4.3	***PHOS***	mg/dL	5.7	5.6
***ALP***	U/L	69.2	62.5	***TCO***_***2***_	mEq/L	20.3	21.3
***ALT***	U/L	31.5	31.8	***Na***	mEq/L	145	145
***AST***	U/L	89.2	75	***K***	mEq/L	8.2	8.3
***CK*** *	U/L	715	297	***Cl***	mEq/L	107	107
***TBIL***	mg/dL	0.2	0.1	***GLOB***	g/dL	1.8	1.8
***DBIL***	mg/dL	0	0	***A/G***	Ratio	1.5	1.4
***BUN***	mg/dL	23.3	22.5	***B/C***	Ratio	115	119
***CREAT***	mg/dL	0.2	0.2	***IBIL***	mg	0.1	0.1
***Ca***	mg/dL	8.9	8.9	***ANION***	mEq	25.7	24.3
***CHOL***	mg/dL	68.8	65.8				

At the conclusion of the 90-day study, the clinical chemistry values were determined from the blood of males and females of each group and averaged. Abbreviations: ALB, albumin; TP, total protein; ALP, alkaline phosphatase; ALT, alanine aminotransferase; AST, aspartate aminotransferase; CK, creatine kinase; TBIL, total blood bilirubin; DBIL, direct bilirubin; BUN, blood urea; CREAT, creatine; Ca, Calcium; CHOL, cholesterol; GLU, Glucose; PHOS, phosphate, TCO_2_, total carbon dioxide; Na, sodium; K, potassium; Cl, chloride; GLOB, globulin; A/G, total protein and albumin to globulin ratio; B/C, bilirubin to creatine ratio; IBIL, bilirubin; ANION, anion gap.

*The CK values from both groups were not statistically different (Mann-Whitney test: P = 0.43).

**Table 3:** Clinical hematology.

Parameter	Unit	Control	Test	Parameter	Unit	Control	Test
***WBC***	x10^3^/μL	5.7	4.7	***LYMPH***	%	90.8	89.3
***RBC***	x10^6^/μL	9	8.9	***MONO***	%	2.5	2
***HGB***	g/dL	12.9	12.8	***EOS***	%	2	4
***HCT***	%	44.9	43.8	***BASO***	%	0.3	0
***MCV***	fL	49.9	49.5	***NEUT***	x10^3^/μL	0	0
***MCH***	Pg	14.3	14.4	***LYMPH***	x10^3^/μL	0.3	0.2
***MCHC***	g/dL	28.7	29.1	***MONO***	x10^3^/μL	5.2	4.2
***RDW***	%	12.6	12.3	***EOS***	x10^3^/μL	0.1	0.1
***PLT***	x10^3^/μL	1067	1143	***BASO***	x10^3^/μL	0.1	0.2
***MPV***	fL	6.6	5.8	***RETIC***	%	0	0
***NEUT***	%	4.5	5.3	***RETIC***	x10^3^/μL	0	0

At the conclusion of the 90-day study, the hematology values were determined from the blood of male and female mice of each group and averaged. Abbreviations: WBC, white blood cells; RBC, red blood cells; HGB, hemoglobin; HCT, hematocrit; MCV, mean corpuscular volume; MCH, mean corpuscular hemoglobin; MCHC, mean corpuscular hemoglobin concentration; RDW, red cell distribution width; PLT, platelets; MPV, mean platelet volume; NEUT, neutrophils; LYMPH, lymphocytes; MONO, monocytes; EOS, eosinophils; BASO, basophils, RETIC, reticulocytes.

Analysis of these results showed that there were no significant differences (P values > 0.05 in all cases) between the AP-exposed and the control mice for both the blood chemistry and hematology values. In brief, all bloodwork indicated a population of normal animals of both groups.

### Histopathology of airway tissues

**Fig 3:**
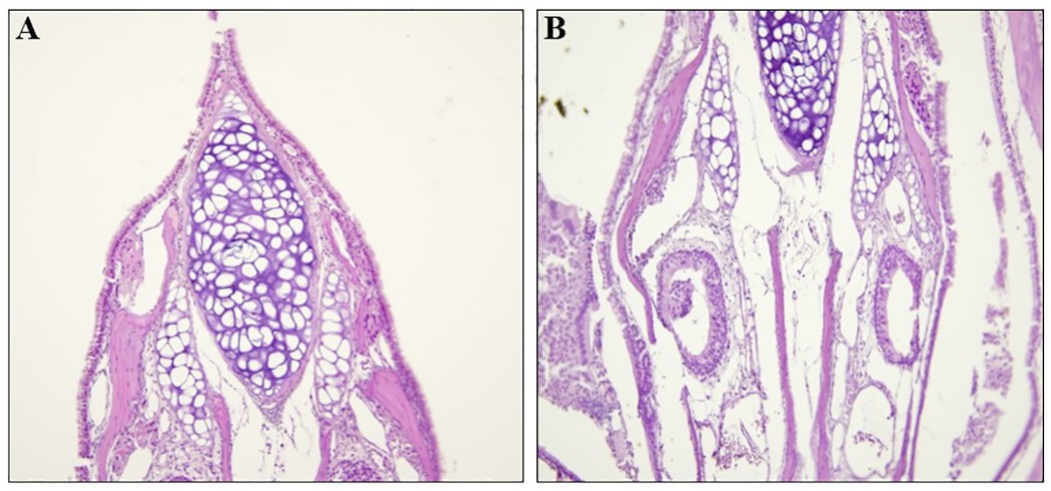
Histology of the nasal region of a control mouse and an AP-exposed mouse. The nasal regions of a control mouse (Panel A) and an AP-exposed mouse (Panel B) were processed and stained with H and E as described in the Materials and methods. The turbinate epithelium and cartilage in both Panel A and Panel B are within normal limits and there is no evidence of inflammation, erosion, luminal debris, or cellular proliferation in any of the tissues. 200X.

**Fig 4:**
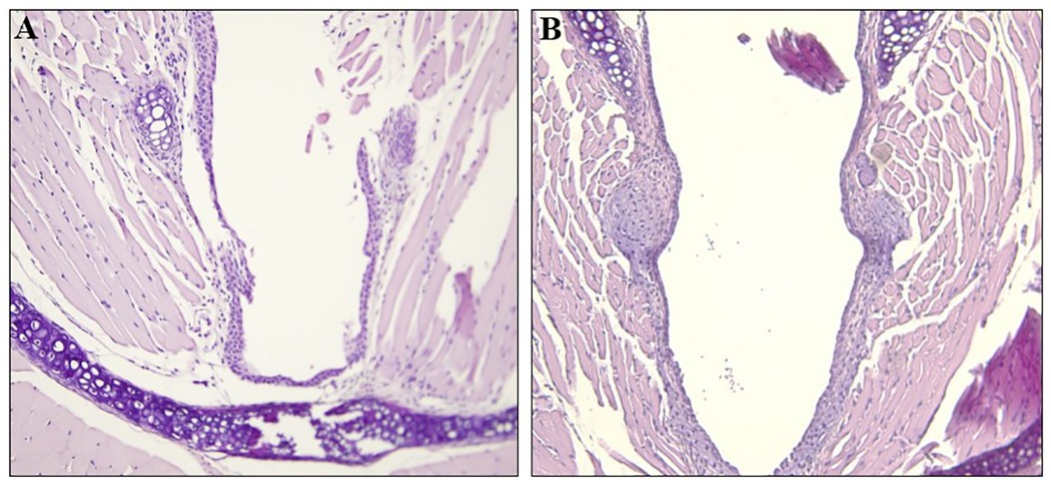
Histology of a portion of the larynx of a control mouse and an AP-exposed mouse. The larynx regions of a control mouse (Panel A) and an AP-exposed mouse (Panel B) were processed and stained with H and E as described in the Materials and methods. The laryngeal epithelium and underlying tissues in both Panel A and Panel B are within normal limits and there is no evidence of inflammation, erosion, luminal debris, cartilage damage, or cellular proliferation in any of the tissues. 200X.

**Fig 5:**
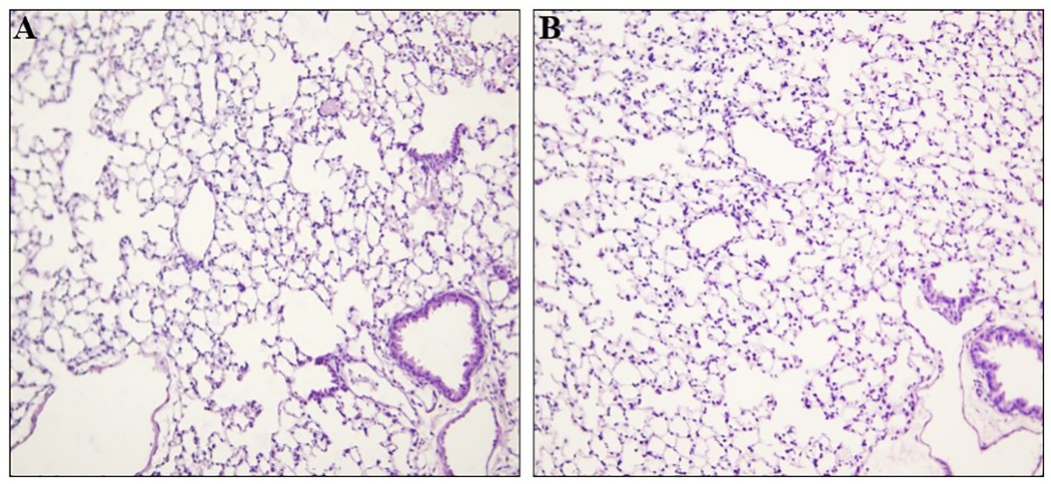
Histology of a portion of the lungs of a control mouse and an AP-exposed mouse. The lung region of a control mouse (Panel A) and of an AP-exposed (Panel B) was processed and stained with H and E as described in the Materials and methods. The bronchial epithelium, alveoli, interstitium, blood vessels, and serosa in both Panel A and Panel B are within normal limits and there is no evidence of erosion, inflammation, luminal debris, pneumonia, cellular proliferation or tumor formation in any of the lung tissues. 200X.

At necropsy, all mice were euthanized and nasal, larynx, and lung tissues were embedded in paraffin, sectioned, and stained as described in the Materials and methods. The stained histology sections were examined as described in the Materials and methods. Representative images are shown in Fig 3 of a control (panel A) and of an AP-exposed mouse (panel B) nasal region, Fig 4 from a control mouse (panel A) and an AP-exposed mouse (panel B) larynx, and Fig 5 of lung tissues from a control (panel A) and an AP-exposed (panel B) mouse.

Please note that none of the sections showed any abnormalities. There was no evidence of immune cells infiltration of any of the airway tissues (*e*.*g*., Dust cells in the alveolar tissues, lymphocyte infiltration [including neutrophils and eosinophils]). Note the lack of alveolar collapse, intra-alveolar edema, inflammation, and fibrotic remodeling in Fig 5 (Panel A: control) and Fig 5 (Panel B: AP-exposed). In brief, each histological section from the each of the control mice and the AP-exposed mice did not reveal any morphological differences between the airways of mice exposed to control conditions and the airways of mice exposed to therapeutic levels of AP.

## Discussion

This study was the first to assess the safety of Advanced Photohydrolysis in occupied indoor spaces by measuring the effects of therapeutic levels of Advanced Photohydrolysis on the health and wellbeing of mice. The use of mice as a model system for a number of human diseases has been well documented [for example, 23–28]. The advantages of mice include the physiological similarities between species (including humans), low cost, many strains available, and detailed guidelines for care of the mice in experimental studies [27]. C57BL mice were chosen for this study since this strain is inbred, sensitive to air-borne odors, has robust local allergy and inflammatory responses, and is widely used as a model of human disease [for example, please see 28, 29].

Male and female mice were assigned to identical animal care rooms except for the presence of therapeutic levels of AP (please see Table 1). For example, factors (*e*.*g*., food, vibration, noise, lighting, male and female in one cage) that could have induced unintentional stress to the mice were identical thereby reducing confounding variables. Air currents and traffic patterns were similar, so the level of exposure to circulating air was similar in both groups. Moreover, the animal care cages were placed so that each mouse was not exposed directly to outside HEPA-filtered air that did not contain therapeutic levels of AP. Thus, any changes to the health, well-being, hematology and blood chemistry markers, and histopathology of the airways of AP-exposed mice in contrast to those of the control group mice would have been attributed to the presence of therapeutic levels of AP.

Yet, as described in the Results above, there were no indications of pain or distress, no differences in the behavior, food consumption and weight gain (please see Fig 2) between the control group mice and the AP-exposed group mice during the 90-day study. The mice of both groups were found to be normal and healthy throughout the 90-day study.

In addition, if AP-levels had affected hepatocellular function, then the levels of alanine aminotransferase and aspartate aminotransferase of the AP-exposed group mice would have been expected to be altered from those of the control group [30]. No such differences were observed (please see Table 2). Additionally, the levels of alkaline phosphatase and total bilirubin were similar for both groups of mice indicating that hepatobiliary function [31, 32] was not affected. Other markers of cellular and organ function, including albumin, calcium, chloride, total cholesterol, creatine, globulin, glucose, phosphorous, potassium, total protein, anion gap, and urea nitrogen were not significantly different between the control and AP-exposed mice (Table 2). The clinical hematology results (please see Table 3) were unremarkable in that the levels of red blood cells, the morphology of red blood cells, hematocrit, and the numbers and types of white blood cells including platelets, were not different from control and test group mice. Taken together, the blood chemistry and clinical hematology results did not reveal any direct or indirect negative effects of 90-day exposure to therapeutic levels of AP.

It is well understood that the mammalian respiratory system is divided into a conduction portion, which includes the nose, the pharynx, the larynx, the trachea, and the bronchi, and a respiratory portion which contains the respiratory bronchioles, alveolar ducts, and alveoli [33]. Each portion has several morphological and functionally distinct cell types [33]. In addition, each region responds to environmental insults with different responses. For example, cells within the epithelium of the upper airway are highly innervated [34–39] with sensory neurons that induce protective reflexes, such as coughing and sneezing, and cell changes when noxious stimuli are detected (*e*.*g*., congestion and fibrosis in alveoli and terminal/respiratory bronchioles [40–44]. If AP had had any negative effects on the respiratory system, then these would be evident in an increase in protective reflexes and in an examination of the epithelium and underlying tissues of the upper and lower portions of the respiratory system. However, as shown in the Results, there were no increases in protective reflexes, and no differences in the histology of the conduction and respiratory portions of the airway tissues of mice exposed to therapeutic levels of Advanced Photohydrolysis to those of the control mice (please see Figs 3 through 5). For example, the epithelial tissues of the nasal vestibule, the keratinized stratified squamous epithelium, the pseudostratified columnar ciliated epithelium [45], and the underlying tissues were identical in both groups. There was no evidence of necrosis, lymphocyte hyperplasia (*e*.*g*., monocyte or macrophage infiltration), edema, or inflammation in any of the airway epithelia. or subjacent tissues Additionally, no increase in goblet cell numbers (often a response to airway irritation and injury [46]), no changes to the appearance and numbers of Club cells (which play a role in the biotransformation of many harmful compounds [47]) and no fibrosis of lung tissues. Also, there was no evidence of alveolar congestion (please compare Fig 4 panel A to panel B). Each of these would have been expected to be altered if AP-levels had had a negative effect. However, since none of these was found, it can be concluded that therapeutic levels of AP did not result in any deleterious effects on either the upper or lower airway tissues.

### Summary and conclusions

The results of this 90-day study convincingly showed that there were no differences between mice exposed to therapeutic levels of Advanced Photohydrolysis and control mice with respect to: Behavior (a marker for the general health of mice); weight gain; adverse clinical effects; protective reflexes; blood chemistry; hematology; and, critically, the histology of the upper and lower airway tissues.

From this preponderance of results, it is clear that therapeutic levels of Advanced Photohydrolysis had no negative effects on the exposed mice. It is important to stress that this is the first report that directly tests and confirms the safety of the Advanced Photohydrolysis technology. While it is not possible to directly extrapolate these findings and conclusions to humans, this study provides the foundation for, and encourages, additional experiments beyond the scope and intent of this study. These may include examination of other tissues (*e*.*g*., skin, eyes), other animal models (*e*.*g*., porcine), detailed molecular studies of immune cells (*e*.*g*., T_reg_ and T_helper_), cytokines (*e*.*g*., IL-1β, IL-6, and many others), and the histochemistry of airway tissues for inflammatory markers (*e*.*g*., TNF-α).

## Supporting information

S1 File US patent D985,100 S. (PDF)

S2 File US patent 10,391,193 B2. (PDF)

S3 File US patent D974,538 S. (PDF)

S4 File VOC testing. (PDF)

S5 File Bioaerosols 1. (PDF)

S6 File Bioaerosols 2. (PDF)

S7 File MSICU testing. (PDF)

S8 File CVICU testing. (PDF)

S9 File FDA testing. (PDF)

S10 File Ion concentration and efficacy. (PDF)

S11 File Ozone Testing 1. (PDF)

S12 File Ozone Testing 2. (PDF)
