# Supplementary material for: Evaluate the safety of a novel photohydrolysis technology used to clean and disinfect indoor air: A murine study
Source: PLoS One. 2024 Oct 9;19(10):e0307031. doi: 10.1371/journal.pone.0307031 (PMC11463749; doi:10.1371/journal.pone.0307031)
Supplement: S1 File — (PDF) [file pone.0307031.s001.pdf]

US00D985110S

(12) **United States Design Patent** (10) **Patent No.: US D985,110 S**
Recer et al. (45) **Date of Patent: ** May 2, 2023**

(54) **DEVICE FOR FRESHENING INTERIOR AIR**

(71) Applicant: **DBG Group Investments, LLC**, Dallas, TX (US)

(72) Inventors: **Chad Recer**, Frisco, TX (US); **Deborah Jessup**, Pittsburgh, PA (US); **Joe Urso**, Dallas, TX (US); **Andrew Eide**, Rockwall, TX (US)

(**) Term: **15 Years**

(21) Appl. No.: **29/721,805**

(22) Filed: **Jan. 23, 2020**

(51) **LOC (14) Cl.** **23-01**

(52) **U.S. Cl.**
USPC **D23/364**

(58) **Field of Classification Search**
USPC D23/200, 213, 325–326, 332, 314–324, D23/327–331, 419, 299, 354–365
CPC A61L 9/16; A61L 9/22; B01D 46/2414; B03C 3/155; F24F 3/16; F24F 13/222; F24F 11/30
See application file for complete search history.

(56) **References Cited**

U.S. PATENT DOCUMENTS

5,035,728	A *	7/1991	Fang	B03C 3/32 422/120
D323,382	S *	1/1992	Wang	D23/335
D333,866	S *	3/1993	Wang	D23/335
D373,414	S *	9/1996	Bucher	D23/335
D518,160	S *	3/2006	Normark	D23/366
D526,058	S *	8/2006	Blateri	D23/335
D534,629	S *	1/2007	Bucher	D23/335
D570,464	S *	6/2008	Normark	D23/364
D601,687	S *	10/2009	Chan	D23/364
D754,628	S *	4/2016	Baird	D14/204
D862,674	S *	10/2019	Shin	D23/364
D862,675	S *	10/2019	Shin	D23/364
D935,589	S *	11/2021	Lu	F24F 1/02 D23/364
D948,008	S *	4/2022	O'Brien	D23/325
D952,826	S *	5/2022	Zhan	D23/364
D953,503	S *	5/2022	Zheng	D23/359
2020/0149755	A1*	5/2020	Yan	F24F 1/02
2020/0182494	A1*	6/2020	Fitsch	B01D 33/073

OTHER PUBLICATIONS

Chinese Design Application No. 2019300144232 filed Jan. 11, 2019; Patentee Guangdong Haikebao Environmental Technology Co., Ltd.
Office Action received for Mexican Patent Application No. MX/f/2020/001663, dated Oct. 27, 2021, 14 pages.

* cited by examiner

Primary Examiner — Dana K Weiland
Assistant Examiner — Jonathan E Fiencke
(74) *Attorney, Agent, or Firm* — Workman Nydegger; Logan Christenson; John Guynn

(57) **CLAIM**

The ornamental design for a device for freshening interior air, as shown and described.

DESCRIPTION

FIG. **1** illustrates a perspective view of an air freshening device showing the new design;
FIG. **2** illustrates a front elevation view thereof;
FIG. **3** illustrates a rear elevation view thereof;
FIG. **4** illustrates a top plan view thereof;
FIG. **5** illustrates a bottom plan view thereof;
FIG. **6** illustrates a right side elevation view thereof; and,
FIG. **7** illustrates a left side elevation view thereof.
In the drawings, the broken lines and unshaded areas depict portions of the device for freshening interior air that form no part of the claimed design.

1 Claim, 7 Drawing Sheets

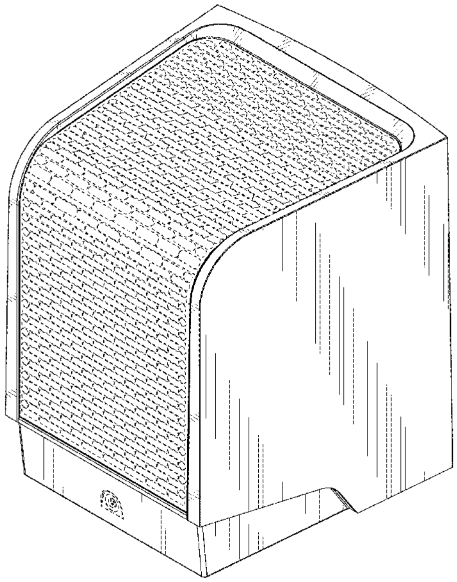

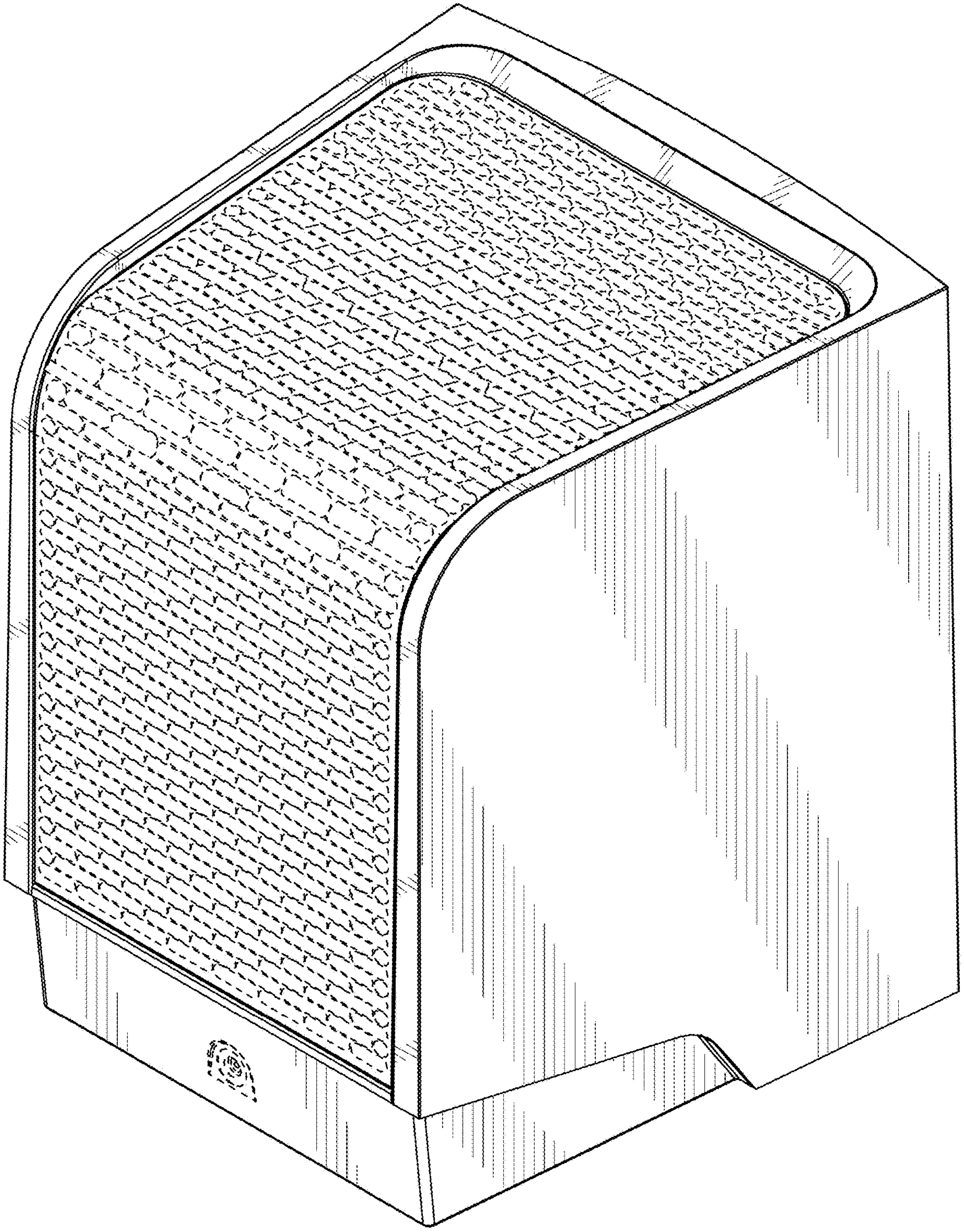

FIG. 1

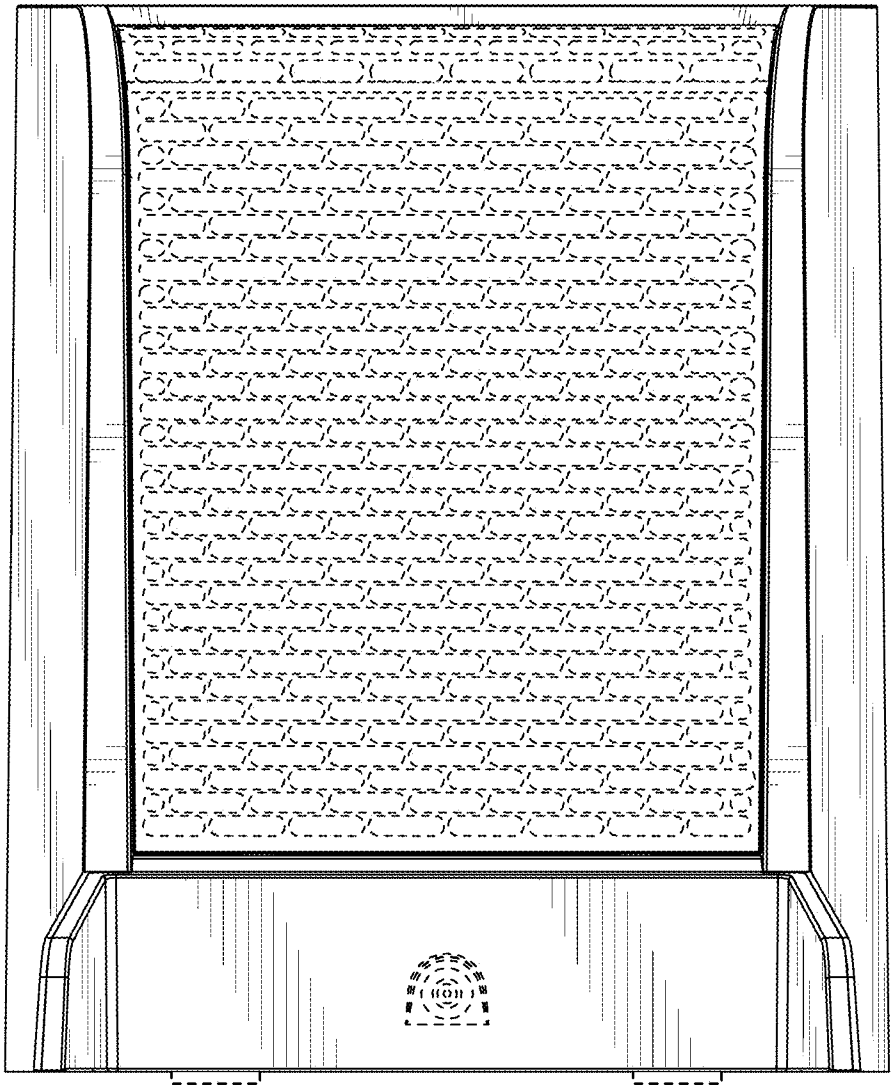

FIG. 2

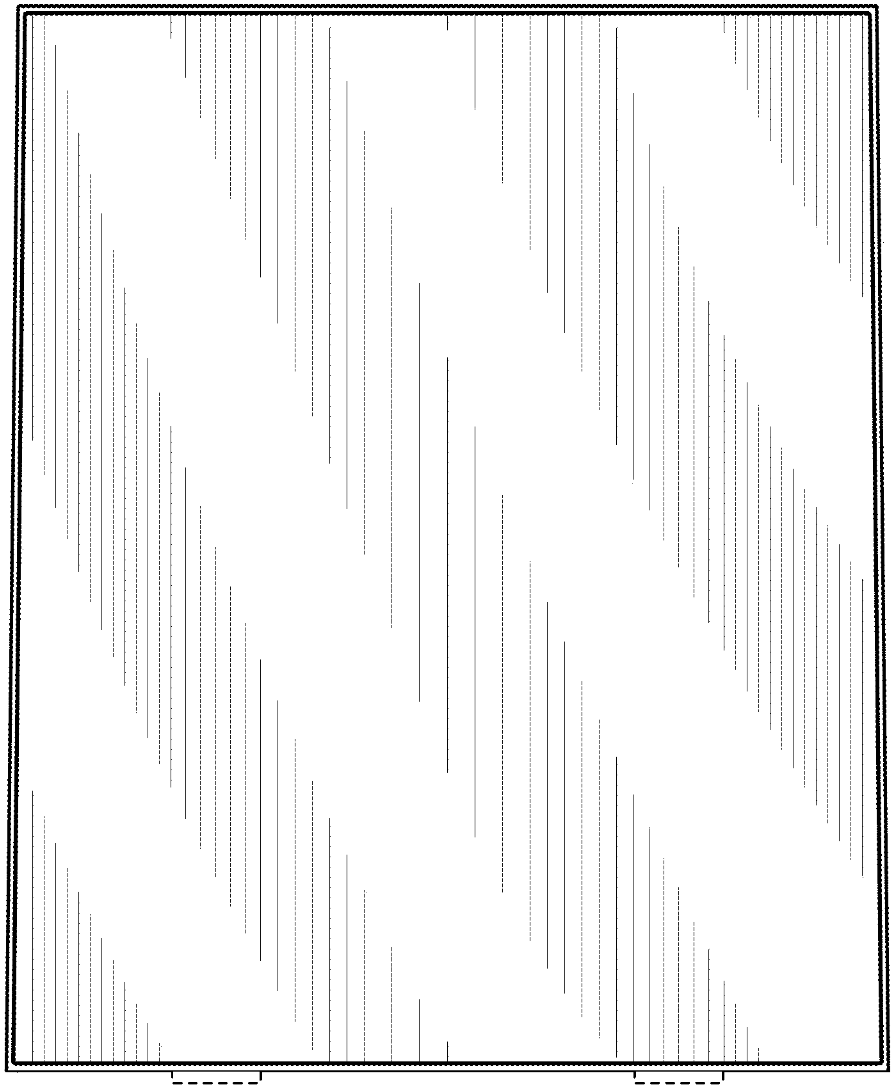

FIG. 3

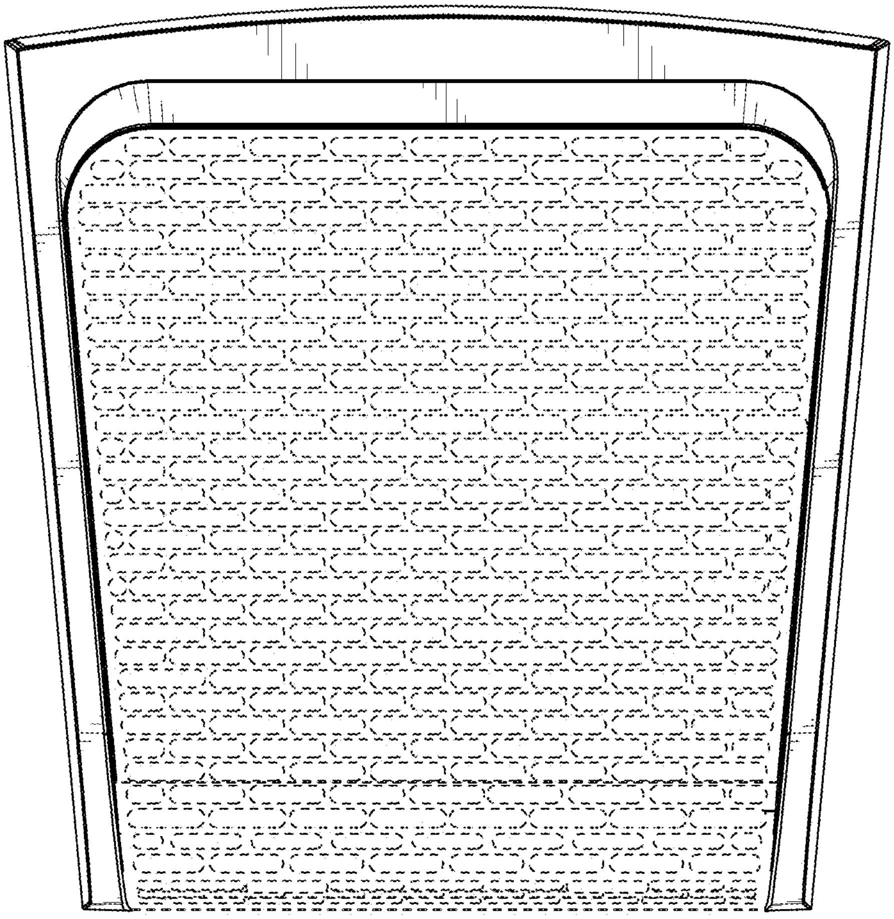

FIG. 4

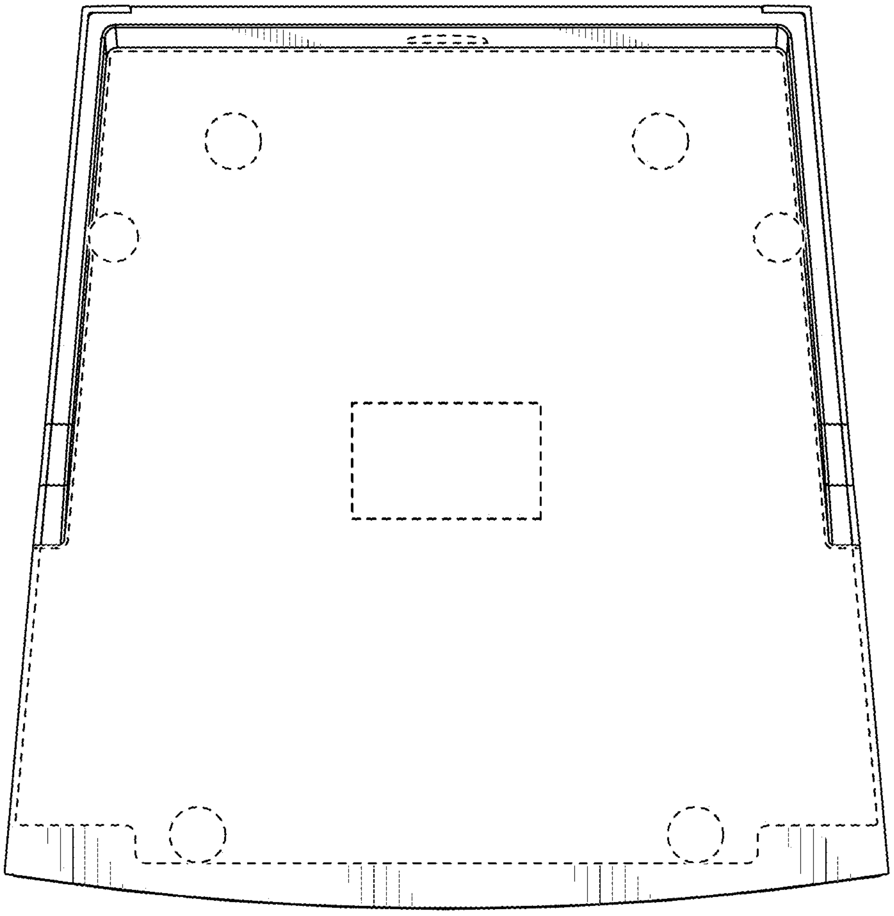

FIG. 5

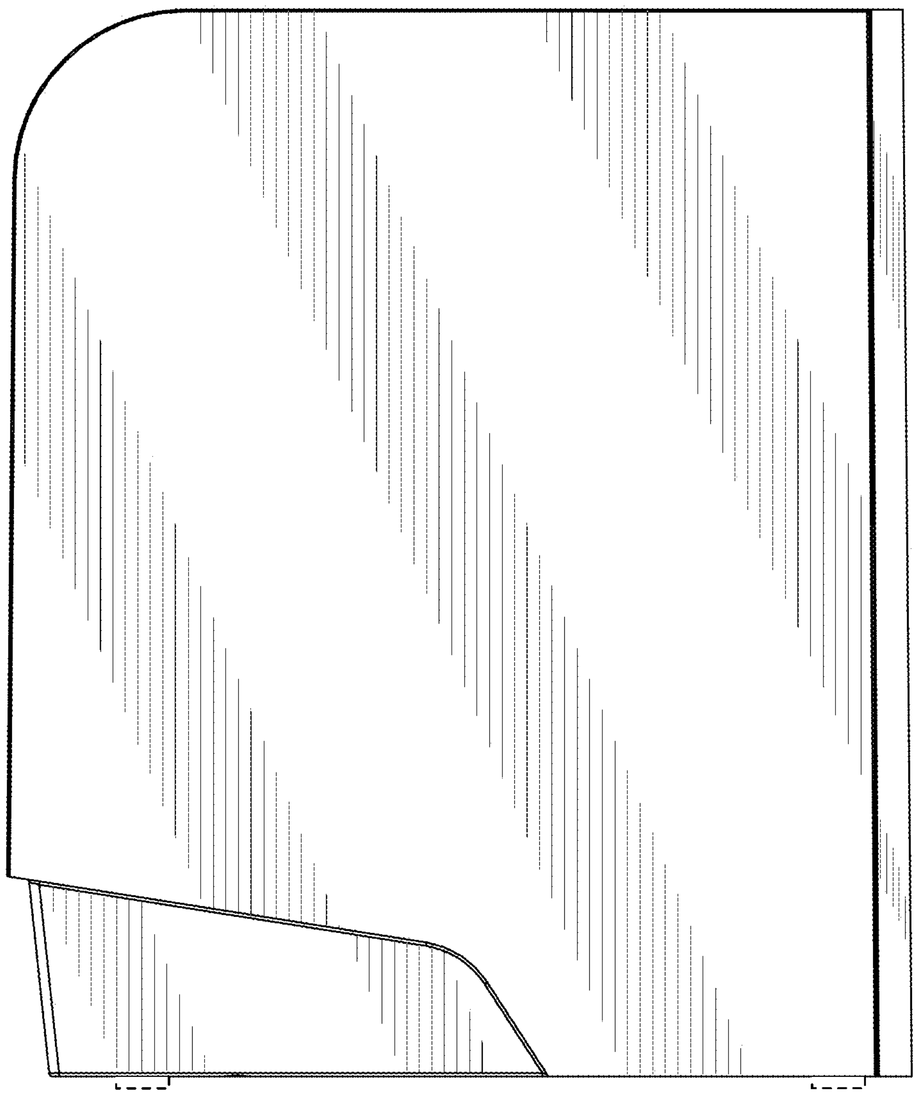

FIG. 6

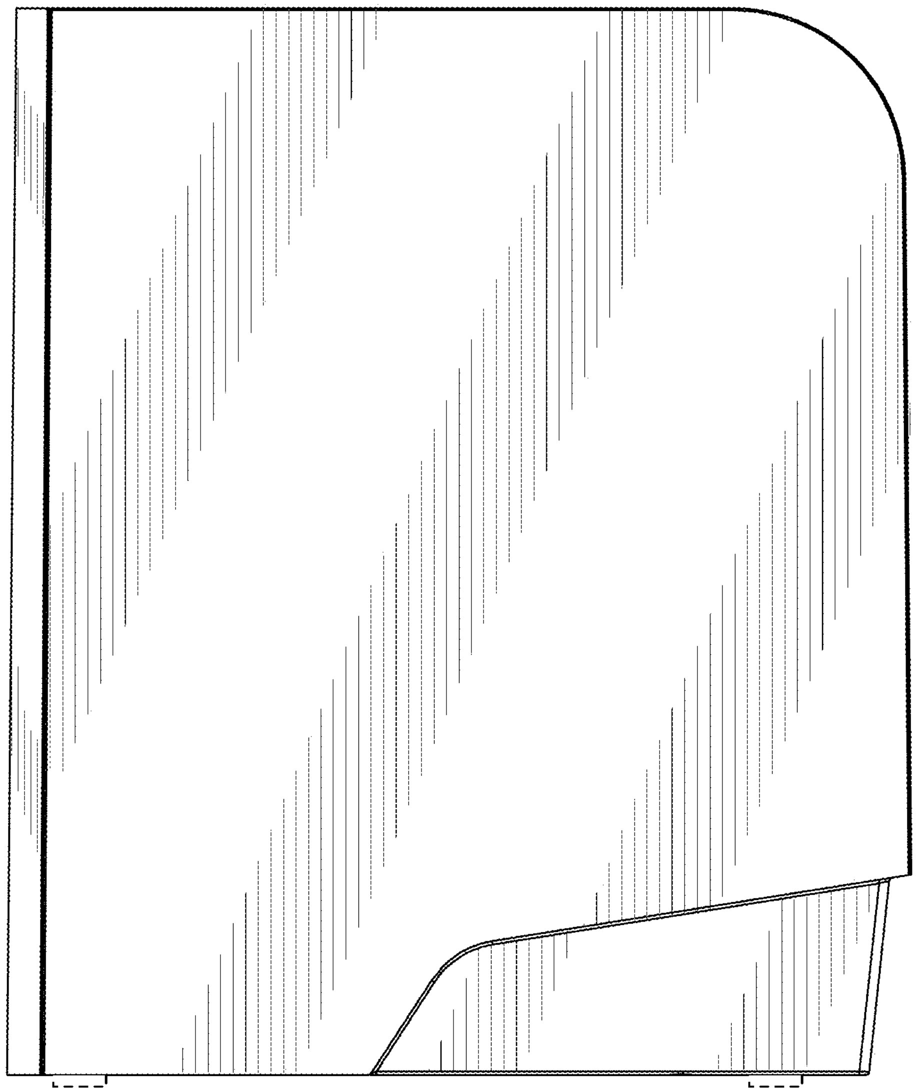

FIG. 7
