# Supplementary material for: Evaluate the safety of a novel photohydrolysis technology used to clean and disinfect indoor air: A murine study
Source: PLoS One. 2024 Oct 9;19(10):e0307031. doi: 10.1371/journal.pone.0307031 (PMC11463749; doi:10.1371/journal.pone.0307031)
Supplement: S2 File — (PDF) [file pone.0307031.s002.pdf]

US010391194B2

(12) **United States Patent**
Eide

(10) **Patent No.: US 10,391,194 B2**
(45) **Date of Patent: Aug. 27, 2019**

(54) **ACTIVE PHOTOCATALYTIC OXIDATION**

(71) Applicant: **DBG GROUP INVESTMENTS LLC**, Dallas, TX (US)

(72) Inventor: **Andrew Eide**, Rockwall, TX (US)

(73) Assignee: **DBG GROUP INVESTMENTS, LLC**, Dallas, TX (US)

(*) Notice: Subject to any disclaimer, the term of this patent is extended or adjusted under 35 U.S.C. 154(b) by 0 days.

(21) Appl. No.: **15/835,363**

(22) Filed: **Dec. 7, 2017**

(65) **Prior Publication Data**

US 2018/0104376 A1 Apr. 19, 2018

Related U.S. Application Data

(60) Division of application No. 14/705,046, filed on May 6, 2015, now Pat. No. 9,867,897, which is a continuation-in-part of application No. 13/602,102, filed on Sep. 1, 2012, now Pat. No. 9,623,374.

(51) **Int. Cl.**
A61L 9/20 (2006.01)
B01D 53/88 (2006.01)

(52) **U.S. Cl.**
CPC ***A61L 9/205*** (2013.01); ***B01D 53/885*** (2013.01); *B01D 2255/20707* (2013.01); *B01D 2255/802* (2013.01); *B01D 2258/06* (2013.01); *B01D 2259/804* (2013.01)

(58) **Field of Classification Search**
CPC . A61L 2/088; A61L 2/10; A61L 9/205; A61L 2209/212
See application file for complete search history.

(56) **References Cited**

U.S. PATENT DOCUMENTS

6,238,631	B1	5/2001	Ogata
7,425,226	B2	9/2008	Powell
7,674,436	B1	3/2010	Felman
8,658,101	B1	2/2014	Burnett

(Continued)

FOREIGN PATENT DOCUMENTS

DE	1965022	7/1970
JP	2000262605	9/2000

(Continued)

OTHER PUBLICATIONS

Machine translation of JP 2003287354 (Year: 2003).*

(Continued)

Primary Examiner — Timothy C Cleveland
(74) *Attorney, Agent, or Firm* — Workman Nydegger

(57) **ABSTRACT**

An active oxidation and purifying system is provided to increase or maximize the rate of photocatalytic oxidation and ambient air purification capacity by providing both direct ultraviolet (UV) light and reflected UV light directed to the surface and apertures of active cell panels coated with a photocatalytic material. In one example, the active cells also include a plurality of apertures disposed in a transverse manner from the first surface to the second surface of the active cell. Furthermore, a first set of the apertures could be disposed about 45 degrees relative to a median axis along the first and second surfaces, while a second set of apertures could be disposed about negative 45 degrees relative to the same median axis in order to increase the surface area impinged by the direct and reflected UV light.

18 Claims, 14 Drawing Sheets

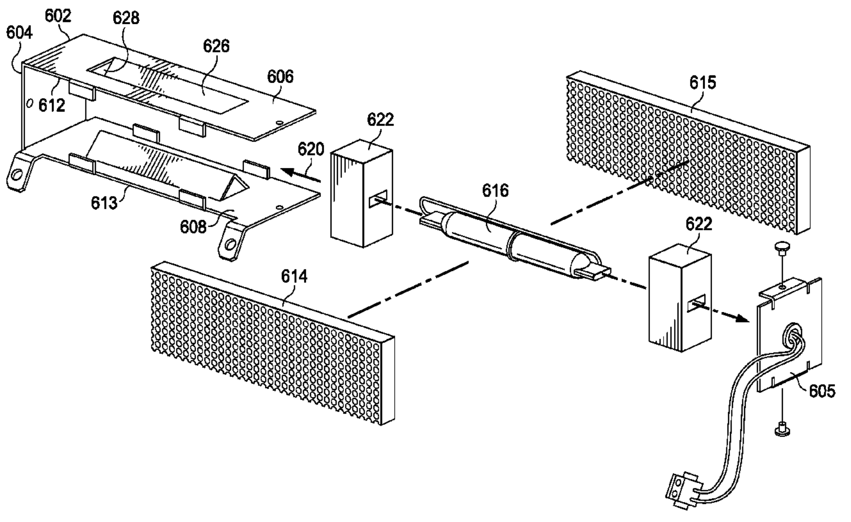

(56) **References Cited**

U.S. PATENT DOCUMENTS

9,011,780	B1	4/2015	Burnett
9,623,374	B2	4/2017	Eide
9,867,897	B2	1/2018	Eide
2006/0057020	A1	3/2006	Tufo
2006/0262389	A1	11/2006	Zaczek
2009/0035176	A1	2/2009	Normark et al.
2009/0041617	A1	2/2009	Lee
2012/0000860	A1	1/2012	Arenshtam
2012/0315184	A1	12/2012	Clark
2014/0050611	A1	2/2014	Warren et al.
2014/0065023	A1	3/2014	Eide
2014/0091230	A1	4/2014	Clark et al.
2018/0104375	A1	4/2018	Eide

FOREIGN PATENT DOCUMENTS

JP	2003135973	5/2003
JP	2003284926	10/2003
JP	2003287354	10/2003
JP	2006017358	1/2006
JP	2007130042	5/2007
WO	2009038236	3/2009
WO	WO 2012/033818	3/2012
WO	WO 2013/036553	3/2013

OTHER PUBLICATIONS

PCT: International Search Report and Written Opinion of PCT/US15/33364 (related application); dated Sep. 4, 2015; 16 pgs.
U.S. Appl. No. 13/602,102, Dec. 13, 2016, Notice of Allowance.
U.S. Appl. No. 13/602,102, May 2, 2016, Office Action.
U.S. Appl. No. 13/602,102, Nov. 12, 2015, Office Action.
U.S. Appl. No. 13/602,102, Apr. 9, 2015, Office Action.
U.S. Appl. No. 13/602,102, Oct. 9, 2014, Office Action.
U.S. Appl. No. 13/602,102, Mar. 11, 2014, Office Action.
U.S. Appl. No. 14/705,046, Nov. 13, 2017, Notice of Allowance.
U.S. Appl. No. 14/70,5046, Jul. 18, 2017, Office Action.
Supplementary European Search Report issued in PCT/US2015033364 dated Dec. 7, 2018.

* cited by examiner

FIG. 1A

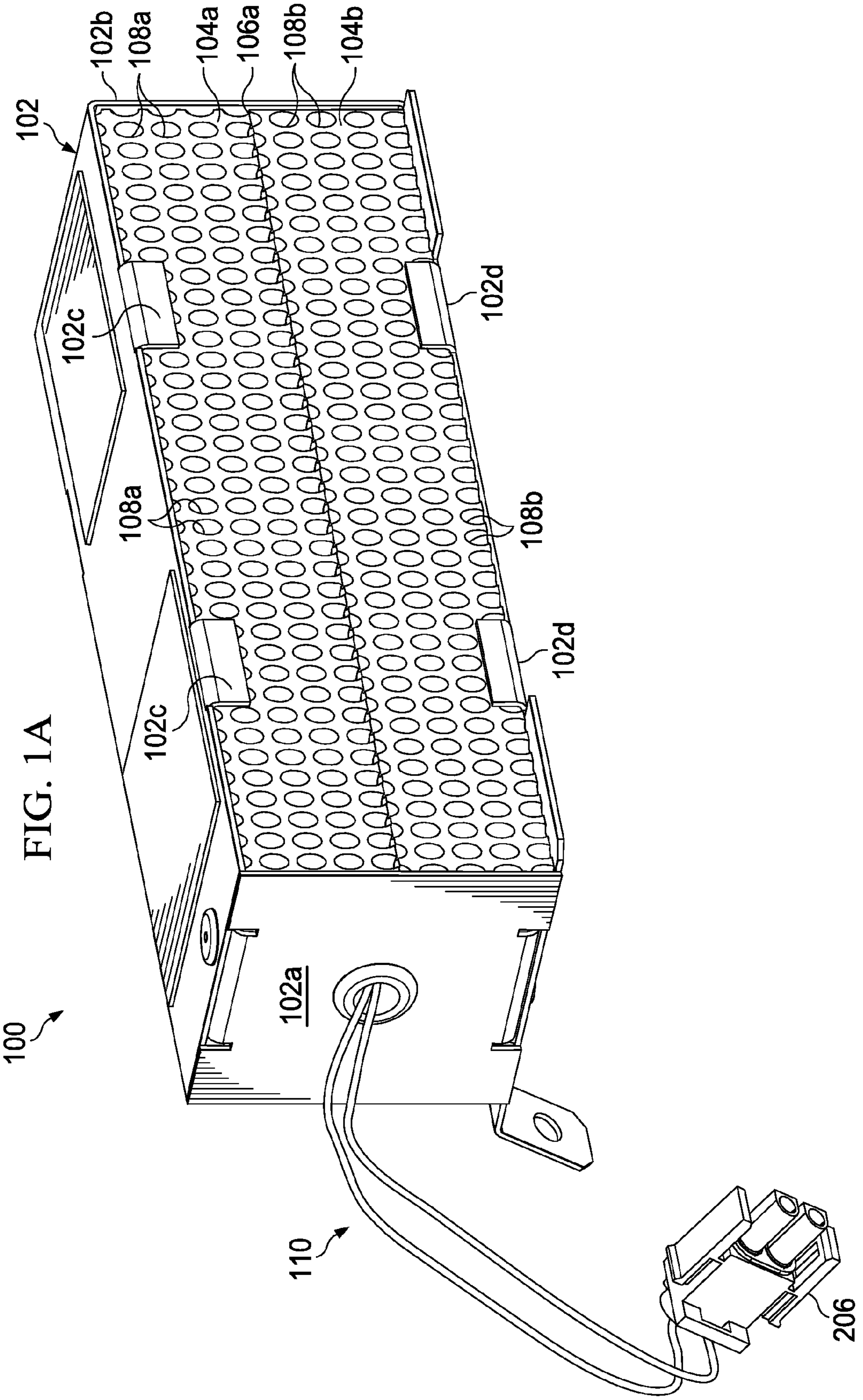
102
102b
108a
104a
106a
108b
104b
102c
102d
108a
108b
102c
102d
100
102a
110
206

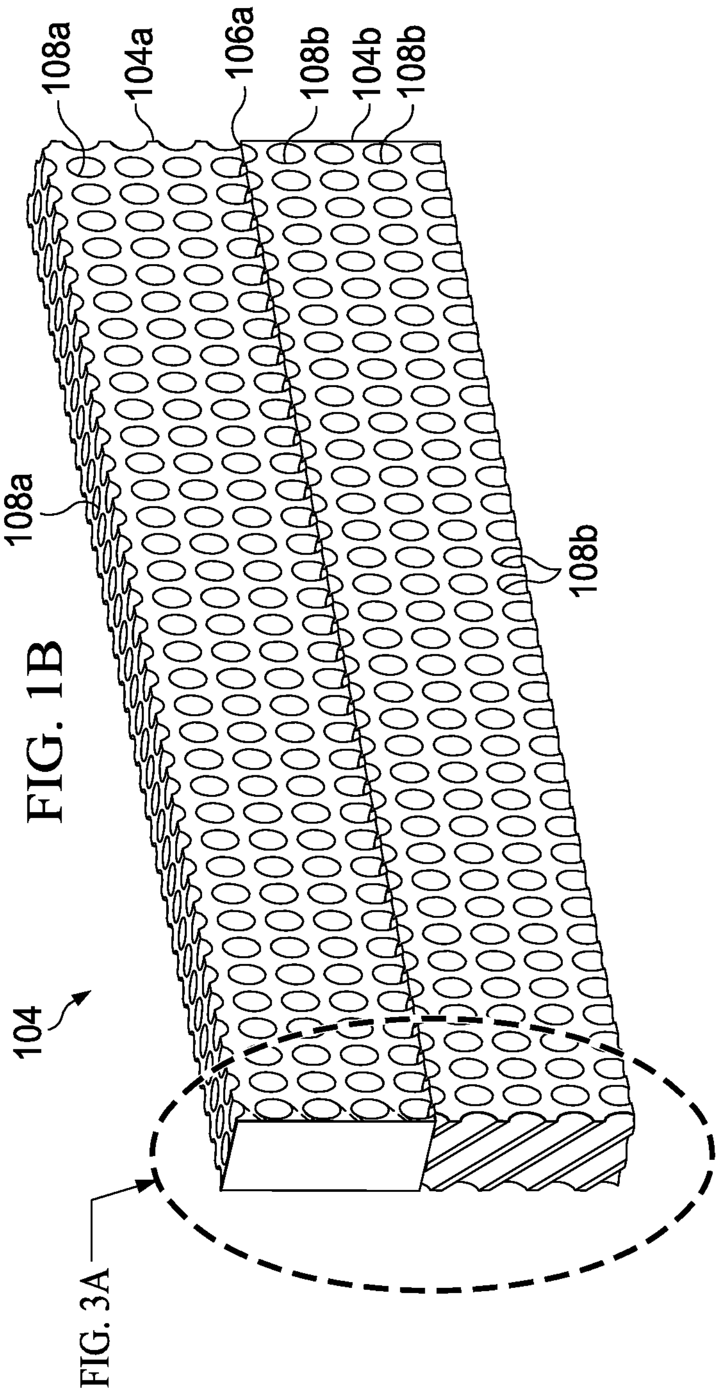

FIG. 1B

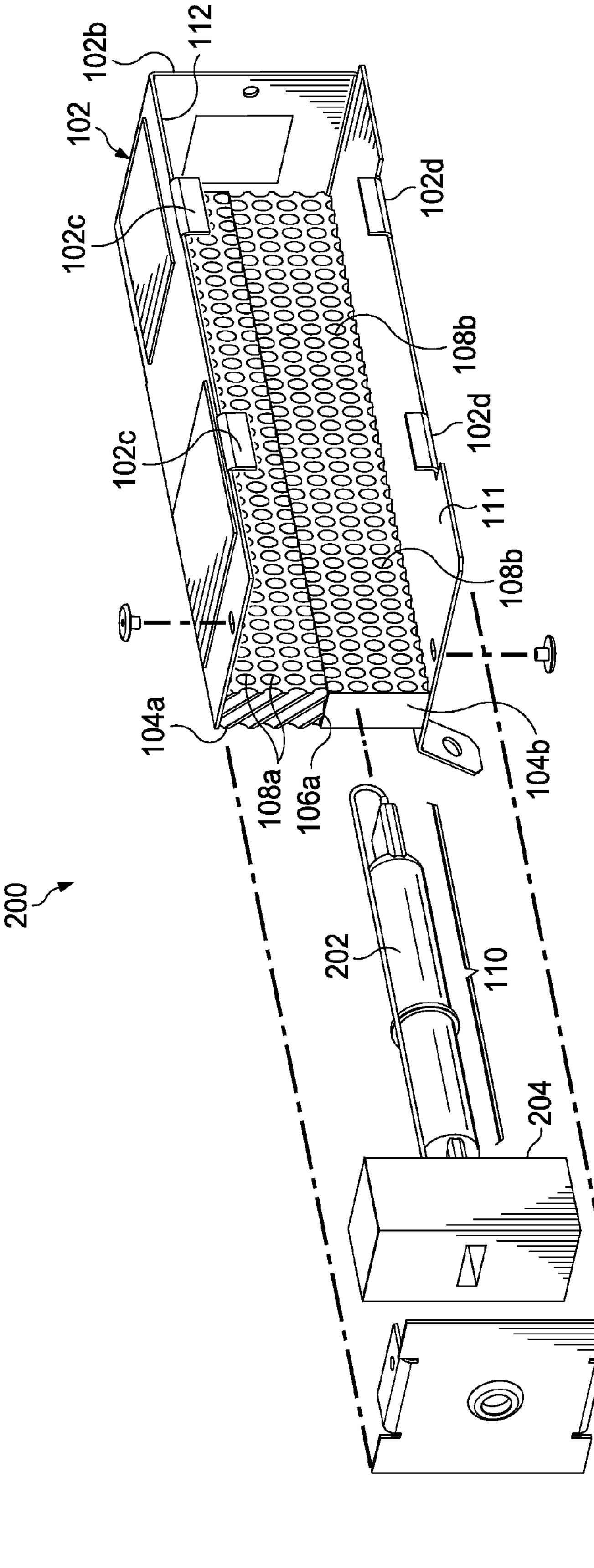

102b
112
102
102c
102d
108b
102c
102d
111
108b
104a
108a
106a
104b
200
202
110
204

FIG. 2

FIG. 3A

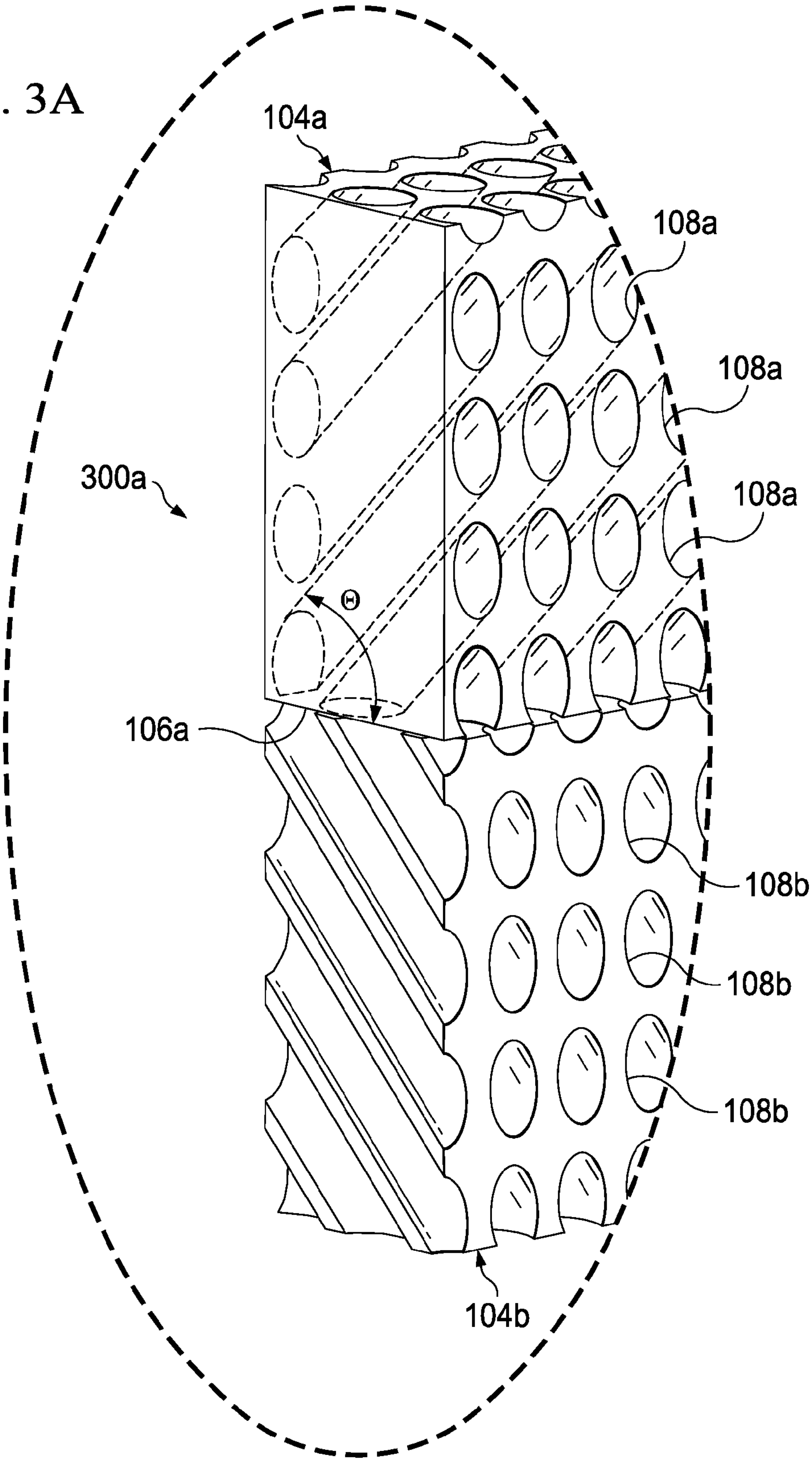

FIG. 3B

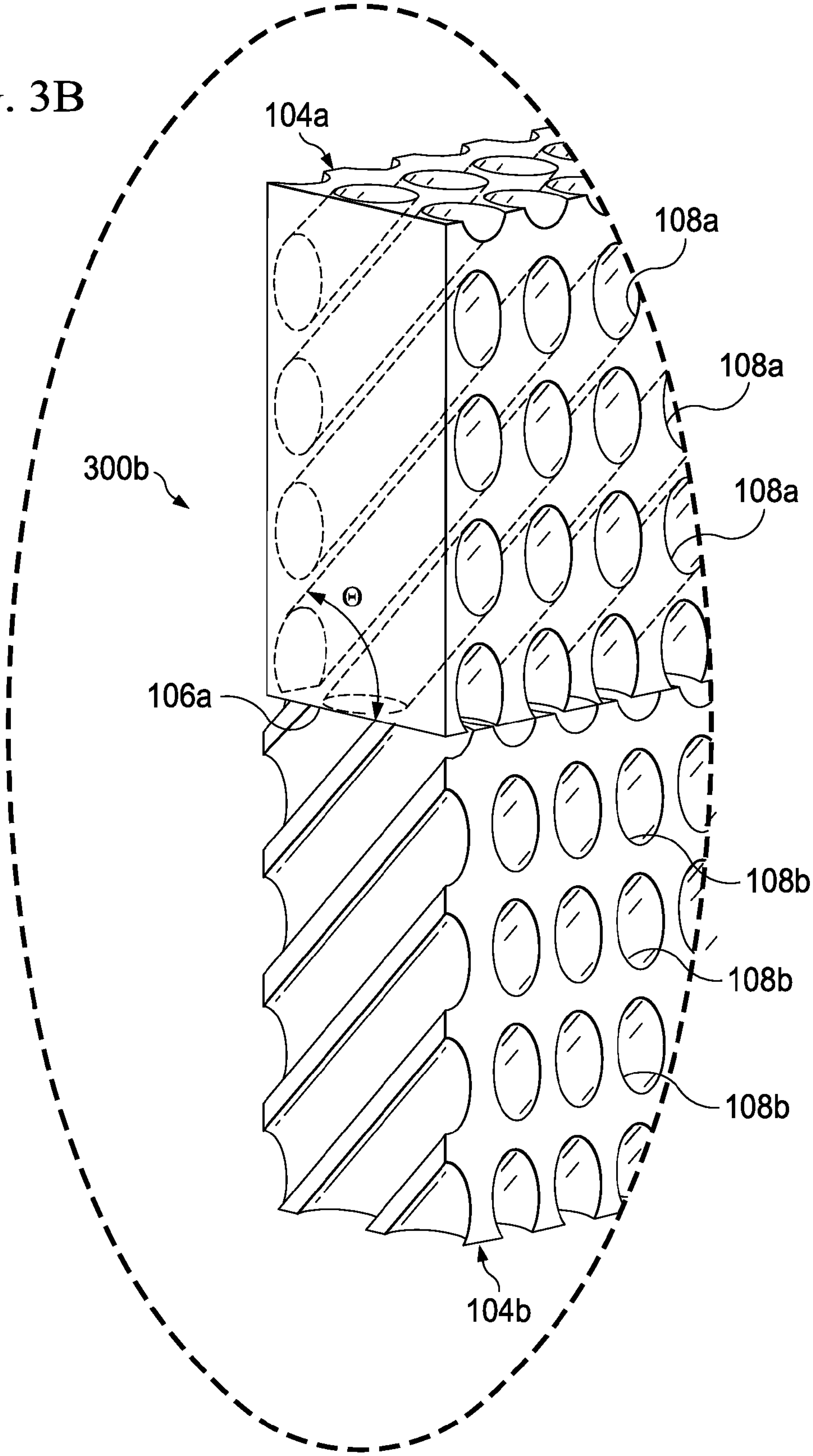

FIG. 3C

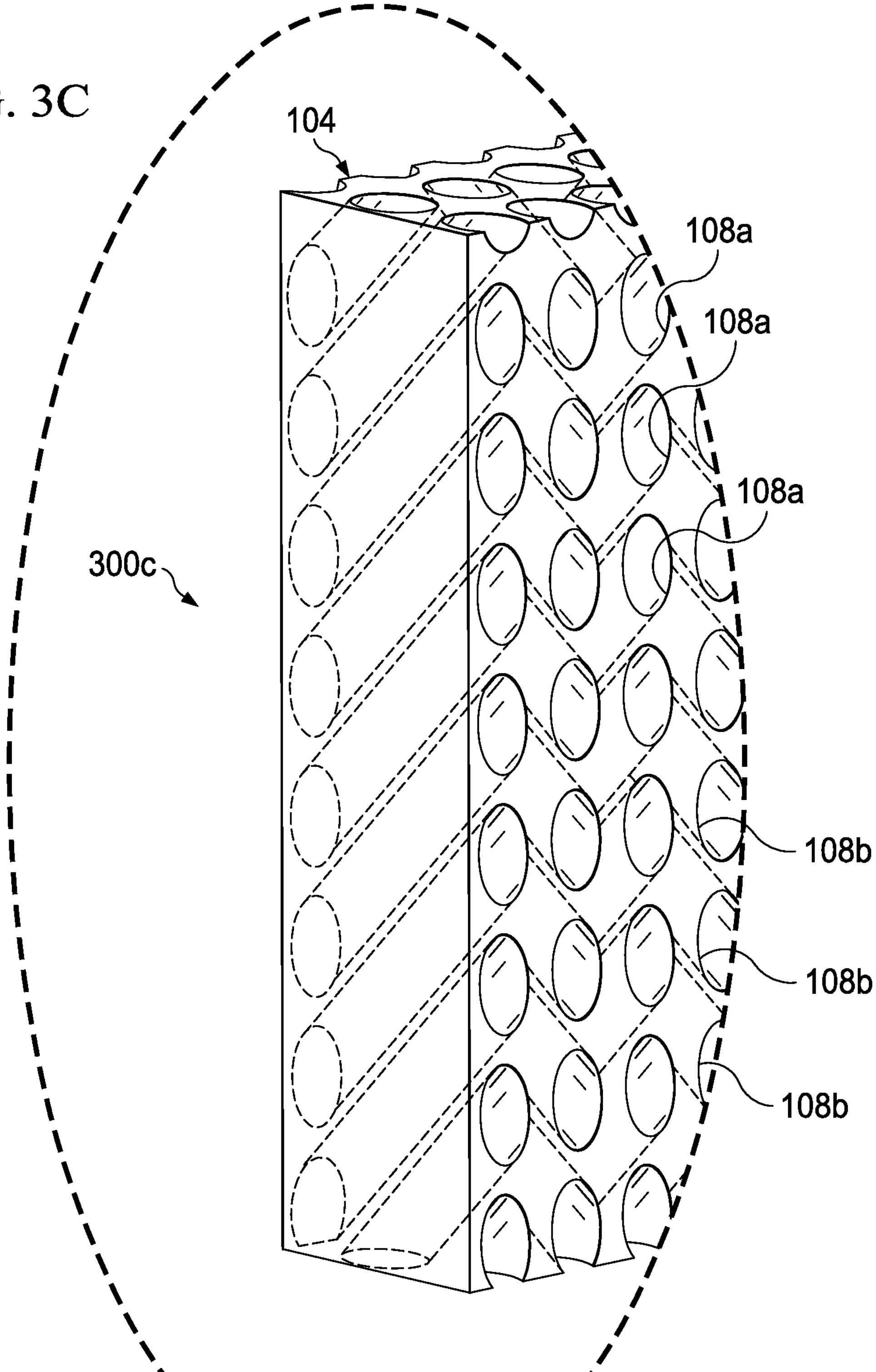

FIG. 3D

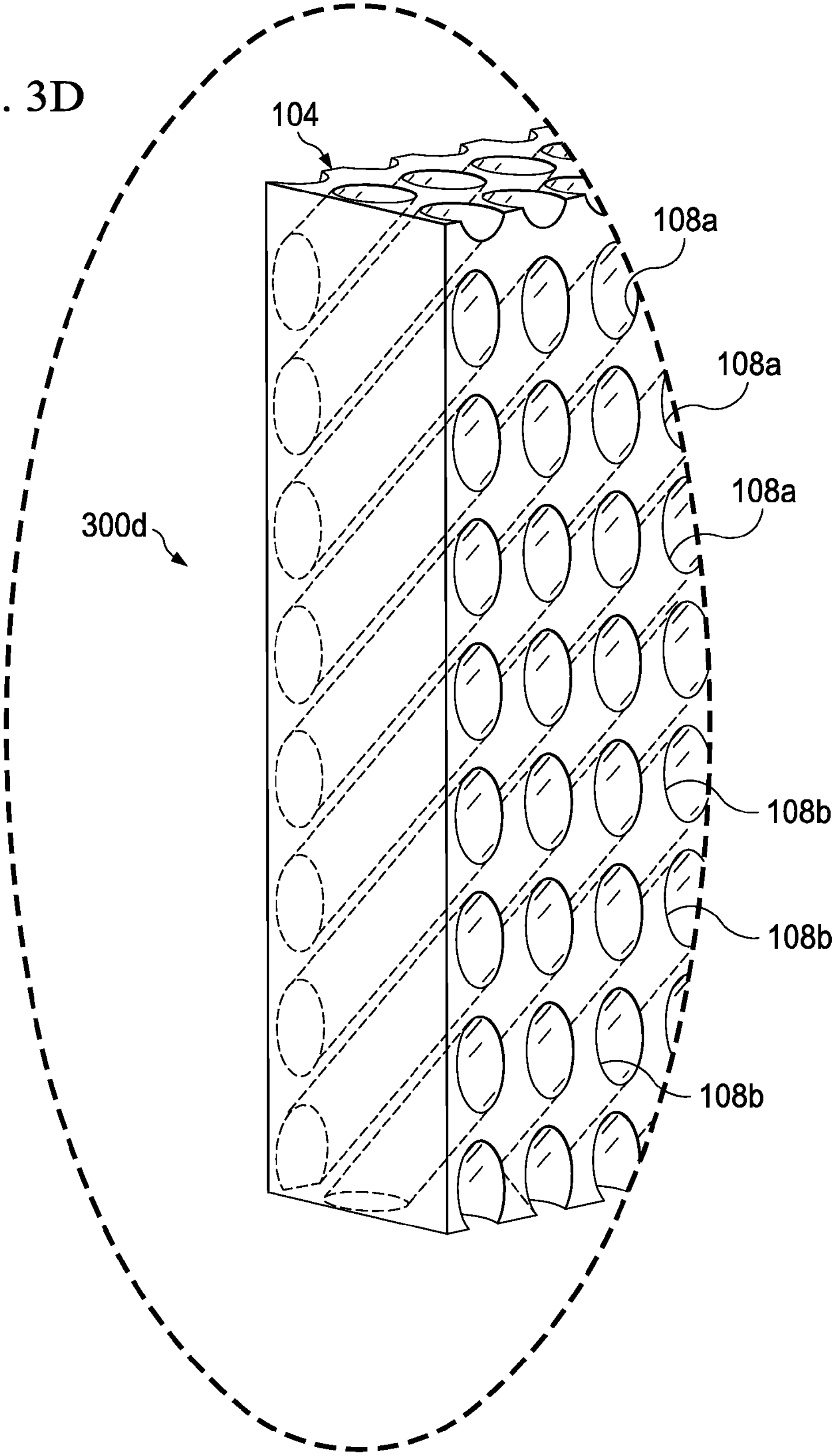

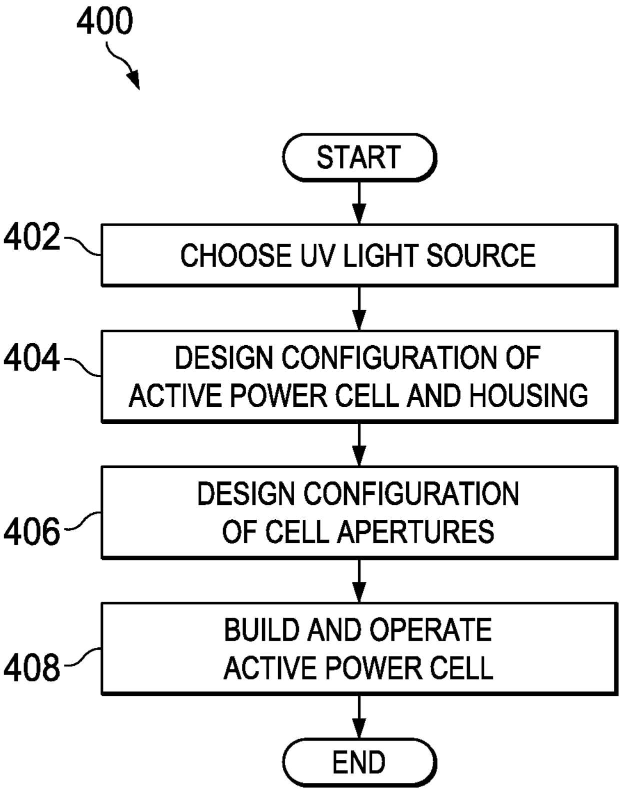
400
START
402
CHOOSE UV LIGHT SOURCE
404
DESIGN CONFIGURATION OF ACTIVE POWER CELL AND HOUSING
406
DESIGN CONFIGURATION OF CELL APERTURES
408
BUILD AND OPERATE ACTIVE POWER CELL
END

FIG. 4

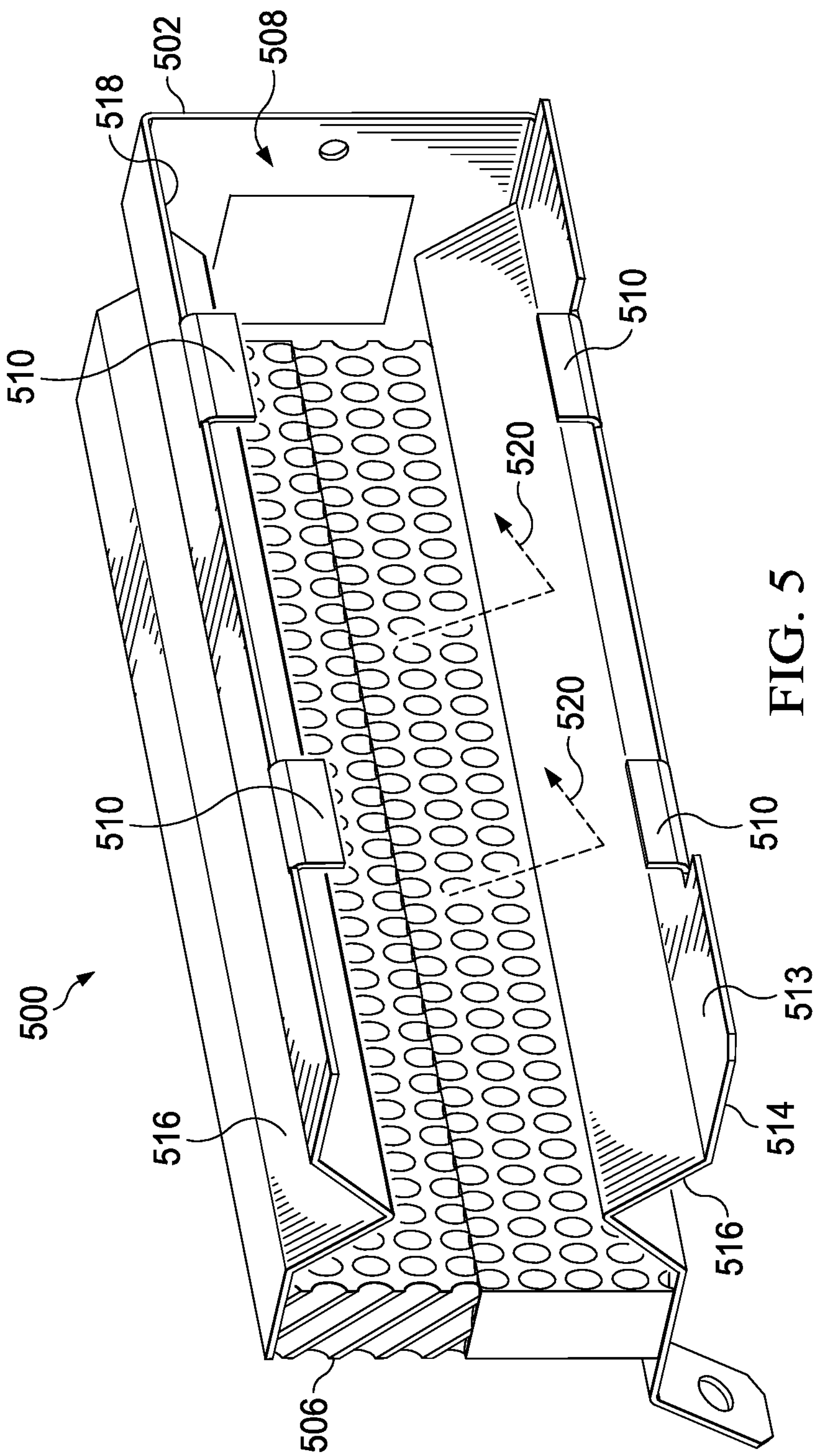

FIG. 5

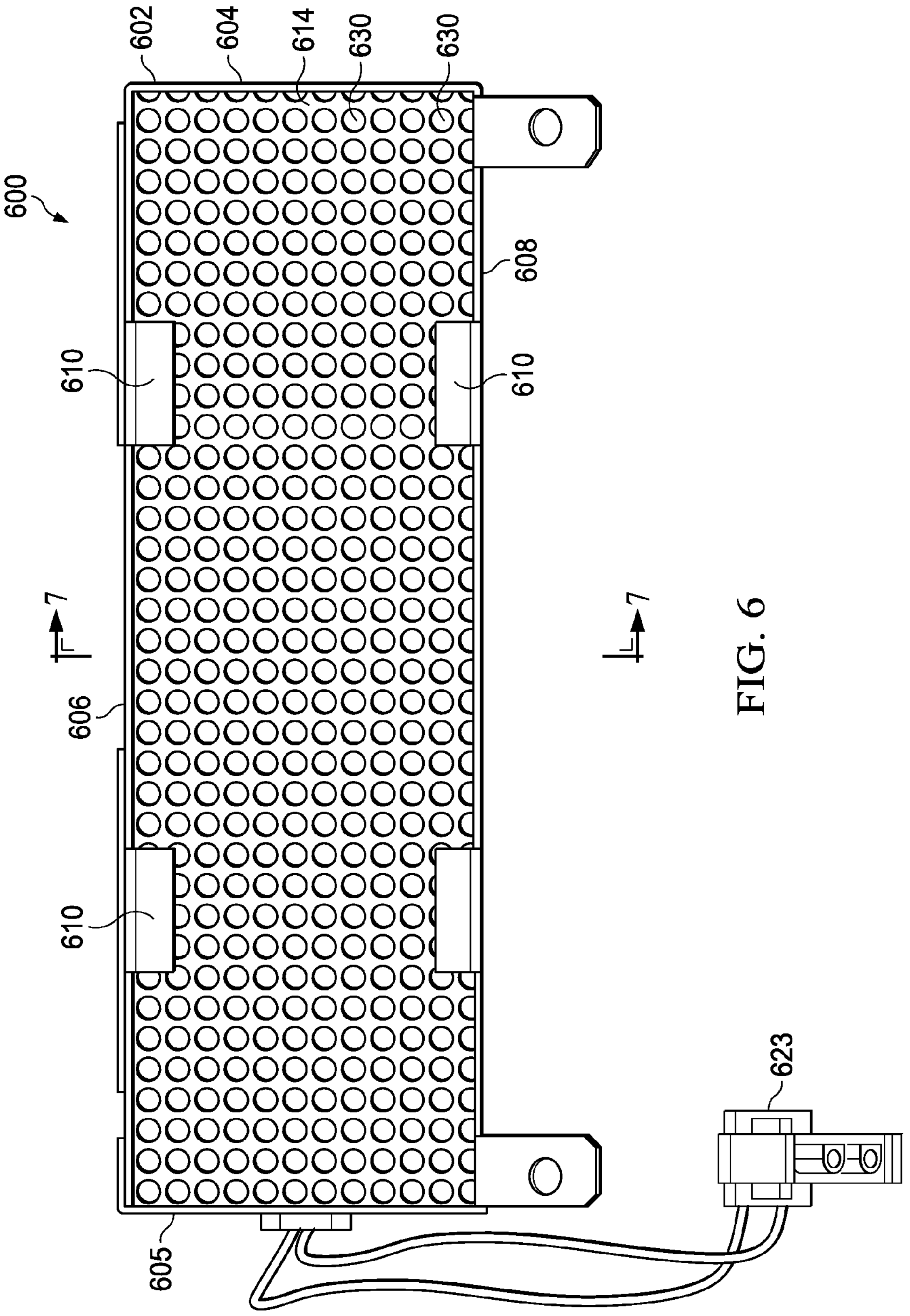

FIG. 6

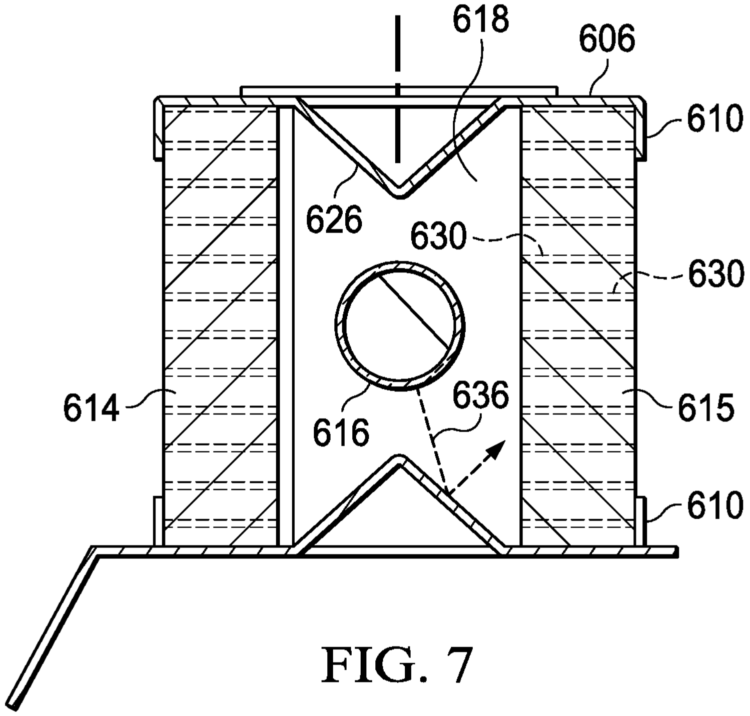

FIG. 7

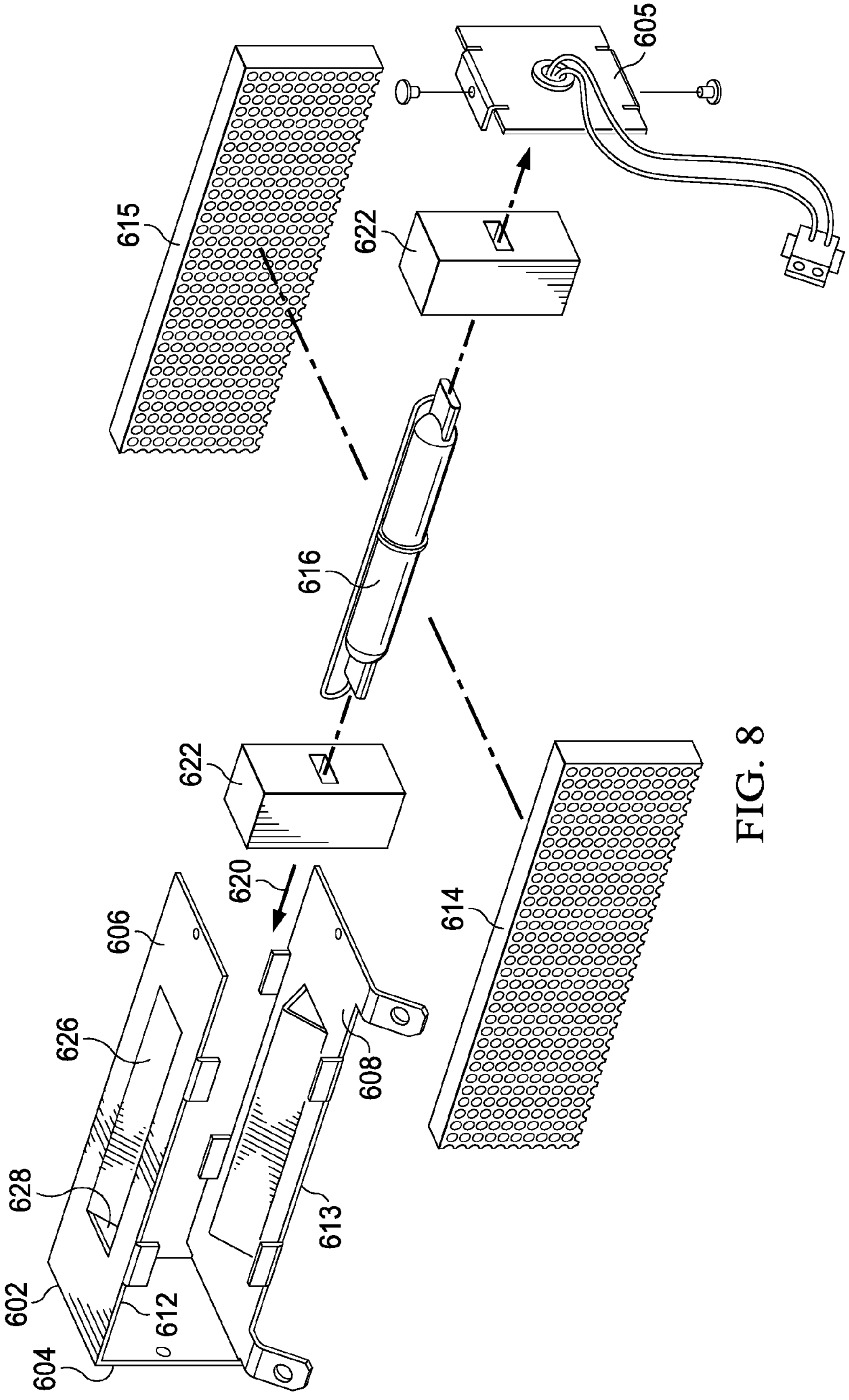

FIG. 8

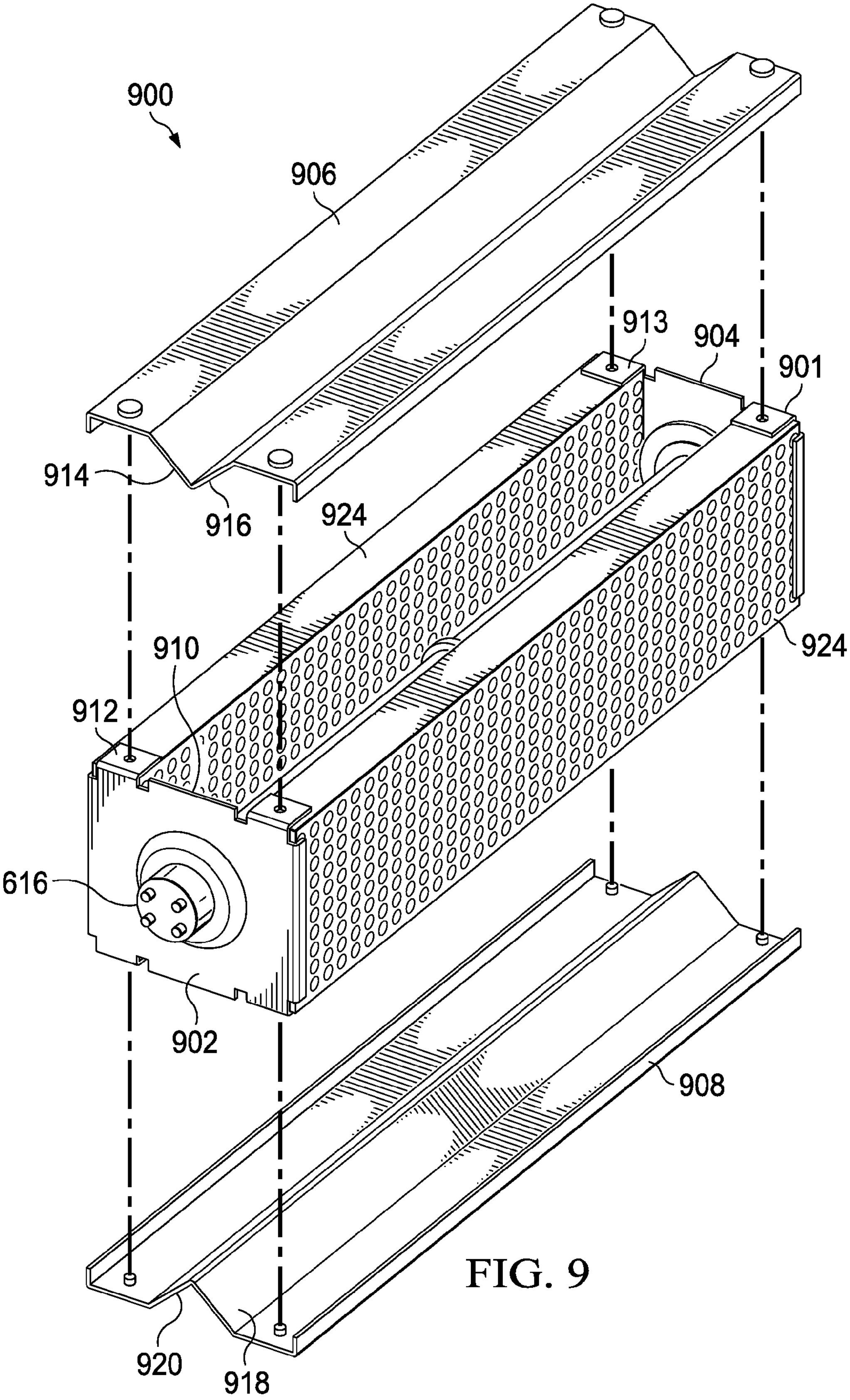

FIG. 9

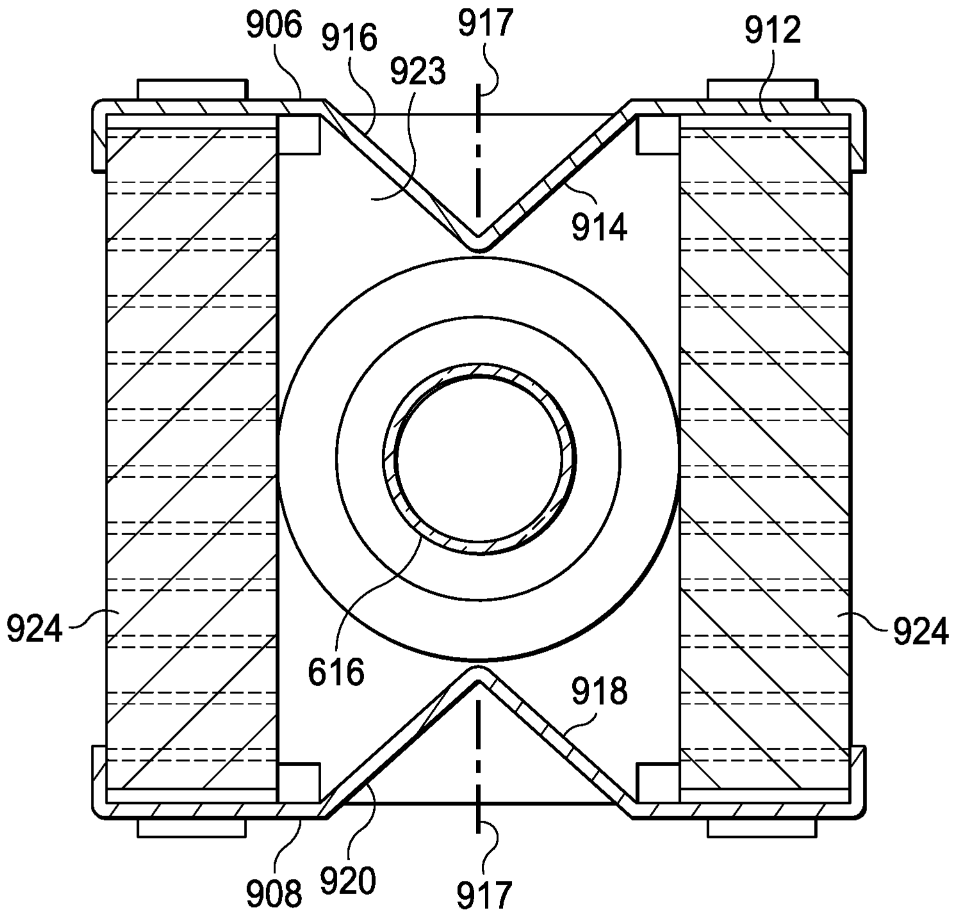

FIG. 10

ACTIVE PHOTOCATALYTIC OXIDATION

CROSS-REFERENCE TO RELATED APPLICATIONS

This application is a Division of U.S. patent application Ser. No. 14/705,046, filed on May 6, 2015, entitled ACTIVE PHOTOCATALYTIC OXIDATION, which published on Aug. 30, 2015, as U.S. Application Publication No. 2015-0231298. U.S. application Ser. No. 14/705,046 is a continuation-in-part of U.S. patent application Ser. No. 13/602,102, filed on Sep. 1, 2012, entitled ACTIVE PHOTOCATALYTIC OXIDATION. U.S. application Ser. Nos. 14/705,046 and 13/602,102, and U.S. Application Publication No. 2015-0231298 are incorporated by reference herein in their entirety.

TECHNICAL FIELD

The present disclosure relates generally to air filtering or purifying systems and, in particular, to systems and methods of using ultraviolet light to oxidize and purify the ambient environment using photocatalytic oxidation.

BACKGROUND

Conventional systems, such as high-efficiency particulate air (HEPA) filtration systems, require contaminates to come in direct contact with a filter to remove such contaminants and fail to address any surface contaminants. Other conventional systems using ultraviolet light to oxidize ambient air typically use particle filters that, when energized by a centrally located ultraviolet (UV) light source, aid in purifying the ambient air and environment by converting toxic compounds to benign constituents. Such systems typically include rows of coated particle filters that form a structure to selectively envelope the UV light source. The UV light in such systems fails to adequately expose the surfaces of conventional particle filters to the UV light and, thus, such systems typically yield rates of photocatalytic oxidation and air filtration that are relatively low.

What is needed, is an effective and efficient system of increasing the rate of photocatalytic oxidation to ultimately increase the rate and efficiency of oxidizing and purifying the ambient environment.

SUMMARY

Embodiments of the present disclosure generally provide systems and methods of using ultraviolet light and photocatalytic oxidation to oxidize and purify the ambient environment.

In one embodiment a photocatalytic oxidation system is provided that comprises a housing. The housing comprising a proximate end panel and a distal end panel. The housing has a longitudinal direction extending from the proximate end panel to the distal end panel. The housing further includes an upper panel extending between top sides of both the proximate end panel and the distal end panel. There is a lower panel extending between lower sides of both the proximate end panel and the distal end panel. The lower panel is spaced from the upper panel. A first active cell panel extends between a first side of the proximate end panel, a first side of the distal end panel, a first side of the upper panel, and a first side of the lower panel. The active first cell

comprises a first plurality of apertures disposed in a transverse manner extending from an inner side to an outer side of the first active cell panel.

Additionally, a second active cell panel extends between a second side of the proximate end panel, a second side of the distal end panel, a second side of the upper panel, and a second side of the lower end panel. The second active cell comprises a second plurality of apertures disposed in a transverse manner extending from an inner side to an outer side of the second active cell panel.

There is an interior chamber bounded by the proximate end and distal end panels, the upper and lower panels, and the first and second active cell panels. An elongate UV bulb is positioned inside the interior chamber and has a center axis that is parallel with the longitudinal direction.

The embodiment further includes a first reflective feature that protrudes inward into the interior chamber from an inner surface of the upper panel. The first reflective feature further extends in the longitudinal direction on the inner surface of the upper panel and is configured to reflect ultraviolet (UV) radiation emitted radially from the elongate UV bulb toward the first plurality of apertures and the inner side of the first active cell, as well as toward the second plurality of apertures and the inner side of the second active cell.

Embodiments may include a first reflective feature that comprises a convex protrusion into the interior chamber from the inner surface of the upper panel.

The first reflective feature may be a V-shape in cross section. And, may additionally be configured to provide stiffening and structural support to the housing.

In various embodiments the inner side and/or the surfaces of the first plurality of apertures of the first active cell panel are coated with a photocatalytic material.

In various embodiments the first reflective feature is provided over a portion of an entire longitudinal length of the upper panel.

Additionally, in some embodiments a center longitudinal axis of the first reflective feature is aligned with the center axis of the elongate UV bulb.

In various embodiments, the reflective feature comprises a metal UV reflective surface.

Additionally, some embodiments further comprise a second reflective feature that protrudes inward into the interior chamber from an inner surface of the lower panel. The second reflective feature is provided in the longitudinal direction on the inner surface of the lower panel and is configured to reflect UV radiation, which is emitted radially from the elongate UV bulb toward the lower panel, toward the first plurality of apertures and the inner side of the first active cell, as well as toward the second plurality of apertures and the inner side of the second active cell.

Another embodiment of a photocatalytic oxidation system comprises an inner cavity; an elongate UV bulb having a central axis in a longitudinal direction and positioned in the inner cavity; and first and second active cell panels positioned on opposing sides of the inner cavity and each being parallel with the central axis of the elongate UV bulb. The first and second active cell panels each comprise an inner surface that faces the inner cavity. Each of the inner surfaces of the first and second active cell panels are coated with a photocatalytic material configured to exhibit a photocatalytic oxidative process when subjected to UV radiation emitted from the elongate UV bulb. There is also an upper side panel that extends between upper edges of the first and second active cell panels. The upper side panel comprises an upper reflective feature that includes an upper convex protrusion that protrudes from an inner surface of the upper side

panel inward into the inner cavity. The upper reflective feature extends longitudinally on the inner surface of the upper side panel and has a cross section that is a mirror image about a central longitudinal and vertical plane through the upper convex protrusion. The surface of the reflective feature is configured to reflect UV radiation emitted from the elongate UV bulb in the direction of the upper side panel toward both the inner surfaces of the first and second active cell panels. Additionally, a bottom side panel extends between lower edges of the first and second active cell panels.

In various embodiments the first and second active cell panels each further comprise a plurality of apertures disposed in a transverse manner from the inner surface to the outer surface of the first and second active cell panels, and the aperture surfaces are each coated with the photocatalytic material and configured to allow airflow there through.

In other embodiments the bottom side panel may further comprise a lower reflective feature that includes an upper convex protrusion that protrudes from an inner surface of the lower side panel inward into the inner cavity, the lower reflective feature extends in a longitudinal direction on the inner surface of the lower side panel and has a cross section that is a mirror image about a central longitudinal vertical plane through the lower convex protrusion. The surface of the reflective feature is configured to reflect UV radiation emitted from the elongate UV bulb in the direction of the lower side panel toward both the inner surfaces of the first and second active cell panels.

In some embodiments, the cross section of the upper convex protrusion is a V-shape.

In various embodiments, the plurality of apertures transverse from the inner surface to the outer surface of the first and second active cell panels in a diagonal manner.

In some embodiments, the upper reflective feature extends the entire longitudinal length of the upper side panel.

Other technical features may be readily apparent to one skilled in the art from the following figures and descriptions.

BRIEF DESCRIPTION OF THE DRAWINGS

For a more complete understanding of this disclosure and its features, reference is now made to the following description, taken in conjunction with the accompanying drawings, in which:

FIGS. **1**A and **1**B are perspective views of an active photocatalytic oxidation system having an active cell in accordance with an embodiment of the present disclosure;

FIG. **2** is an exploded view of the system shown in FIGS. **1**A and **1**B according to an embodiment of the present disclosure;

FIGS. **3**A-**3**D are partial cross sectional views of the system embodiment shown in FIGS. **1**A, **1**B and **2**;

FIG. **4** is a flow diagram generally illustrating a method of using the system embodiment shown in FIGS. **1**A and **1**B;

FIG. **5** is an partial exploded view of another embodiment of the active photocatalytic oxidation system

FIG. **6** is a side view of another active photocatalytic oxidation system in accordance with an embodiment of the present disclosure;

FIG. **7** is a cross sectional view of FIG. **6** along cross section line A-A;

FIG. **8** is an exploded view of the active photocatalytic oxidation system of FIG. **6**;

FIG. **9** is an exploded view of another embodiment of an active photocatalytic oxidation system; and

FIG. **10** is a cross section view of the assembled embodiment of FIG. **9** taken perpendicular to a longitudinal axis of the embodiment.

DETAILED DESCRIPTION

The present disclosure relates generally to air purifications systems methods and methods, in particular, to systems and of using ultraviolet light and photocatalytic oxidation to oxidize and purify the ambient environment. Embodiments of air purification systems in accordance with the invention will now be described more fully hereinafter with reference to the accompanying drawings, in which preferred embodiments of the invention are shown. This invention may, however, be embodied in many different forms and should not be construed as limited to the embodiments set forth herein; rather, these embodiments are provided so that this disclosure will be thorough and complete, and will fully convey pertinent aspects of the invention to those skilled in the art.

An embodiment of a photocatalytic oxidation system could include an active cell having multiple rows of selectively arranged specially treated and coated cell apertures that, when energized by light emitted from a centrally located ultraviolet (UV) light source, aid in oxidizing and purifying the ambient air, surrounding active cell surfaces or ambient environment by converting toxic compounds to benign constituents and controlling or neutralizing contaminants in the ambient environment. The multiple rows of cell apertures could form a honeycomb-like, array or other suitable structures selectively positioned to envelope or at least partially surround the UV light source. By selectively positioning the cell apertures to maximize UV exposure, the active cell increases the relative rates of photocatalytic oxidation and purification when compared to conventional oxidation systems, purification systems, or particle filters. Additionally, by including additional strategically placed angled reflective surfaces within a structure surrounding the UV light source, additional UV light emitted from the UV light source can be reflected from the interior reflective surfaces towards the active cells surfaces to further increase the efficiency of the photocatalytic oxidation and purification of, for example, air, being moved through the photocatalytic oxidation system.

FIGS. **1**A and **1**B are perspective views of an embodiment of a photocatalytic oxidation system **100**. Here, the photocatalytic oxidation system **100** could include housing **102**, active cells **104**, medians **106**, cell apertures **108**, and lighting assembly **110** as generally shown in FIGS. **1**A and **1**B and described in further detail herein. It should be understood that system **100** and active cell **104** could also include, for example, any suitable purification particle filtering system, system, oxidation active cell system, photocatalytic system, neutralizing systems, air filtration system, or combination thereof. It should also be understood that system **100** and active cell **104** shown in FIGS. **1**A and **1**B are for illustrative purposes only and that other suitable systems or subsystems could be used in conjunction with or in lieu of system **100** or active cell **104** and their various embodiments.

Housing **102** could include any suitably sized, shaped or configured frame, frame-like structure, housing, or housing-like structure to aid in maintaining a particular configuration of two or more active cells **104** relative to each other. The housing **102** could be coupled to or include a proximate end **102***a*, a distal end **102***b*, and side ends having lips **102***c*, **102***d*, **102***e* and **102***f* (note that lips **102***e* and **102***f* are hidden

in the views shown). In various embodiments, the housing **102** could be about 5 to 20 inches in length from the proximate end **102**a to the distal end **102**b.

The proximate end **102**a, distal end **102**b, and side edges, which have elongated retaining lips **102** c, **102** d, **102** e and **102** f are collectively referred to herein as housing **102**. It should be understood that housing **102** could be constructed of any suitable material such as a metallic material, plastic material, a polymer, or any suitable combination thereof and include any number of suitable labels, constructs, attachments, binding materials, and accessory like elements. It should also be understood that housing **102** or parts of housing **102** may be constructed or assembled in any suitable manner including, for example, by tabs, screws, rivets, bolts, connectors, tight fits, tapes, adhesives, magnets, sleeves, other securing or retaining mechanisms, or any combination thereof.

In various embodiments, lips **102**c, **102**d, **102**e and **102**f aid in retaining the active cells **104**a, **104**b, **104**c, and **104**d (collectively, referred to herein as active cells **104**) in a particular fashion relative to each other and within the system **100**. Active cells **104** could include any shaped or configured frame, structure, frame-like structure, housing, housing-like structure, or any combination thereof. Active cells **104** could include a first side exposed to an ambient environment outside of the photocatalytic oxidation system and a second side exposed to the interior chamber of the photocatalytic oxidation system **100**.

Active cells **104**a and **104**b shown in FIG. **1**A could be included as a unitary structure or as two or more separate structures. Similarly, active cells **104**c and **104**d shown in FIG. **1**B could be included as a unitary structure or as two or more separate structures. In one embodiment, active cells **104** could be about 5 to about 20 inches in length. In one embodiment, active cells **104**a and **104**b could be disposed adjacent to one another along the median **106**a as shown in FIGS. **1**A and **1**B. Likewise, active cells **104**c and **104**d (which are hidden in FIG. **1**A) could be disposed adjacent to one another along a median **106**b (also hidden in FIG. **1**A. Medians **106**a and **106**b can be collectively referred to herein as medians **106**.

In various embodiments, each of the active cells **104** could include any suitable number, size, shape, or configuration of pass-through structures or apertures such as, for example, cell apertures **108**a, **108**b, **108**c, and **108**d as generally shown in FIGS. **1**A and **1**B. Cell apertures **108**a, **108**b, **108**c and **108**d (and any other cell apertures included in the active cells **104**) are collectively referred to herein as cell apertures **108**. Cell apertures **108** could include any suitably sized, shaped or configured structure to allow ambient airflow from the outside of system **100** to pass through to an internal area of system **100** and vice versa according to one embodiment of the present disclosure.

In various embodiments, cell apertures **108** could be arranged in multiple rows, in a somewhat honeycomb-like structure or array of apertures or tube-like structures. Each of the cell apertures **108** could be disposed in a transverse or diagonal fashion relative to the housing **102** or medians **106**, rather than disposed in a relatively parallel or perpendicular fashion relative to housing **102** or medians **106**. As an example, each of cell apertures **108** could be transversely disposed about 45 degrees (plus or minus 20 degrees) relative to medians **106** according to one embodiment of the present disclosure.

In various embodiments, the cell apertures **108**a in the active cell **104**a could be disposed about +45 degrees (plus or minus 20 degrees) relative to an x-axis of median **106**a, while the cell apertures **108**b in the active cell **104**b could be disposed about −45 degrees (plus or minus 20 degrees) relative to the same x-axis of median **104**a. The, cell apertures **108**c in active cell **104**c could be disposed at about +45 degrees (plus or minus 20 degrees) relative to an x-axis of median **106**b, while the cell apertures **108**d in active cell **104**d could be disposed about −45 degrees (plus or minus 20 degrees) relative to the same x-axis of median **106**b.

In other embodiments, the cell apertures **108** could be positioned between about plus or minus 20 degrees and 75 degrees relative to medians **106**. In still other embodiments, the optimal disposition of cell apertures **108** could be about plus or minus 45 degrees relative to medians **106**. One reason for transversely or diagonally disposing the cell apertures **108** is to maximize the amount of UV light emitted from a centrally located UV light source that will impinge on the surfaces of the active cells **104** and the apertures **108** integrated in the active cells.

FIG. **1**A also depicts a lighting assembly **110** that is connected to a connector **206** at one end and adapted to receive power to energize the UV lighting assembly positioned inside the housing as shown in FIG. **2**.

The active cells **104**, cell apertures **108**, or any combination thereof could be uniformly or selectively coated or treated with one or more photocatalytic materials, such as titanium dioxide and similar compounds. The active cells **104** that are treated with such photocatalytic materials and energized by receiving UV light emitted from a centrally located UV light source, operate by supporting a photocatalytic oxidation process that aides in the purification of the ambient air about the active cells by converting toxic compounds, via oxidation, to benign constituents. In one embodiment, active cells **104**, cell apertures **108**, or any suitable combination thereof may be coated with a suitable hydrophilic photocatalytic coating having non-nano titanium dioxide with several transition elements added to the coating to enhance or help optimize the overall photocatalytic effect.

FIG. **2** is an exploded view of assembly **200** of an embodiment of system **100**. It should be understood that assembly **200** shown in FIG. **2** is for illustrative purposes only and that other suitable views, systems or subsystems could be used in conjunction with **100**. Additionally, only a single active cell **104** wall (**104**a, **104**b) is shown and a second active cell **104** wall (**104**c, **104**d) is left out of the figure to make the interior of the assembly **200** easier to view.

In various embodiments, assembly **200** shown in FIG. **2** generally illustrates an embodiment of an unassembled portion of system **100** shown in FIGS. **1**A and **1**B. Here additional subassemblies and parts of the embodiment are shown to include a UV light source **202**, and a spacer **204**. As illustrated by FIGS. **1**A and **2**, when system **100** is fully assembled UV light source **202** is disposed between active cells **104**a, **104**b, **104**c, and **104**d according to one embodiment of the present disclosure such that the when the UV light source **202** emits UV light, the UV light impinges on the active cell surfaces facing the interior of the housing. Additionally, the UV light impinges on the interior surfaces of the lower and upper housing panel surfaces **111**, **112**. In various embodiments the lower and upper housing panel surfaces **111**, **112** are reflective surfaces that reflect UV light emitted from the UV light source **202** back toward the UV light and toward the inner surfaces of the active cell **104**a and the plurality of interior surfaces of the cell apertures **108**a, **108**b. In order to be reflective surfaces, the inner surfaces of the housing panels **111**, **112** can have their

surfaces buffed, have a reflective coating placed thereon, or be made of a material, such as aluminum, stainless steel, certain types of plastic/polymers or other materials that reflect UV light. The reflective surfaces of the inner surfaces of the lower and upper housing panels are provided to reflect UV light emitted from the UV light source **202** onto and into the aperture surfaces of the apertures **108** thereby enhancing the efficiency of the photocatalytic oxidation reaction and air cleaning capability of the system **100**.

In various embodiments, the UV light source **202** could include any suitably sized, shaped, or configured UV light source, UV lamp, UV bulb, UV light emitting diode array, other suitable sources of UV or UV subtype C (UVC) radiation, or any combination thereof providing a suitable amount of UV intensity to activate the coating on the active cells **104**. The UV light source **202** is configured to provide UV light of sufficient intensity to induce photocatalytic oxidation of coating or the treatment applied to the surface of the active cells **104**. In various embodiments the UV light source **202** includes, for example, a UV light source having a wavelength of 185 nanometers (nm) or 254 or a broad spectrum lamp capable of providing UV light providing both wavelengths of 185 nm and 254 nm to induce photocatalytic oxidation of active cells **104**.

When system **200** is assembled, the spacer **204**, which is part of the lighting assembly **110**, aids in maintaining the relative central-axial position of UV light source **202** within housing **102**. Although a particular configuration of spacer **204** is shown in FIG. **2**, it should be understood that spacer **204** could include any suitable size, shape, or configuration to maintain the relative position of the UV light source inside the housing with respect to the active cells **104**. In various embodiments, spacer **204** could also be used to maintain the relative position of the lighting assembly **110** relative to housing **102** and connector **206** as generally shown in FIG. **2**.

The connector **206** can be part of the lighting assembly **110** and is electrically coupled with UV light source **202**. Connector **206** can include any suitable connection to a power source (not shown) for powering the lighting assembly **110**. Connector **206** could include any suitably sized, shaped, or configured connector including, for example, any suitable multiple pin connector with two or more electrical contacts.

As illustrated by FIG. **2**, when assembly **200** is fully assembled, the UV light source **202** is disposed centrally within the housing chamber and between the active cells **104***a*, **104***b*, **104***c*, and **104***d*. Although active cells **104** are disposed opposite from one another relative to UV light source **202**, it should be understood that the relative positions and configurations of active cells **104** could be varied in other suitable manners. The UV light source is configured to emit light radially outward from the surface of the UV light source such that the emitted UV light impinges on the exposed surfaces of the active cells **104** that are facing the UV light source.

For example, the active cells **104** could be disposed in three sides or as three walls of the housing **102** and about the chamber within. In another example, the active cells **104** could be disposed in a triangular manner encasing or substantially surrounding UV light source **202**. In another example, the active cells **104** could be disposed in a box-like manner encasing or substantially surrounding the entire perimeter of the chamber within the housing and about the UV light source **202** according to another embodiment. As still another example, active cells **104** could be disposed in a circular manner encasing or substantially surrounding UV light source **202** in another embodiment.

FIGS. **3**A, **3**B, **3**C and **3**D are somewhat simplified partial cross sectional views **300***a*, **300***b*, **300***c* and **300***d* (collectively referred to herein as views **300**) of exemplary active cells **104** in accordance with embodiments of the present disclosure. It should be understood that views **300** shown in FIGS. **3**A-**3**D are for illustrative purposes only and that any other suitable view, system or subsystem could be used in conjunction with or in lieu of active cells **104** shown in views **300** according to one embodiment of the present disclosure.

In the embodiment shown in FIG. **3**A, active cells **104***a* and active cells **104***b* are structurally independent from each other. Cell apertures **108***a* in active cell **104***a* could be disposed in a transverse direction about minus Θ degrees relative to an x-axis of median **106***a*, while cell apertures **108***b* in active cell **104***b* could be disposed about positive Θ degrees relative to the same x-axis of median **106***a* as shown in FIG. **3**A. In an exemplary embodiment, active cell **104***a* and active cell **104***b* are structurally independent from each other. The cell apertures **108***a* in active cell **104***a* could be disposed in a transverse direction about minus 45 degrees (plus or minus 20 degrees) relative to an x-axis of median **106***a*, while cell apertures **108***b* in active cell **104***b* could be disposed about positive 45 degrees (plus or minus 20 degrees) relative to the same x-axis of median **106***a* as shown in FIG. **3**A.

In the active cell embodiment **300***b* shown in FIG. **3**B, active cell **104***a* and active cell **104***b* are independent structures of cell aperture arrays. The cell apertures **108***a* and **108***b* are disposed in the same transverse direction from a first side of the active cell to a second side of the active cell and are each disposed about 45 degrees (plus or minus 20 degrees) relative to a reference axis found in active cells **104**.

In the active cell embodiment **300***c* shown in FIG. **3**C, active cells **104***a* and active cells **104***b* are included as a unitary structure. Columns of cell apertures **108***a* in active cell **104***a* are transversely disposed from a first side to a second side of the active cell structure at about minus 45 degrees (plus or minus 20 degrees) relative to a horizontal x-axis along a top edge of the active cell **104**, while alternate columns of cell apertures **108***b* are transversely disposed from a first side to a second side of the active cell structure at about positive 45 degrees (plus or minus 20 degrees) relative to the horizontal x-axis long a tope edge of the active cell **104**.

In the active cell **300***d* embodiment shown in FIG. **3**D, active cell **104** is a unitary structure comprising a matrix of a plurality of cell apertures **108**. Cell apertures **108** are disposed in a transverse direction from a first side of the active cell to a second side of the active cell and are disposed at about 45 degrees (plus or minus 20 degrees) relative a reference plane parallel with a front surface of the active cell **104**.

In one embodiment of the present disclosure, the majority, if not all, of active cells **104** in system **100** are capable of performing a photocatalytic response to being radiated or illuminated by UV light emitted from the UV light source **202**. By optimizing or maximizing the surface area of UV light exposure of the surface of the active cell **104**, including the inner surfaces of the cell apertures **108**, the system **200** maximizes the relative rates and amount of photocatalytic oxidation performed by the photocatalytic materials on the surfaces of the active cells **104**, and thus rates of oxidation and purification of the air in the ambient environment are

greater than those of conventional prior art oxidation systems, purification systems, or particle air purifiers and filters. In one embodiment, photocatalytic oxidation in system **200** could produce one or more of the following oxidizers: Hydroxyl Radicals (OH), Vaporized Hydrogen Peroxide (H_2O_2), Super Oxides (O_2—) or Low Level Ozone (O_3).

In one embodiment, active cell **104** could include any suitably sized and dimensioned cell apertures **108** such as, for example, cell apertures **108** that are about 55 square millimeters (mm^2) in volume. In other embodiments, active cell **104** could include cell apertures **108** where the average or median distance from an edge of one or more of cell apertures **108** to the center of UV light source **202** having, for example, a diameter of about 20 mm, could be about 15 mm.

FIG. **4** is a simplified flow diagram generally illustrating a method **400** of customizing and using active cell **104** shown in FIGS. 1A, **1**B, **2** and **3** in accordance with an embodiment of the present disclosure. Although a particular set of steps are illustrated as method **400**, it should be understood that any number of steps may be included or removed in conjunction with the steps should as method **400** or in lieu of one or more steps of method **400** according to one embodiment of the present disclosure.

In one embodiment, method **400** begins with step **402** that includes choosing a UV light source such as, for example, UV light source **202** shown in FIG. **2**, of suitable size, shape and configuration for a particular photocatalytic oxidation and ambient environment oxidation and purification application.

In step **404**, method **400** includes designing the configuration of an active cell and housing needed to house the UV light source chosen in step **402**. As examples, the active cells could be designed to encase or substantially surround the chosen UV light source on opposing sides such as, for example, the relative positions of UV light source **202** and active cells **104** shown in FIG. **2**. In another design, the system could include designing a system where the active cells are configured in a triangular fashion or circular fashion about the UV light source.

Depending on the active cell configuration chosen, step **406** may include choosing the relative angle of the cell apertures in the active cells. For example, in step **406**, cell apertures **108** shown in FIG. **3**, could be configured to be about 45 degrees (plus or minus 20 degrees) from a reference point such as, for example, median **106** to maximize UV exposure to the respective surfaces of active cell **104** and cell apertures **108**.

Once design specifications in step **406** are complete, method **400** continues with step **408**. Step **408** includes building and operating the active cell to facilitate photocatalytic oxidation and air filtration. For example, by maximizing UV exposure on the surfaces of the active cells **104**, the relative rates of photocatalytic oxidation with the ambient air and thus air filtration are maximized and superior the photocatalytic oxidation rates of previous conventional oxidation system, purification systems, or particle air purifiers and filters.

Accordingly, embodiments of the present disclosure optimize or increase an active photocatalytic oxidation system increasing or maximizing the potential for ultraviolet (UV) light to impinge on the surface and apertures of the active cells, to thereby increase the rates of photocatalytic oxidation and the oxidation and purification of the ambient environment about and flowing through the inner chamber of the housing and individual apertures.

It may be advantageous to set forth definitions of certain words and phrases used in the present disclosure. The terms "ambient" and "environment" and its respective derivatives refer to any surrounding areas, air, gasses, solids, liquids, organisms, or surfaces. The term "couple" and its derivatives refer to any direct or indirect communication between two or more elements, whether or not those elements are in physical contact with one another. The terms "include" and "comprise," as well as derivatives thereof, mean inclusion without limitation. The term "or" is inclusive, meaning and/or. The phrases "associated with" and "associated therewith, "as well as derivatives thereof, may mean to include, be included within, interconnect with, contain, be contained within, connect to or with, couple to or with, be communicable with, cooperate with, interleave, juxtapose, be proximate to, be bound to or with, have, have a property of, or the like.

Referring now to FIG. **5**, a variation of the embodiment shown in FIG. **2** is depicted. A housing **500** is shown. This housing **500** can be used in place of the housing **102** shown in FIG. **2**. Housing **500** has a distal end panel **502** and a proximate end panel (not specifically shown). Two active cell panels **506** are positioned as side walls on opposing walls of the housing **500**. One of the active cell panels **506** is not depicted in FIG. **5** so that the interior chamber of the housing can be viewed. The active cells **506** are held in place by retaining tabs or lips **510** extending from side edges of an upper panel **512** and lower panel **514**.

When the UV light source **202** is positioned inside the interior chamber of the housing, the UV light source may be an elongated UV light source and define a longitudinal axis extending from the proximate end panel to the distal end panel **502** of the housing **500**.

The upper and lower side panels **512**, **514** each may extend from the proximate end panel to the distal end panel **502**. The combination of the proximate end panel, distal end panel **502**, the two active cell panels **506** positioned as side walls on opposing sides of the housing **500**, and the upper and lower side panels each provide interior surfaces that define the interior chamber **508** of the housing **500**.

On at least one of the upper and/or lower side panels **512**, **514**, there is a reflecting feature **516** that is configured to increase the amount of UV light directed from the UV light source **202** to the inner and cell aperture surfaces of the active cell panels **506**. In some embodiments, the reflecting feature **516** comprises an inwardly convex longitudinal protrusion that extends from an inner surface portion **513** of the, for example, lower side panel **514** inward into the interior chamber **508**. The convex longitudinal protrusion is elongated in the longitudinal direction of the upper side panel **512**. In the embodiment shown in FIG. **5** the reflecting feature **516** extends the entire longitudinal length of the upper side panel **512**. The inner surface **518** of the upper side panel **512** is reflective to UV light. In some embodiments, at least the inner surface of the reflective feature **516** is very reflective to UV light or the UV spectrum. The reflectivity of the inner surface of the upper side panel **512** may be due to the surface being buffed smooth or coated to have a reflective surface. In other embodiments, the reflectivity is due to the surface being a bare or reflective metal surface.

It should be understood that the lower side panel **514** may also include a reflective feature **516** that is similarly configured as the reflective feature **516** that is part of the upper side panel **512**.

The convex longitudinal protrusion may appear like an elongated concave trough on the outside surface of the upper side panel **512**. One functional purpose of the reflective

feature **516** is to reflect and redirect UV light emitted from the UV light source **202** onto the upper or lower side panels **512**, **514** toward the inner surface of the active cell panels **506** and the interior surfaces of the cell apertures in order to increase the photocatalytic oxidation reaction on the surfaces of the active cell panels **506** and thereby increase the efficiency and efficacy of the air, gas or liquid purification process performed by embodiments. The reflection or redirection of UV light on the surface of the reflecting feature **516** is shown by the arrows **520** indicating UV light emitted from a UV light source (not specifically shown in this figure) and being reflected and redirected toward the inner coated surfaces of the active cell panels **506**. Another functional purpose of the reflective feature **516** is for the longitudinal protrusion on the upper and/or lower side panels **512**, **514** to provide additional stiffening and structural support for the overall housing structure **500**.

Referring now to FIGS. **6**, **7** and **8** another embodiment of an active photocatalytic oxidation system **600** is disclosed. FIG. **6** is a side view of the active photocatalytic oxidation system **600**. The housing **602**, in this embodiment, has a distal end **604** that attaches to an upper housing portion **606** and a lower housing portion **608**. The upper and lower housing portions **606**, **608** extend perpendicularly from the distal end **604**. The upper and lower housing portions **606**, **680** are basically parallel to each other and to some degree a mirrored image of each other. The housing **602** can also be easily viewed in FIGS. **8** and **7**. Along each side hedge **612** of the upper housing portion **606** are one or more retaining tabs or clips **610** that extend downwardly from the upper housing portion **606** and configured to support, position and hold a first and second active cell panel **614**, **615** in place on a first side and second side of the housing **602**. Similarly, along each side and **613** of the lower housing portions **608** there are one or more retaining tabs or clips **611** extend upwardly from the lower housing portions **608** and are configured to support, position hold the first and second active cell panel **614**, **615** in place on the first side and second side of the housing **602**.

FIG. **7** is a cross-sectional view of FIG. **6** at the cross section line A-A looking from the distal end **604** toward the proximate end **605** of the housing structure **602**. Additionally, FIG. **8** is an exploded view of the active photocatalytic oxidation system **600**. An elongate UV bulb **616** is positioned within a cavity **618** within the housing structure **602**. The cavity **616** is defined by the inner surfaces of the upper and lower housing portions **606**, **608**, the first and second active cell panels **614**, **615**, and the proximate and distal ends **605**, **604** of the housing structure **602**. The elongate UV bulb **616** defines a longitudinal direction or axis **620** extending from the proximate end **605** to the distal end **604**. At either end of the UV bulb **616** are support structures **622** that support the UV bulb **616**. One or both of the support structures **622** have a socket or connector **624** for electrically connecting and providing power to the UV bulb **616**. Although not specifically shown, there may also be an electronic circuit within one or both of the support structures **622**. The electronic circuit may include a ballast circuit for a florescent UV bulb, circuitry to drive high-voltage to the UV bulb, or electronics that supports an array of ultra bright UV diodes in a ultraviolet UV bulb configuration. Electrical wires and a connector **623** may be provided to connect the UV bulb **616** to a power source. The

Additionally, the upper housing portion **606** comprises a reflective feature **626**, that when viewed from the cavity **618**, extends in the longitudinal direction as an inwardly convex longitudinal trough. The inner surface of the inwardly convex longitudinal trough (ICLT) or reflective feature **626**, is reflective or highly reflective in the UV spectrum. In some embodiments the reflective feature **626** comprises the ICLT. The reflective feature **626** may also include a UV reflective coating, buffed surface, or other additional treatment to the inner surface of the reflective feature **626**. As can be easily seen in FIGS. **7** and **8**, the upper housing portions **606**, when viewed from the outside of the housing structure **602**, has an elongated trough or indentation that extends in the longitudinal direction **620**. The ICLT **626** in provides additional structural integrity and rigidity to the housing structure **602**. In the embodiment shown the most inward portion of the reflective feature **626** is disposed in a plane or is centered with a longitudinal axis of the elongate UV bulb **616**. In various embodiments the ICLT reflective feature **626** appears like a trough on the outside of the upper housing portion **606** and appears like an elongated protrusion into the cavity **618** on the inside of the upper housing portion **606**. In this embodiment the reflective feature does not extend the full longitudinal distance between the proximate end **605** and distal end **604**. Instead, a gap or airway **628** is established at either end of the reflective feature **626**, which allows for additional airflow between the outside environment of the housing structure **602** end the inner cavity **618**.

When the UV bulb **616** is on and emitting ultraviolet light, the inside surfaces of the reflective feature **626** reflects ultraviolet energy emitted from the UV bulb **616** that is directed toward the inner surfaces of the upper housing portion **606** directly toward the surfaces (i.e., inner surfaces) of the active cell panels **614**, **615** and the respective inner surfaces of the cell apertures **630** that make up the active cell panels **614**, **615** (for example as shown by the arrow **632** in FIG. **7**. It is been found that providing the reflective feature **626** in the upper housing portion **606**, the efficiency and effectiveness of an active photocatalytic oxidation system **600** is increased over a system that does not include the reflective feature **626**.

In various embodiments, the reflective feature **626** is also incorporated or included in the lower housing portion **608** in a manner similar to the description above with respect to the upper housing portion **606** and as shown in FIGS. **6**, **7** and **8**.

In other embodiments the reflective feature **626**, when viewed in cross-section such as the cross-sectional view A-A perpendicular to the longitudinal axis, may have any convex shape, including but not limited to, a V-shape as show, a half circle, half oval, half octagon or other multi-sited or faceted shape that provides a mirrored image of itself about a central longitudinal vertical plane **917** through the trough (shown as dotted lines).

Referring now to FIG. **9** an exploded view of yet another embodiment of an active photocatalytic oxidation system **900** is provided. Additionally, FIG. **10** is a cutaway view perpendicular to the longitudinal direction of the assembled system **900**. In this embodiment, the system **900** has a housing **901** having a proximate end panel **102**, a distal end panel **904**. An upper panel **906** and lower panel **208** extend parallel to each other. The upper panel **906** removably attaches to an upper edge **910** or upper tabs **112** of the proximate end panel **902**. The upper panel **906** extends in a longitudinal direction from the upper edge **910** of the proximate end panel **902** to the upper edge **910** of the distal end panel **904** where it is also removably attached to upper tabs **913**.

The upper panel **906** comprises a reflective feature **914** extending the longitudinal length of the upper panel **906**. The reflective feature **914**, on the inner surface of the upper

panel **906**, includes an inwardly convex longitudinal trough (ICLT) **916** that extends into the interior of the housing **901**. The inner surface of the ICLT reflective feature **914** is reflective or highly reflective to the UV spectrum. In this embodiment, the ICLT reflective feature, from the outside of the housing **901**, looks like a concave trough extending longitudinally from a proximate to a distal end of the upper panel **906**. The ICLT reflective feature **914** looks like a convex longitudinal trough with a reflective surface that extends inwardly into the cavity **923** of the system housing **901**.

The lower panel **908** also comprises a reflective feature **918** that extends the longitudinal length of the lower panel **908**. On the inner surface of the lower panel **908**, the reflective feature **918** includes an inwardly convex longitudinal trough (ICLT) **920** that extends into the interior of the housing **901**. Like the inner surface of the ICLT reflective feature **914**, the ICLT reflective feature **918** is reflective or highly reflective to the ultraviolet spectrum. From the outside of the housing, the ICLT reflective feature **918** looks like a concave trough extending longitudinally from a proximate to a distal end of the lower panel **906**. Furthermore the ICLT reflective feature **918** looks like a convex longitudinal trough with a reflective surface that extends inwardly into the cavity **923** of the system housing from the perspective of inside the system housing **901**. In cross-section, the reflective features **914**, **918** may be a V-shape, half circle, half oval, half hexagon or half any other faceted or multi sided shape that is mirrored about a feature's central longitudinal vertical plane **917** (shown as a dotted line).

The elongate UV bulb **922** is positioned to extend longitudinally between the proximate end panel **902** and the distal end panel **904**. In some embodiments, the proximate end panel **902** and/or distal end panel **904** may each include a centrally located mechanism for holding the UV bulb **922** such that it maintains a central position within the inner cavity **923** of the housing **901**. In various embodiments the upper and lower reflective features **914**, **918** have a longitudinal center line that is parallel with a longitudinal axis of the UV bulb **922**.

On opposing sides of the housing **901** are opposing active cell panels **924** that each extend from opposing sides of the proximate end panel **9022** opposing sides of the distal end panel **904**. The tabs **912** of the proximate end panel **902** and the tabs **914** of the distal end panel **904** are used to attach the upper panel **906**, the two active cell panels **924**, the lower panel **908** and the proximate end panel **902** and distal end panel **904** together so as to establish the inner cavity **923** of the housing **901**.

The high sea LTE reflective features **914**, **918** provide structural stabilizing support for the housing **901** as well as the reflective surface on the inside sides of the upper and lower panels **906**, **908** that increases the amount of ultraviolet light directed toward the inside surfaces of the active cell panels **924** as well as the inside surfaces of the cell apertures comprised within the active cell panels **24**. UV light emitted toward the upper or lower panels is reflected by the reflective features **914**, **918** directly toward the various active surfaces associated with the active cell panels on either side of the housing **901**.

The UV bulb **616** the have a connector at one or both ends of the housing for connecting to electrical power.

During operation, the UV bulb **616** emits UV light radiation radially from the longitudinal axis of the UV bulb. Much of the UV radiation impinges directly upon various inner surfaces of the active side panels. Much of the UV radiation also impinges on the inner surfaces of the upper and lower panels **906**, **908**. The reflective features **914** and **918** on the upper and lower panels increase the amount of UV light intensity that is directed onto the inner surfaces of the active side panels so as to increase the amount or efficiency of the photocatalytic oxidation reaction that occurs on the surfaces of the active panels. Gases or air may flow or be forced to flow through the apertures that make up the active panels such that bacteria, pathogens and other airborne items may be oxidized in the purification process as a result of the reaction occurring between the ultraviolet light and the photocatalytic materials, such as titanium dioxide and other compounds that could hold the surfaces of the active cell panels **924**. By slanting or placing the individual cell apertures at an angle with respect to the horizontal, the surface area inside of the apertures that is impinged by UV light is be increased, especially with the angled reflection of the UV light off of the reflective features **914**, **918**.

While this disclosure has described certain embodiments and generally associated methods, alterations and permutations of these embodiments and methods will be apparent to those skilled in the art. Accordingly, the above description of example embodiments does not define or constrain this disclosure. Other changes, substitutions, and alterations are also possible without departing from the spirit and scope of this disclosure and the following claims.

What is claimed is:

1. A photocatalytic oxidation system comprising:

a housing having a longitudinal central axis;

a UV bulb having a central axis that is parallel with the longitudinal central axis, the UV bulb positioned and configured to emit UV radiation radially about the central axis and into the housing;

a first active cell panel positioned on a first side of the housing and parallel with the longitudinal central axis, the first active cell panel comprising

an inner surface that faces inward toward the longitudinal central axis and incorporates a plurality of elongate apertures having interior surfaces,

wherein the inner surface of the first active cell panel and at least one of the interior surfaces of the plurality of elongate apertures are coated with a photocatalytic material configured to exhibit a photocatalytic oxidative process when subjected to UV radiation emitted from the UV bulb;

a first side panel positioned on a second side of the housing and parallel with the longitudinal central axis, the first side panel comprising

a first reflective feature that includes a convex protrusion that protrudes from an inner surface of the first side panel inward toward the longitudinal central axis, the first reflective feature extending longitudinally on the inner surface of the first side panel for a length that coincides with the first active cell panel but is shorter than the first active cell panel so as to define an airway disposed at an end of the first reflective feature, the airway longitudinally coinciding with at least a portion of the first active cell panel to thereby allow a portion of the air passing through the active cell panel to pass through the airway uninterrupted by the first reflective feature, and

wherein the surface of the first reflective feature is configured to reflect UV radiation emitted from the UV bulb, in the direction of the first side panel, toward the inner surface of the first active cell panel

and at least one of the interior surfaces of the plurality of elongate apertures of the first active cell panel.

2. The photocatalytic oxidation system of claim **1**, further comprising a second active cell panel positioned on a third side of the housing and parallel with the longitudinal central axis, the second active cell panel comprising an inner surface that faces inward toward the longitudinal central axis and incorporates a plurality of elongate apertures, the plurality of elongate apertures including interior surfaces, the inner surface of the second active cell panel and interior surfaces of the plurality of elongate apertures being coated with the photocatalytic material.

3. The photocatalytic oxidation system of claim **2**, wherein the surface of the first reflective feature is further configured to reflect UV radiation emitted from the UV bulb, in the direction of the first side panel, toward the inner surface of the second active cell panel and the interior surfaces of the plurality of elongate apertures of the second active cell panel.

4. The photocatalytic oxidation system of claim **1**, wherein the first reflective feature is further configured to provide structural stiffness to the first side panel.

5. The photocatalytic oxidation system of claim **1**, further comprising a second side panel positioned on a fourth side of the housing and parallel with the longitudinal central axis, the second side panel comprising a second reflective feature that includes a convex protrusion that protrudes from an inner surface of the second side panel inward toward the longitudinal central axis, the second reflective feature extending longitudinally on the inner surface of the second side panel and having a cross section perpendicular to the longitudinal central axis that comprises a mirror image about a second longitudinal plane perpendicular to the inner surface of the second side panel, the second longitudinal plane extending through the longitudinal central axis, wherein the surface of the second reflective feature is configured to reflect UV radiation emitted from the UV bulb, in the direction of the second side panel, toward the inner surface of the first active cell panel and the interior surfaces of the plurality of elongate apertures of the first active cell panel.

6. The photocatalytic oxidation system of claim **1**, wherein the first active cell panel further comprises a median extending longitudinally and dividing the first active cell panel into an upper section and a lower section.

7. The photocatalytic oxidation system of claim **6**, wherein the plurality of apertures disposed on the upper section extend through the first active cell panel at an angle that is different from an angle at which the plurality of apertures disposed on the lower section extend through the first active cell panel.

8. The photocatalytic oxidation system of claim **7**, wherein the plurality of apertures disposed on the upper section extend through the first active cell panel at an angle offset by about 25 to 65 degrees from the median in a first direction, while the plurality of apertures disposed on the lower section extend through the first active cell panel at an angle offset by about 25 to 65 degrees from the median in a second direction opposite the first direction.

9. The photocatalytic oxidation system of claim **1**, wherein the plurality of apertures are arranged into a series of columns positioned along the first active cell panel.

10. The photocatalytic oxidation system of claim **9**, wherein the columns alternate such that apertures in a first column extend through the first active cell panel at a first angle while apertures in a second, adjacent column extend through the first active cell panel at a second angle different from the first angle.

11. The photocatalytic oxidation system of claim **10**, wherein the apertures in the first column extend through the first active cell panel at an angle offset by about 25 to 65 degrees, in a first direction, from an axis perpendicular to an inner surface of the first active cell panel, while the apertures in the second column extend through the first active cell panel at an angle offset by about 25 to 65 degrees, in a second direction opposite the first direction, from the axis perpendicular to an inner surface of the first active cell panel.

12. The photocatalytic oxidation system of claim **1**, wherein the first side panel further comprises an elongate concave trough on an outer surface of the first side panel.

13. A photocatalytic oxidation system comprising:

a housing defining an inner cavity having a longitudinal central axis;

a UV bulb having a central axis that is parallel with the longitudinal central axis, the UV bulb positioned and configured to emit UV radiation radially about the central axis and into the inner cavity;

a first active cell panel positioned on a first side of the housing and parallel with the longitudinal central axis, the first active cell panels comprising

an inner surface that faces the inner cavity and incorporates a plurality of elongate apertures disposed in a transverse manner from the inner surface of the first active cell panel to an outer surface of the first active cell panel, the apertures having interior surfaces,

a median extending longitudinally and dividing the first active cell panel into an upper section and a lower section,

wherein the plurality of apertures disposed on the upper section extend through the first active cell panel at an angle that is different from an angle at which the plurality of apertures disposed on the lower section extend through the first active cell panel, and

wherein the inner surface of the first active cell panel and at least one of the interior surfaces of the plurality of elongate apertures are coated with a photocatalytic material configured to exhibit a photocatalytic oxidative process when subjected to UV radiation emitted from the UV bulb;

a first side panel positioned on a third side of the housing and parallel with the longitudinal central axis, the first side panel comprising

a first reflective feature that includes a convex protrusion that protrudes from an inner surface of the first side panel inward into the inner cavity, the first reflective feature extending longitudinally on the inner surface of the first side panel for a length that coincides with the first active cell panel but is shorter than the first active cell panel so as to define an airway disposed at an end of the first reflective feature, the airway longitudinally coinciding with at least a portion of the first active cell panel to thereby allow a portion of the air passing through the active cell panel to pass through the airway uninterrupted by the first reflective feature, and

wherein the surface of the first reflective feature is configured to reflect UV radiation emitted from the UV bulb, in the direction of the first side panel, toward the inner surface of the first active cell panel

and at least one of the interior surfaces of the plurality of elongate apertures of the first active cell panel; and

a second side panel positioned on a fourth side of the housing and parallel with the longitudinal central axis.

14. The photocatalytic oxidation system of claim **1**, wherein the first reflective feature has a cross section perpendicular to the longitudinal central axis that comprises a mirror image about a first longitudinal plane perpendicular to the inner surface of the first side panel, the first longitudinal plane extending through the longitudinal central axis.

15. The photocatalytic oxidation system of claim **1**, wherein the plurality of apertures extend transversely through the first active cell panel at an angle of about 25 to 65 degrees relative to an axis perpendicular to the inner surface of the first active cell panel.

16. The photocatalytic oxidation system of claim **5**, wherein the second reflective feature extends longitudinally for a length that coincides with the first active cell panel but is shorter than the first active cell panel so as to define an airway disposed at an end of the second reflective feature, the airway longitudinally coinciding with at least a portion of the first active cell panel such that a portion of the air passing through the active cell panel can pass through the airway uninterrupted by the first reflective feature.

17. The photocatalytic oxidation system of claim **13**, wherein the first reflective feature has a cross section perpendicular to the longitudinal central axis that comprises a mirror image about a first longitudinal plane perpendicular to the inner surface of the first side panel, the first longitudinal plane extending through the longitudinal central axis.

18. The photocatalytic oxidation system of claim **13**, the second side panel comprising a second reflective feature that includes a convex protrusion that protrudes from an inner surface of the second side panel inward toward the longitudinal central axis, the second reflective feature extending longitudinally on the inner surface of the second side panel for a length that coincides with the first active cell panel but is shorter than the first active cell panel so as to define an airway disposed at an end of the second reflective feature, the airway longitudinally coinciding with at least a portion of the first active cell panel such that a portion of the air passing through the active cell panel can pass through the airway uninterrupted by the first reflective feature.

* * * * *
