# Supplementary material for: Evaluate the safety of a novel photohydrolysis technology used to clean and disinfect indoor air: A murine study
Source: PLoS One. 2024 Oct 9;19(10):e0307031. doi: 10.1371/journal.pone.0307031 (PMC11463749; doi:10.1371/journal.pone.0307031)
Supplement: S3 File — (PDF) [file pone.0307031.s003.pdf]

US00D974538S

(12) **United States Design Patent**
Eide

(10) **Patent No.: US D974,538 S**
(45) **Date of Patent: ** Jan. 3, 2023**

(54) **LOCKING BRACKET FOR AN AIR PURIFICATION DEVICE**

(71) Applicant: **DBG GROUP INVESTMENTS, LLC**, Dallas, TX (US)

(72) Inventor: **Andrew Eide**, Rockwall, TX (US)

(**) Term: **15 Years**

(21) Appl. No.: **29/721,677**

(22) Filed: **Jan. 22, 2020**

(51) **LOC (14) Cl.** **23-04**

(52) **U.S. Cl.**
USPC **D23/363**

(58) **Field of Classification Search**
USPC D8/382; D23/209, 230, 233–234, D23/262–266, 301, 341, 354, 358, 365; D25/2, 122–126, 133–134
CPC B01D 46/00; B01D 46/10; F02M 35/04; F02M 35/10; F02M 35/024
See application file for complete search history.

(56) **References Cited**

U.S. PATENT DOCUMENTS

D546,978	S	*	7/2007	Ye	D25/124
D622,836	S	*	8/2010	Deflorian	D23/366
D712,021	S	*	8/2014	You	D23/354
D874,619	S	*	2/2020	Simon	F24F 11/30 D23/262
D898,950	S	*	10/2020	Weber	D25/124
D910,158	S	*	2/2021	Kim	D23/365
D914,863	S	*	3/2021	Jeon	F24F 11/30 D23/364
D917,726	S	*	4/2021	Nelson	D25/124
D928,303	S	*	8/2021	Hanson	B01D 46/0001 D23/365
D938,567	S	*	12/2021	Du	B01D 46/0005 D23/365
D944,375	S	*	2/2022	Weiss	B32B 7/08 D23/354
D950,685	S	*	5/2022	Lan	D23/234
D950,776	S	*	5/2022	Shaffer	D25/126
D950,946	S	*	5/2022	Smith	D3/222
D953,582	S	*	5/2022	Sudo	D25/133
D954,546	S	*	6/2022	Smith	D8/382
D959,250	S	*	8/2022	Stahl	D8/382
D959,251	S	*	8/2022	Stahl	D8/382
2012/0317944	A1	*	12/2012	Lise	B01D 46/0001 55/499

(Continued)

Primary Examiner — Karen S Acker
Assistant Examiner — Jonathan E Fiencke
(74) *Attorney, Agent, or Firm* — Workman Nydegger; Logan Christenson; John Guynn

(57) **CLAIM**

The ornamental design for a locking bracket for an air purification device, as shown and described.

DESCRIPTION

FIG. **1** illustrates a perspective view of a locking bracket for an air purification device showing the new design;
FIG. **2** illustrates a front elevation view thereof;
FIG. **3** illustrates a rear elevation view thereof;
FIG. **4** illustrates a top plan view thereof;
FIG. **5** illustrates a bottom plan view thereof;
FIG. **6** illustrates a right side elevation view thereof;
FIG. **7** illustrates a left side elevation view thereof; and,
FIG. **8** illustrates a cross-sectional view thereof taken in the direction of line **8**-**8** in FIG. **2**.
The evenly spaced broken lines and unshaded areas depict portions of the locking bracket for an air purification device that form no part of the claimed design. The dot-dash-dot broken lines denote the boundaries of the claimed design and form no part thereof. In FIG. **2**, the dot-dash-dot broken lines denote the direction of the cross-sectional view of FIG. **8** and form no part of the claimed design.

1 Claim, 4 Drawing Sheets

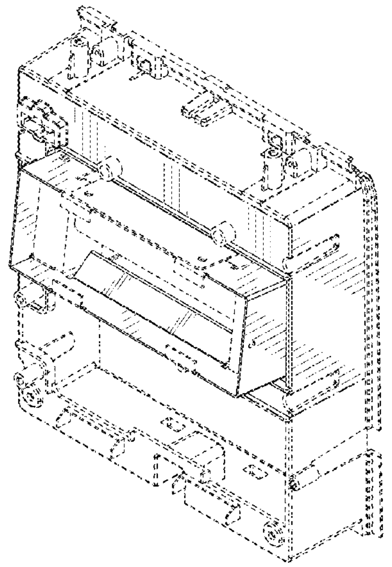

(56) **References Cited**

U.S. PATENT DOCUMENTS

2018/0161713	A1 *	6/2018	Cho	B01D 46/0005
2021/0162323	A1 *	6/2021	Hjertqvist	B01D 29/05

* cited by examiner

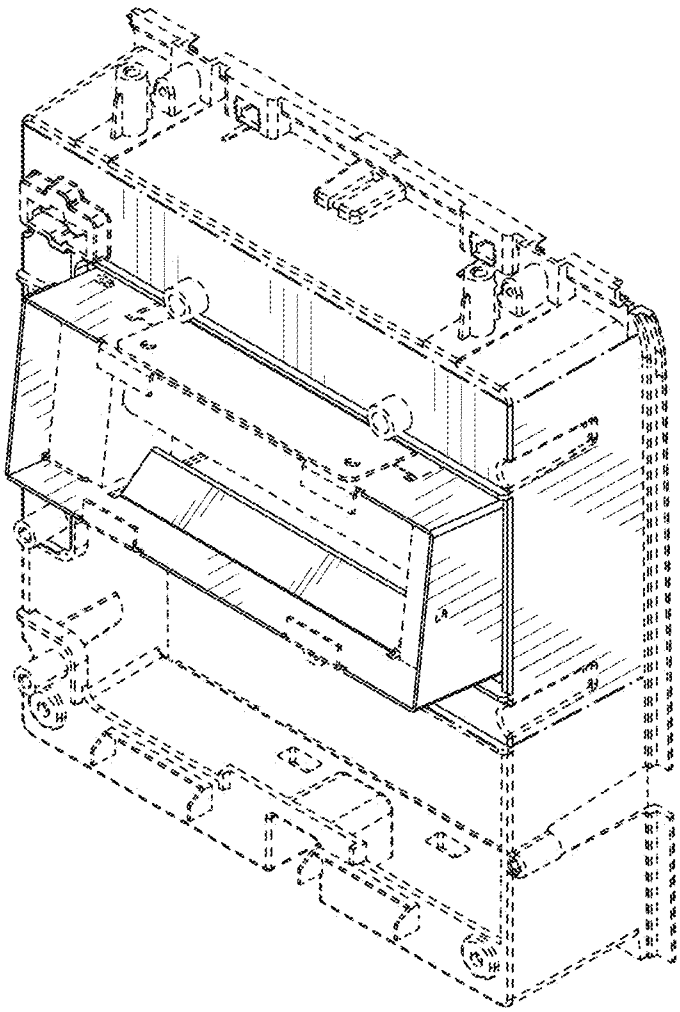

FIG. 1

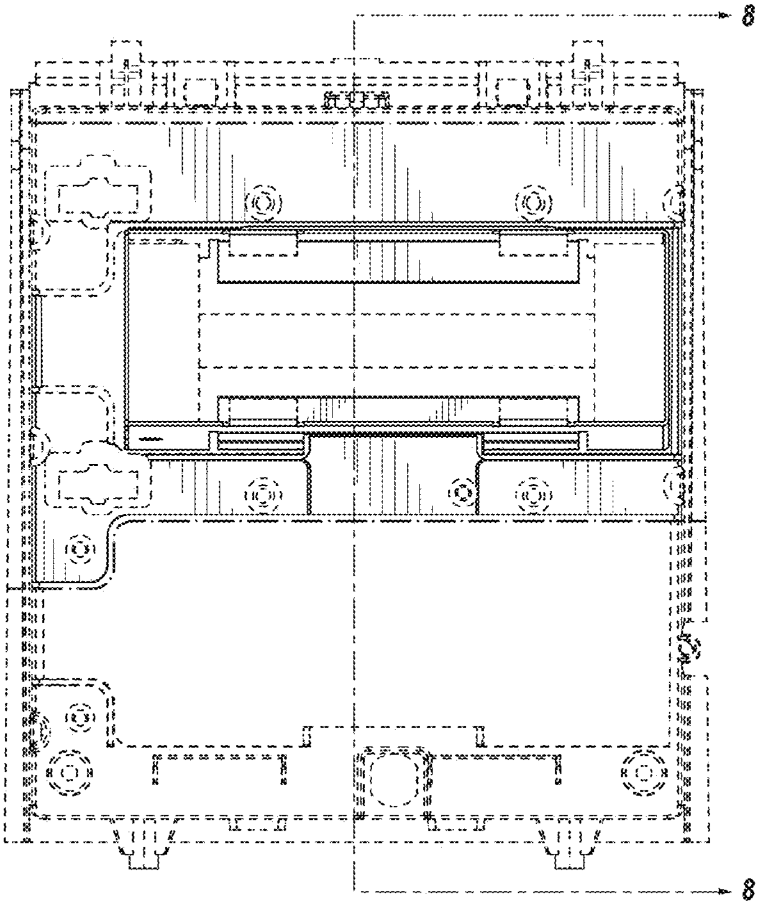

FIG. 2

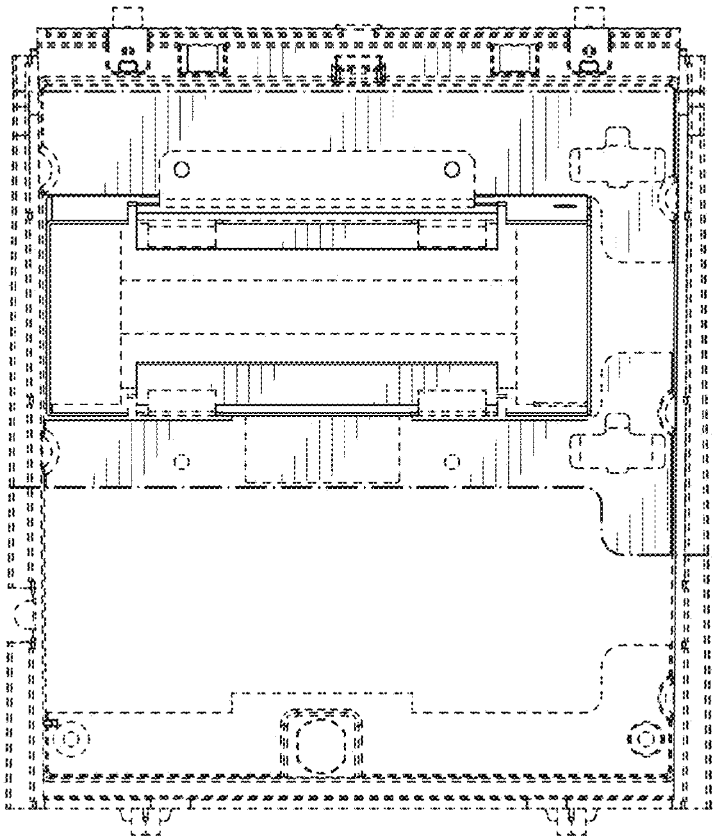

FIG. 3

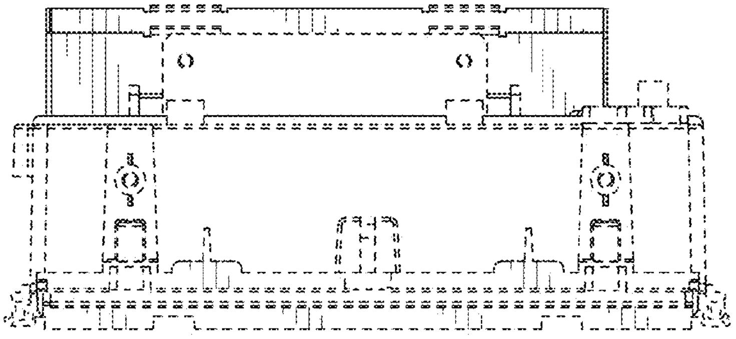

FIG. 4

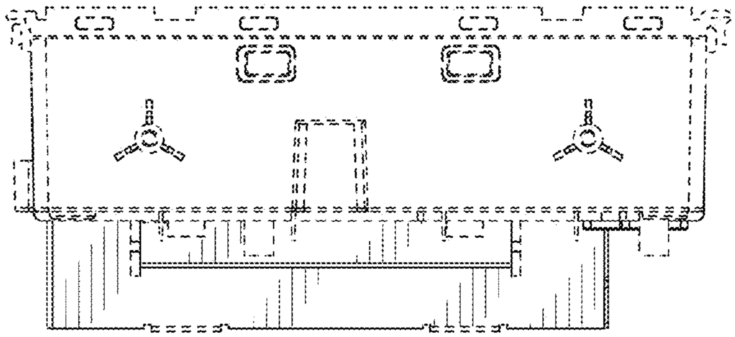

FIG. 5

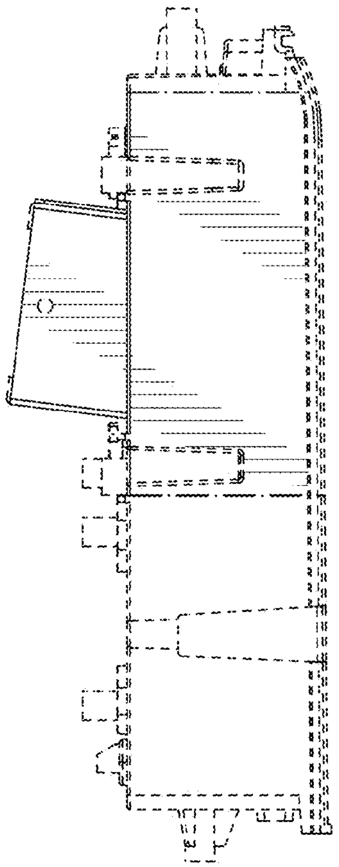

FIG. 6

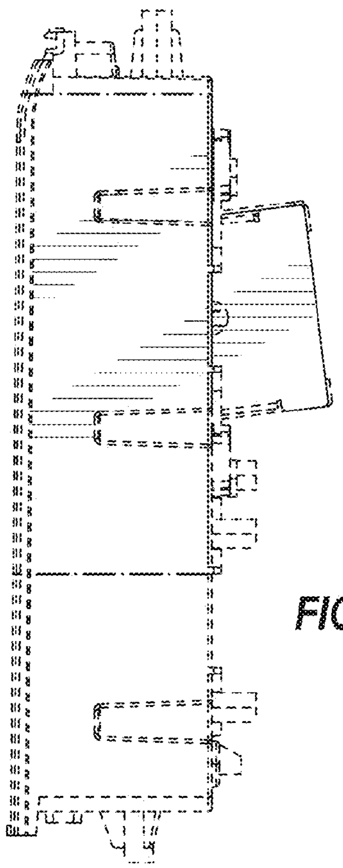

FIG. 7

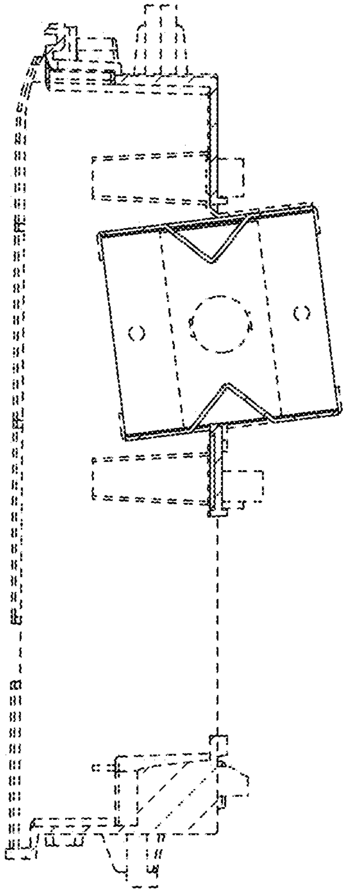

FIG. 8
