# Supplementary material for: Evaluate the safety of a novel photohydrolysis technology used to clean and disinfect indoor air: A murine study
Source: PLoS One. 2024 Oct 9;19(10):e0307031. doi: 10.1371/journal.pone.0307031 (PMC11463749; doi:10.1371/journal.pone.0307031)
Supplement: S4 File — (PDF) [file pone.0307031.s004.pdf]

Professional Environmental Services, Inc.

3092 Hull Avenue, Suite 201, Bronx, New York 10467 / Telephone (718) 231-8399 / Fax (718) 231-8109

EXECUTIVE SUMMARY FOR Particle Counts, TVOCs and Toxic Gasses:
On both inspections, IAQ Air readings were collected in the Lobby and inside the Lobby AC-10 Supply Fan. There was also an Outside Control Sample collected.

.

Lobby air results from both inspections were at levels far lower than the outdoor air. Therefore, all Particle Counts, TVOCs and Toxic Gas air results were negative.

Compared to the first inspection, the interior Total Volatile Organic Compound results were much lower on the second inspection.

Location	TVOC (Total Volatile Organic Compound Results) March 31st Inspection	TVOC (Total Volatile Organic Compound Results) April 20th Inspection
**Outside Control Sample**	1,870 ppb	1,990 ppb
**Lobby**	342 ppb	60 ppb
**Inside AC-10 Supply Fan-Lobby**	229 ppb	36 ppb

Above: TVOC. (Total Volatile Organic Compound Results)
Compared to the first inspection, the interior Total Volatile Organic Compound results were lower on the second inspection.

1301 6th AVE

Professional Environmental Services, Inc.
3092 Hull Avenue, Suite 201, Bronx, New York 10467 / Telephone (718) 231-8399 / Fax (718) 231-8109

EXECUTIVE SUMMARY FOR Particle Counts, TVOCs and Toxic Gasses:
On both inspections, IAQ Air readings were collected in the Lobby and inside the Lobby Air Handler. There was also an Outside Control Sample collected.

Lobby air results from both inspections were at levels far lower than the outdoor air. Therefore, all Particle Counts, TVOCs and Toxic Gas air results were negative.

Compared to the first inspection, the interior Total Volatile Organic Compound results were much lower on the second inspection.

Location	TVOC (Total Volatile Organic Compound Results) March 31st Inspection	TVOC (Total Volatile Organic Compound Results) April 29th Inspection
**Outside Control Sample**	856 ppb	789 ppb
**Lobby**	221 ppb	88 ppb
**Air Handler Lobby**	148 ppb	46 ppb

Above: TVOC. (Total Volatile Organic Compound Results)
Compared to the first inspection, the interior Total Volatile Organic Compound results were lower on the second inspection.

1633 Broadway

Professional Environmental Services, Inc.

3092 Hull Avenue, Suite 201, Bronx, New York 10467 / Telephone (718) 231-8399 / Fax (718) 231-8109

EXECUTIVE SUMMARY FOR Particle Counts, TVOCs and Toxic Gasses:
On both inspections, IAQ Air readings were collected in the Lobby 53rd Street, Lobby 54th Street, Elevator Lobby, 4th Floor return, and an Outside Control Sample.

Lobby air results from both inspections were at levels far lower than the outdoor air. Therefore, all Particle Counts, TVOCs and Toxic Gas air results were negative.

Compared to the first inspection, the interior Total Volatile Organic Compound results were much lower on the second inspection.

Location	TVOC (Total Volatile Organic Compound Results) September 22nd Inspection	TVOC (Total Volatile Organic Compound Results) September 30th Inspection	TVOC (Total Volatile Organic Compound Results) November 1st Inspection
**Outside Control Sample**	1,980 ppb	1,890 ppb	2,360 ppb
**Lobby 53rd Street**	676 ppb	88 ppb	106 ppb
**Lobby 54th Street**	843 ppb	90 ppb	89 ppb
**Elevator Lobby**	833 ppb	134 ppb	98 ppb
**4th Floor Return**	1,230 ppb	1,764 ppb (Open outside Air Louvers)	1,432 ppb (Open outside Air Louvers)

Above: TVOC. (Total Volatile Organic Compound Results)
Compared to the first inspection, the interior Total Volatile Organic Compound results were much lower on the second and third inspections.

Like the second inspection, we did have the open outside louvers on the third inspection.
