# Supplementary material for: Evaluate the safety of a novel photohydrolysis technology used to clean and disinfect indoor air: A murine study
Source: PLoS One. 2024 Oct 9;19(10):e0307031. doi: 10.1371/journal.pone.0307031 (PMC11463749; doi:10.1371/journal.pone.0307031)
Supplement: S5 File — (PDF) [file pone.0307031.s005.pdf]

14841 Dallas Parkway
Suite 500, The Aberdeen Bldg.
Dallas, Texas 75254 USA

Performance Testing – Bench

Efficacy of Aerus Medical Guardian Air System against Various Bioaerosols

Device Name: Aerus Medical Guardian
Model: F170A

Result Summary:

The Aerus Medical Guardian, model F170A device was tested by Aerosol Research and Engineering Laboratories Inc., located in Olathe Kansas to evaluate the reduction of aerosolized microorganisms from the air at 72°F, 50% RH as listed in **Table 1** on the following page. For these studies, specific types of viruses, bacteria and spores were selected to provide a representative sampling of organism types to evaluate the effectiveness of the Aerus Medical Guardian, model F170A for the reduction of those types (DNA, RNA, Gram "+", Gram "–" ,bacterial and fungal spores) of aerosolized organisms from the air.

The device used by Aerosol Research and Engineering Laboratories Inc for evaluation was preconditioned to evaluate performance with the device components at their end of life. All components, filter, Active Pure cell which includes 254 nm UVGI Bulbs and honeycomb coated with the TiO2 catalyst, power switch, ionizer, circuit board and fan had been previously used in various extreme tests either in devices or as separate components. Preconditioned components were then built into devices, and placed in a professional healthcare environment, operating for more than 6 months with the fan set to its highest speed, which is worst case condition, exposing the devices to conditions it would be exposed to with the device intended use.

Unlike the other device components, the filter and cell are considered replaceable parts and have a shorter usage life. 6 months of operation is the recommended replacement time for the filter, so 6 months use in a professional healthcare environment with a filter at it end of life is worst case. The Active Pure cell which contains the two UVGI bulbs and TiO2 coated honeycomb has a recommended replacement time of 1 year of operation. The UVGI bulbs used had been life tested for 9,000 hours. The cell was operated in a 73°F and 60%RH ventilated environment for 6 months. After 6 months of operation, cells were then installed in devices that underwent further use in a professional healthcare environment for an additional 6 months with a cell at its end of life is worst case.

After an additional 6 months of operation in a professional health care environment was achieved, a device was removed, fully evaluated ensuring no operational issues occurred. It was then packaged and shipped to Aerosol Research and Engineering Laboratories Inc to be used in testing. Device tested was at its end of life as a device and the components which make up the device to represent worst care.

AERUS

14841 Dallas Parkway
Suite 500, The Aberdeen Bldg.
Dallas, Texas 75254 USA

Table 1:

Organism Type	Species	Organism Name	Test Temp/RH	Exposure Time (m)	Avg Log-Reduction
Bacteria (gram +)	ATCC 12228	Staphylococcus epidermidis	72°F/50%	60	5.95
Bacteria (gram -)	ATCC 27155	Erwinia herbicola	72°F/50%	60	5.12
Virus (RNA)	ATCC 15597- B1	MS2	72°F/50%	60	5.58
Virus (DNA)	ATCC 13706-B1	Phi-X174	72°F/50%	60	4.19
Fungal spore	ATCC 13835	Aspergillus niger	72°F/50%	60	4.12
Bacterial spore	ATCC 16404	Bacillus globigii	72°F/50%	60	4.22

To summarize, the Aerus Medical Guardian, model F170A device used in bioaerosol tests, Table 1 is a device with, device components, including a filter and Active Pure cell at the end of their useful life, but still fully functional and previously operated in a professional healthcare environment. Professional healthcare environmental conditions are well controlled in a range of 68°F to 72°F. Testing of the device was performed at the upper end of the environmental conditions found in the professional healthcare environment. Test conditions as indicted in Table 1, should be sufficient demonstrating bio organism reduction without additional testing under conditions beyond the device's intended use. Our scientific rational for this position is;

1. The intended use of the device is in a controlled environment of 70°F~71°F, 40~45%RH and in its intended use would not be exposed to extreme conditions.
2. Professional healthcare environments are well controlled within 68°F to 72°F,40~60%RH and as such, the device would not be subjected to extreme conditions in those environments.
3. The device, device components, transportation & handling of the device have undergone extensive useful life and performance testing in actually extreme conditions, far in excess of what the device would be exposed to in its intended use.
4. ARE Labs letter supporting of position that slight temperature and humidity variation would have no measurable impact on either filter capture efficiency, kill rate or reduction performance of the device. Position letter is in section 22, pages 22.56 ~ 22.58
5. Testing in environmental conditions a degree or two above or below the device intended use would have inconsequential effect on the tested bio organisms, supported by test data from another FDA cleared device, Transformair, K161468 which has a same intended use as the Aerus Medical Guardian,. Model F4170A, tested at 45°F, 72°F and 110°F using the six (6) types of bio organisms that was used for the Aerus Medical Guardian, model F170A device testing. The range of environmental conditions used on Transformair testing was based on its application in an HVAC environment. The Transformair test result are shown in table 2.

AERUS

14841 Dallas Parkway
Suite 500, The Aberdeen Bldg.
Dallas, Texas 75254 USA

Table 2:

Organism	Name	Average Maximum log reduction /exposure time (hours)		
		Test temperature		
		45°F	72°F	110°F
Bacteria	Staphylococcus	4.88 / 24 hours	4.02 / 0.33 hours	4.20 / 0.33 hours
Bacteria	Escherichia coli	4.31 / 24 hours	4.79 / 24 hours	4.40 / 0.33 hours
Virus	MS2 bacteriophage	4.13 / 24 hours	4.25 / 24 hours	5.51 / 24 hours
Virus	Phi-X174	4.37 / 24 hours	4.37 / 24 hours	4.37 / 24 hours
Mold endospore	Aspergillus Niger	3.91 / 72 hours	3.99 / 72 hours	4.22 / 72 hours
Bacterial endospore	Bacillus globigii	4.11 / 72 hours	4.41 / 72 hours	4.41 / 72 hours

Based on review of available study information, our position and that of ARE Labs is environmental temperature conditions used for the Transformair device study had inconsequential effect in the overall reduction of tested bio organisms. Reduction in the Transformair study appears the result of the device operation, not the environmental temperature conditions.

As such, further studies at environmental conditions below or above the 72°F used in the Aerus Medical Guardian study would be unnecessarily burdensome as those Aerus Medical Guardian, model F170A results would be predictably similar, an inconsequential effect, to what the Transformair study at the extreme conditions has shown.

This position is further supported by the expert medical opinion of Dr. Daniel I. Sessler M.D. Dr. Sessler attended medical school at Columbia University, and subsequently completed pediatric and anesthesia residencies at the University of California, Los Angeles. He is currently Michael Cudahy Professor and Chair of the Department of Outcomes Research at the Cleveland Clinic.

The Outcomes Research Consortium (www.OR.org), which Dr. Sessler founded, includes more than 120 investigators in ten countries. The Outcomes Research Consortium currently coordinates more than one hundred studies, including a dozen large, multi-center outcome trials. Dr. Sessler has published a book on therapeutic hypothermia and more than 600 full research papers; 200 of his papers were in Anesthesiology, and a dozen were in the New England Journal of Medicine or Lancet. His papers have been cited more than 25,000 times.

Dr. Sessler's has trained more than 100 research fellows, five of whom subsequently chaired anesthesia departments. Among his awards is a Fulbright Fellowship and the 2002 American Society of Anesthesiology Excellence in Research prize.

Dr.Sessler’s administrative work includes two stints as acting-chair of the Department of Anesthesiology at the University of Louisville. He was also Vice-dean and Associate Vice President for Health Affairs at the University of Louisville, giving him responsibility for research throughout the Health Sciences Campus.

Dr. Sessler’s opinion letter is in section 22, pages 22.59 ~ 22.60

The complete Aerus Medical Guardian, model F170A test report on **Efficacy of Aerus Medical Guardian Air System against Various Bioaerosols** is detailed in section 19, pages 19.32 through 19.71. The Aerus Medical Guardian, model F170A device test results demonstrate an average log-reduction of the six aerosolized bio organisms listed in Table 1, to be 4.9 after one hour as tested.
