# Supplementary material for: Evaluate the safety of a novel photohydrolysis technology used to clean and disinfect indoor air: A murine study
Source: PLoS One. 2024 Oct 9;19(10):e0307031. doi: 10.1371/journal.pone.0307031 (PMC11463749; doi:10.1371/journal.pone.0307031)
Supplement: S6 File — (PDF) [file pone.0307031.s006.pdf]

Efficacy of Aerus Medical Guardian Air System against Various Bioaerosols

Jamie Balarashti [a], Sylvester Marshall III [a], Dan Merchant [a]

[a] Aerosol Research and Engineering Laboratories Inc. Olathe KS

Background: This in-vitro study characterized the decontamination efficacy of the Aerus Medical Guardian against various aerosolized biologicals. Aerus Medical LLC's (Bristol, VA) Medical Guardian unit is an indoor air purification system that is designed to neutralize airborne microorganisms. The effectiveness of the system was assessed for six (6) aerosolized biologicals: two vegetative bacteria: *Staphylococcus epidermidis (Gram +)* and *Erwinia herbicola (Gram -);* two viruses: MS2 (RNA bacteriophage) and Phi X174 (DNA bacteriophage); a bacterial endospore; *Bacillus globigii*, and a mold spore; *Aspergillus niger*. The testing consisted of a single control trial plus triplicate decontamination trials for each organism tested.

Methods: Each of the six (6) organisms used for testing were nebulized into a sealed environmental bioaerosol chamber containing the Aerus Medical Guardian air system. AGI impingers were used to capture chamber bioaerosol concentrations at set sampling times. Viable cascade impactors were utilized with some organisms for higher resolution sample collection. All impinger samples were serially diluted, plated and enumerated in triplicate to yield viable bioaerosol concentration at each sampling point and time. Chamber control trial data was subtracted from Medical Guardian trial data to yield net LOG reduction in the chamber for each tested bioaerosol challenge.

Results: *Staph* had an average Net LOG reduction of 5.46 +/- 0.34 for the triplicate trials. *Erwinia's* average Net LOG reduction was 5.36 +/- 0.37. The Medical Guardian's efficacy against the MS2 virus was 5.58 +/- 0.43. The performance against Phi X174 showed 4.05 +/- 0.27 net LOG reduction. The endospores, *Bacillus*, had an average net LOG reduction of 4.23 +/- 0.31, and the mold spores, *A. niger*, had a net LOG reduction of 4.12 +/- 0.10. The two test viruses, MS2 and Phi X174, organisms showed no viability after t=75minute (MS2) and T=60minutes (PhiX174), in order to calculate the net LOG kill for these organisms a fictitious single colony count was used to calculate the limits of detection for each trial and to show the minimum Net LOG reduction achieved for the Medical Guardian.

Summary: Overall, the Medical Guardian showed an average Net LOG reduction for all organisms tested of 4.80 +/- 0.74. The unit seemed most effective at removing vegetative bacteria from an environment, with an average net LOG reduction of the two vegetative bacteria tested of 5.41 +/- 0.07. However both viruses were below detectable limits due there being zero plaques observed on plates after a certain time point, meaning that the net LOG reduction for those organisms only represents a minimum value. Spores are known to be tough and highly resilient, and therefore it is not unexpected that their net LOG reductions, average 4.18 LOG, are not quite as high as the other organisms. Overall the Medical Guardian showed a high efficacy against of a broad range of viable bioaerosol.

This study was conducted in compliance with FDA Good Laboratory Practices (GLP) as defined in 21 CFR, Part 58.

Overview

This study was conducted to evaluate the efficacy of the Aerus Medical Guardian produced by Aerus Medical LLC's (Bristol, VA), to decontaminate viable airborne bioaerosols. Testing was conducted in a controlled stainless steel aerosol chamber designed to simulate a small room environment. The Medical Guardian's effectiveness was tested against six (6) Bio-safety level 1 (BSL1) organisms in order to evaluate the system's Net LOG reduction of the viable bioaerosols.

All Bioaerosol challenge testing for this study used an Aerus Medical Guardian (Model # F170A) developed by Aerus Medical LLC (Bristol, VA). A picture and details of the Medical Guardian air system is shown in **Figure 1.**

The effectiveness of the Medical Guardian was evaluated against vegetative bacterium, endospores, viruses, and mold spores. Testing with each of the six distinct organisms was completed in triplicate trials plus a control trial to demonstrate the capability of reducing viable bioaerosol concentrations. There were a total of twenty-four (24) independent trials in this study.

Each organism was examined during a set process involving a control trial and three test trials. During the control trials the Medical Guardian would remain inside the testing chamber, but would never be activated. The organisms were aerosolized into the controlled chamber, and air samples were collected at set time points throughout each trial. Comparisons of the number of living organisms between the control trials and the test trials allowed for determination of the Medical Guardian's efficacy at removing viable bioaerosols from an enclosed room environment.

AERUS

Aerus Medical Guardian

Picture:

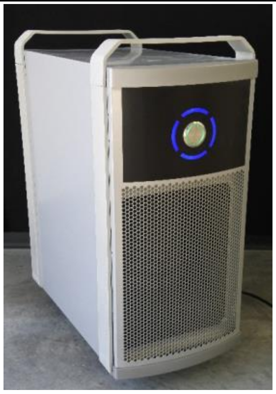

Device Features	
Manufacturer:	Aerus Medical LLC
Model:	F170A
Notes:	Ion Generator, Photocatalytic Oxidizer

Figure 1: *Aerus Medical Guardian air system*

Bioaerosol Testing Chamber

A large sealed aerosol test chamber was used to replicate a potentially contaminated room environment and to contain any potential release of aerosols into the surrounding environment.

The aerosol test chamber is constructed of 304 stainless steel and is equipped with three viewing windows and an air-tight lockable chamber door for system setup and general ingress and egress. The test chamber internal dimensions are 9.1ft x 9.1ft x 6.8ft, with a displacement volume of 562 cubic feet, or 15,914 liters. **Figure 2** shows a diagram of the testing chamber configuration. Testing conditions inside the chamber were consistently 72.0°F with 50% relative humidity.

The chamber is equipped with filtered HEPA inlets, digital internal temperature and humidity monitor, external humidifiers (for humidity control), lighting system, multiple sampling ports, aerosol mixing fans, and a HEPA filtered exhaust system that are operated with wireless remote control. For testing, the chamber was equipped with four 3/8 inch diameter stainless steel probes for aerosol sampling each sampling about 18" from the nearest sidewall. A stainless 1 inch diameter port was used for bio-aerosol dissemination into the chamber using a Collison 24-jet nebulizer for the bacteriophages, vegetative cells and bacterial spores, or a dry powder eductor for the fungal spores.

A ¼ inch diameter probe was used for continuous aerosol particle size monitoring via a TSI Aerodynamic Particle Sizer (APS) model 3321. All sample and dissemination ports were inserted approximately 18 inches from the interior walls of the chamber to avoid wall effects and at a height of approximately 40 inches from the floor.

The aerosol sampling and aerosol dissemination probes are stainless steel and bulk headed through the chamber walls to provide external remote access to the aerosol generator and samplers during testing.

The test chamber is equipped with two high-flow HEPA filters for the introduction of filtered purified air into the test chamber during aerosol evacuation/purging of the system between test trials and a HEPA filtered exhaust blower with a 500 ft^3/min rated flow capability for rapid evacuation of remaining bioaerosols.

A magnehelic gauge with a range of 0.0 +/- 0.5 inch H_2O (Dwyer instruments, Michigan City IN) was used to monitor and balance the system pressure during aerosol generation, aerosol purge and testing cycles.

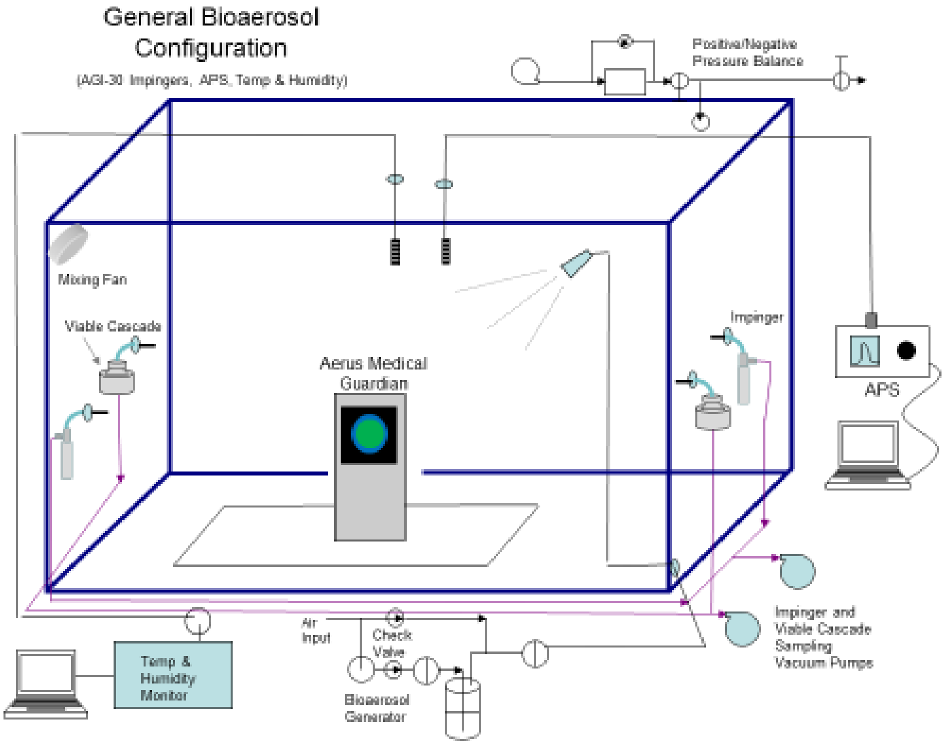

Figure 2: *Bio-Aerosol Test Chamber Flow Diagram.*

Test Location and Conditions

Testing was conducted at Aerosol Research and Engineering labs located at 15320 S. Cornice Street in Olathe, Kansas 66062. Laboratory conditions were approximately 70.6°F with 36% relative humidity.

Bioaerosol Sampling and Monitoring System

Two AGI-30 impingers (Ace Glass Inc., Vineland NJ) were used for bio-aerosol collection of biological aerosols to determine the chamber concentration. These impingers were connected to the bioaerosol chamber via sample ports located at opposite corners of the chamber.

The AGI-30 impinger vacuum source was maintained at a negative pressure of 18 inches of Hg during all characterization and test sampling to assure critical flow conditions. The AGI-30 sample impingers were flow characterized using a calibrated TSI model 4040 mass flow meter.

Aerosol particle size distributions and count concentrations were measured in real-time through the duration of all control and Aerus trial runs using a model 3321 Aerodynamic Particle Sizer (APS) (TSI Inc., St Paul, MN). The APS sampled for the entire duration of all trials (90 minutes) with 1 minute sampling intervals.

The impingers were filled with 20 mL of sterilized PBS (addition of 0.005% v/v Tween 80) for bioaerosol collection. The addition of Tween 80 was shown to increase the impinger collection efficiency and de-agglomeration of all microorganisms.

During testing with some organisms, sample collections were also obtained using a pair of viable cascade impactors. A viable cascade impactor (SKC Inc., Valley View, PA) comprises an inlet cone, precision-drilled 400-hole impactor stage, and a base that holds a standard-size agar plate (**Figure 3**). A high flow pump pulls microorganisms in air through the holes (jets) where they are collected on the agar surface.

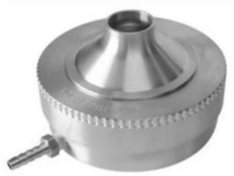

Figure 3: SKC BioStage Viable Cascade Impactor.

Trial	Run	Species (gram, description)	ATCC Ref	Target Monodispersed Particle Size	Challenge Conc. (#/L)	Total Trial Time (min)	Impinger Sample Time (min)	Sampling	Plating and Enumeration
1<br>2	Control<br>Challenge	*Stapilococcuss epidermidis*<br>(+, vegetative)	12228	2.5-3.0μm	10^4-10^6	90	0, 15, 30, 45, 60, 75, 90	APS, Impingers & Viable Cascade	all samples in triplicate
3<br>4	Control<br>Challenge	*Erwinia herbicola*<br>(-, vegetative)	27155	2.5-3.0μm	10^4-10^6	90	0, 15, 30, 45, 60, 75, 90	APS, Impingers & Viable Cascade	all samples in triplicate
5<br>6	Control<br>Challenge	*MS2 bacteriophage*<br>(E. coli phage)	15597-B1	<50 nm	10^4-10^6	90	0, 15, 30, 45, 60, 75, 90	APS, Impingers	all samples in triplicate
7<br>8	Control<br>Challenge	*Aspergillus niger*<br>(mold, spore forming)	13835	<5.0μm	10^4-10^6	90	0, 15, 30, 45, 60, 75, 90	APS, Impingers	all samples in triplicate
9<br>10	Control<br>Challenge	*Bacillus globigii* endospore<br>(*Bacillus* Spores)	16404	<3.5 μm	10^4-10^6	90	0, 15, 30, 45, 60, 75, 90	APS, Impingers & Viable Cascade	all samples in triplicate
11<br>12	Control<br>Challenge	Phi X174 bacteriophage<br>(E. coli *phage)*	13706-B1	<50 nm	10^3-10^5	90	0, 15, 30, 45, 60, 75, 90	APS, Impingers & Viable Cascade	all samples in triplicate

Table 1: *Bioaerosol Test Matrices for all trials.*

This method of bioaerosol collection was chosen as the most sensitive and accurate sampling process for the quantification of bioaerosols with this testing configuration. With viable collection enumeration detection at one colony forming unit (cfu), direct collection onto nutrient agar, and immediate incubation after sample collection, it provides the highest sensitivity for low concentration viable aerosol collection and measurement. This method of collection was not a feasible option for all organisms due to the plating methods of viruses and growth patterns of mold spores. **Table 1** shows the complete test matrix for this study.

Bioaerosol Generation System

Test bioaerosols were disseminated using a Collison 24-jet nebulizer (BGI Inc. Waltham MA) driven by purified filtered house air supply. A pressure regulator allowed for control of disseminated particle size, use rate and sheer force generated within the Collison nebulizer. The mold spore organisms were disseminated using a dry powder aerosolization unit.

Prior to testing, the Collison nebulizer flow rate and use rate were characterized using an air supply pressure of approximately 60 psi, which obtained an output volumetric flow rate of 50-80 lpm with a fluid dissemination rate of approximately 1-2 ml/min. The Collison nebulizer was flow characterized using a calibrated TSI model 4040 mass flow meter (TSI Inc., St Paul MN).

Species Selection

Species selection is based on Biological Safety Level 1 (BSL1) surrogates for a wide range of BSL3 pathogenic organisms. It is routine in the bioaerosol field to use surrogate species to test performance against BSL3 organism decontamination due to the high cost and limited lab space associated with aerosol BSL3 testing. For this reason a broad range of viable bioaerosol surrogates were selected to specifically test the Guardian’s efficacy against various types of pathogenic bioaerosols encounter in hospital and other environments.

Staphylococcus epidermidis (Gram +, ATCC 12228) acts as a surrogate for its cousin, *Staph aureus,* which has developed multi drug resistance (MRSA) and is one of the major leading causes of hospital-acquired infections.

Erwinia Herbicola (Gram -, ATCC 27155) is a Gram-negative bacterium and has been used historically as a surrogate for black plague causing bacterium, *Yersinia pestis*.

MS2 (ATCC 15597-B1) is a viral RNA bacteriophage that is commonly used as a surrogate for the influenza virus and the norovirus.

PhiX-174 (ATCC 13706-B1) is a small, single-stranded DNA virus that is often used as a surrogate for HCV, HCB, and HIV in research studies.

Aspergillus niger (ATCC 13835) endospores are used as a surrogate for several toxic black mold species such as *Stachybotrys chartarumand*.

Bacillus globigii (now named *Bacillus subtillis*) endospores (ATCC 16404) are routinely used as a surrogate for weaponized anthrax, *Bacillus anthracis*. Phi X174

Vegetative Cells Culture & Preparation

Pure strain seed stocks were purchased from ATCC (American Type Culture Collection, Manassas VA). Working stock cultures were prepared using sterile techniques in a class 2 biological safety cabinet and followed standard preparation methodologies. Approximately 100ml of *Staph* and *Erwinia Herbicola* stock was prepared in tryptic soy liquid broth media, and incubated for 24 hours with oxygen infusion (1cc/min) at 37°C. Biological stock concentrations were greater than 1 x 10^9 cfu/ml for *Staph* and *Erwinia* using this method.

Stock cultures were centrifuged for 12 minutes at 4000 rpm in sterile 50mL conical tubes, growth media was decanted, and the cells re-suspended in sterile PBS buffer for aerosolization. Aliquots of these suspensions were enumerated on tryptic soy agar plates (Hardy Diagnostics, Cincinnati OH) for viable counts and stock concentration calculations. For each organism, test working stocks were grown in sufficient volume to satisfy use quantities for all tests conducted using the same culture stock material.

Viral Culture & Preparation

Pure strain viral seed stock and host bacterium were obtained from ATCC. Host bacterium was grown in a similar fashion to the vegetative cells in an appropriate liquid media. The liquid media was infected during the logarithmic growth cycle with the specific bacteriophage. After an appropriate incubation time the cells were lysed and the cellular debris discharged by centrifugation. MS2 stock yields were greater than 1 x 10^9 plaque forming units per milliliter (pfu/ml) with a single amplification procedure. Phi X174 stock yields were less than MS2 yields, and ranged between 1 x 10^8 to 1 x 10^9 pfu/ml after two amplifications.

Endospore Culture & Preparation

Bacillus globigii spores were growth, sporelated, spray dried and stored in a pure dry powder (2.41 x 10^{11} cfu/gr.) form ahead of time. Master stocks were prepared using the pure spore and were kept stored in a 30% ethanol : 70% PBS + tween solution to maintain their endospore state and to prevent contamination by other vegetative cells. Nebulization proceed directly using this master stock.

Aspergillus niger fungal spores were also cultured, purified and stored in purified bulk powder form with a concentration of 3.4 x 10^9 cfu/gr. Due to the size of the spore (5µm diameter) nebulization was not used for dissemination of the bioaerosol, instead a custom dry eductor and feeder was used dispersed into the chamber using a ARE Labs dry powder aerosolization unit.

Plating and Enumeration

Impinger and stock biological cultures were serially diluted and plated in triplicate (multiple serial dilutions) using a standard spread plate assay technique onto tryptic soy agar plates. The plated cultures were incubated for 24-48 hours (species dependent) and enumerated and recorded.

Inert Particle Characterization

In order to calculate the dissemination efficiency, stability and to pinpoint impinger/viable cascade sample times pre-testing was conducted using polystyrene latex beads (PSL miscrospheres) prior to bioaerosol testing. PSL microspheres were used to characterize the various aspects of the chamber and Aerus system.

Polydispersed PSL beads with aerodynamic diameters of 1.0µm were nebulized and chamber concentrations were recorded using the APS. The control trials were used to calculate nebulization efficiencies, particle stability and AGI-30 collection efficiencies were used to estimate generation efficiencies, dissemination times, sample times and aerosol persistence prior to bioaerosol testing. Live trials with the Medical Guardian system were also performed to measure particle removal rates of the system.

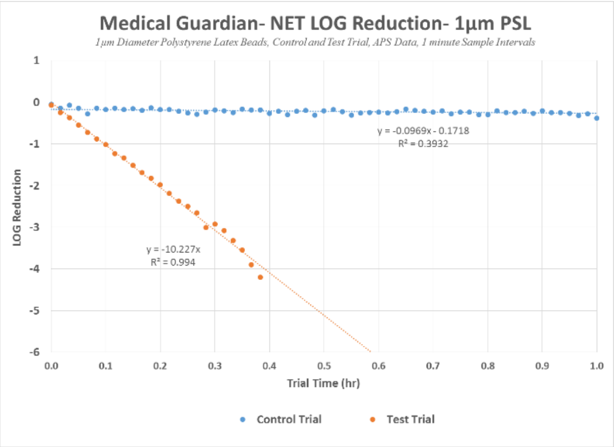

***Figure 4:** LOG Reduction of 1.0um PSL Beads*

Figure 4 shows the LOG reduction of 1.0um PSL beads both with the Medical Guardian unit on (Test Trial) and off (Control Trial). The figure shows the

General Timeline for Bioaerosol Chamber Testing

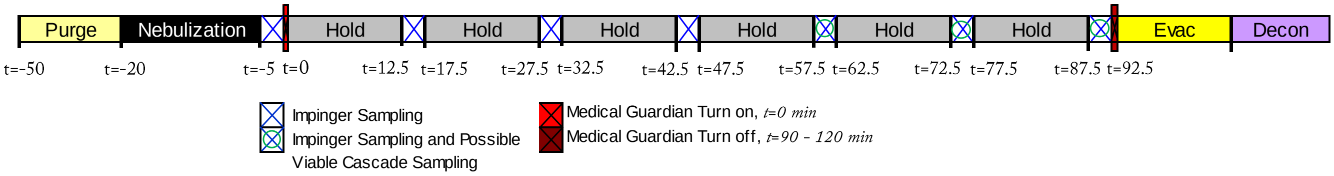

Table 2: General *Trial Timeline for Aerus Medical Guardian Decontamination Trials.*

comparison between the reduction with and without the Medical Guardian. When the Guardian is turned on the LOG reduction of 1.0um PSL beads follows a precise logarithmic function. It is notable that this size (1um) is smaller than the vegetative bacteria, bacterial endospores, and mold spores, but larger than the two viruses.

Control Testing

To accurately assess the Aerus Medical Guardian unit, test chamber pilot control trials were performed with all Bioaerosols from 90 to 120 minute periods without the Medical Guardian in operation to characterize the biological challenge aerosol for particle size distribution, aerosol delivery/collection efficiency, decay rate and viable concentration over time. Control testing was performed to provide baseline comparative data in order to assess the actual reduction from Medical Guardian challenge testing and verify that viable bioaerosol concentrations persisted above the required concentrations over the entire pilot control test period.

During control runs, a single low velocity fan located in the corner of the bioaerosol test chamber was turned on for the duration of trial to ensure a homogenous aerosol concentration within the aerosol chamber. The mixing fan was used for all control runs and was turned off during Medical Guardian decontamination trials. The two impingers used for bacteriophage, vegetative, fungal and bacterial endospore test sampling were pooled and mixed prior to plating and enumeration.

Medical Guardian Testing

For each control and challenge test, the Collison nebulizer was filled with approximately 50 mL of biological stock and operated at 50 psi for a period of 20 or 25 minutes (organism dependent). For control and Medical Guardian trials, the impingers were filled with 20 mL of sterilized PBS (addition of 0.005% v/v Tween 80) for bioaerosol collection. The addition of Tween 80 was shown to increase the impinger collection efficiency and de-agglomeration of all microorganisms.

The chamber mixing fan was turned on during bioaerosol dissemination to assure a homogeneous bioaerosol concentration in the test chamber prior to the first impinger sample.

Following bioaerosol generation, baseline bioaerosol concentrations were established for each pilot control and Guardian test by sampling simultaneously with two AGI-30 impingers located at opposite corners of the chamber. AGI samples were collected for 2, 5, or 10 minutes (organism dependent) at intervals of 15 minutes throughout the entire period. **Table 2** above shows the general timeline for each Medical Guardian live bioaerosol challenge trial.

Collected impinger chamber samples were pooled and mixed at each sample interval for each test. Aliquots of impinger samples were collected and then used for plating. Impingers were rinsed 6x with sterile filtered water between each sampling interval, and re-filled with sterile PBS using sterile graduated pipettes for sample collection.

For Medical Guardian biological testing, the unit was turned on immediately following a time 0 baseline sample and operated for the entirety of the test (up to 90 minutes). Subsequent impinger samples were taken at intervals of 15 minutes and samples enumerated for viable concentration to measure the effective viable bioaerosol reduction during operation of the Medical Guardian system over time. **Table 2** outlines the general timeline for the testing procedure with the Medical Guardian.

Test chamber temperature and humidity were recorded at the initiation and completion of each test.

All samples were plated in triplicate on tryptic soy agar media over a minimum of a 2 log dilution range.

Plates were incubated for viable plaque forming units (pfu) formation for the viral phase of the study, and colony forming units (cfu) for fungal spore, and bacterial endospore phases of the study. Plates were incubated and enumerated for viable counts to calculate aerosol challenge concentrations in the chamber and reduction of viable microorganisms.

Post-Testing Decontamination and Prep

Following each test, the chamber was air flow evacuated/purged for a minimum of twenty minutes between tests and analyzed with the APS for particle concentration decrease to baseline levels between each test. The chamber was decontaminated between live microorganism trials with aerosol/vaporous hydrogen peroxide (35%). The Collison nebulizer and impingers were cleaned at the conclusion of each day of testing by soaking in a 5% bleach bath for 20 minutes. The nebulizer and impingers were then submerged in a DI water bath, removed, and spray rinsed 6x with filtered DI water until use.

Data Analysis

Shows the results of the quadruplicate trials for each biological tested for this study. All results indicate the calculated viable bioaerosol concentration both upstream and downstream of the Clean Air System and the Net LOG reduction provided by the system.

All trials show individual and group average +/- standard deviations for Net LOG reduction on a per challenge organism basis.

Results by Organism

Staphylococcus epidermidis

The *S. epidermidis* are a gram positive, aerobic bacteria that grow in trypticase soy at 37°C and were chosen to act as surrogates for *Staph aurus.* Infections from *S. aurus* are a leading cause of hospital-acquired infections and are linked to 50,000 deaths in the USA per year.

Staph cultures were initiated the day prior to their testing, and grew to a concentration greater than $1e^{10}$ cfu/ml. After nebulization, initial concentrations of *Staph* averaged $2.6e^{5}$ cfu/L of atmosphere inside the testing chamber. The control trial experienced only a 0.62 LOG reduction of *Staph* after 90 minutes of sample collections using the AGI-30 impingers.

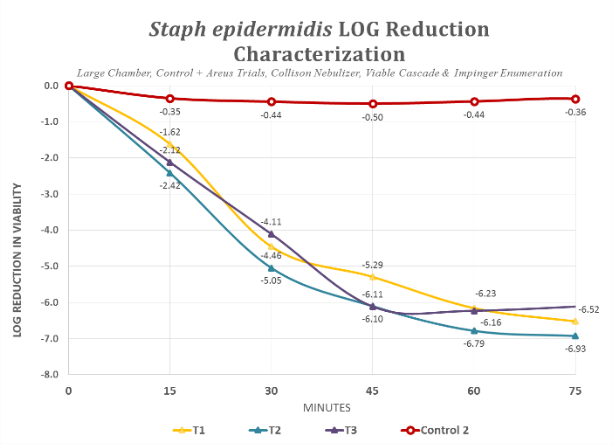

Figure 5: LOG Reduction of *S. epidermidis* in control and Medical Guardian test trials.

The Medical Guardian trials saw a drastic increase in reduction with the unit activated. In 15 minutes, over the three test trials, an average of only 1.183% of the viable *Staph* still remained inside the testing chamber. **Figure** 5 shows the LOG reduction of *Staph* for the control and test trials over the 90 minute testing periods. At 60 minutes there was an average net LOG reduction of *Staph* of 5.95 +/- 0.34 with a limit of detection of 8.15. **Figure 6** shows the LOG reductions for each *Staph* trial and their average +/- standard deviation.

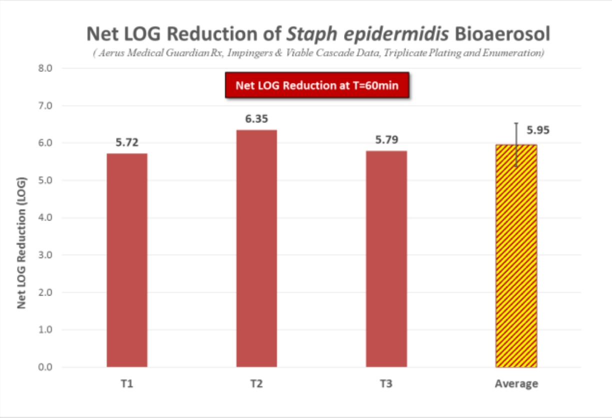

Figure 6 Net LOG Reduction of *Staphylococcus epidermidis*

Erwinia herbicola

The black plague surrogate, *Erwinia herbicola,* was the second and final vegetative bacterium tested in this study. The gram-negative bacterium were grown overnight to an average concentration of $8.4e^{9}$ cfu/ml,

and after nebulization had an average concentration of 1.9e^5 cfu/L of atmosphere inside the testing chamber.

The *Erwinia* control trial saw a 0.70 LOG reduction after 90 minutes of sample collection. After 15 minutes of being activated, the Medical Guardian removed an average of 99.992% of the viable *Erwinia* from the chamber atmosphere. The average net LOG reduction of *Erwinia* by the Medical Guardian at 75 minutes was 5.36 +/- 0.37 with a limits of detection of 7.64.

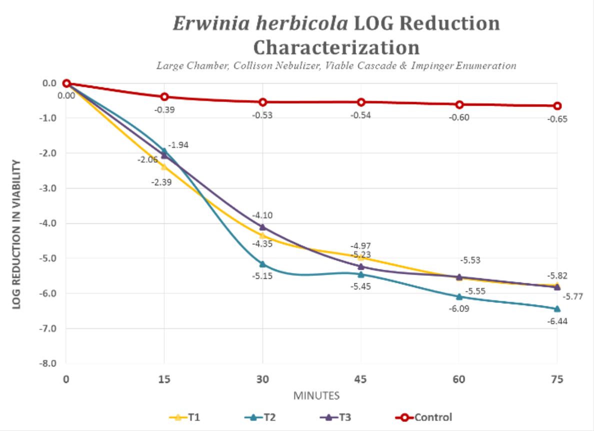

Figure 7: LOG Reduction of *Erwinia herbicola* in control and Medical Guardian test trials.

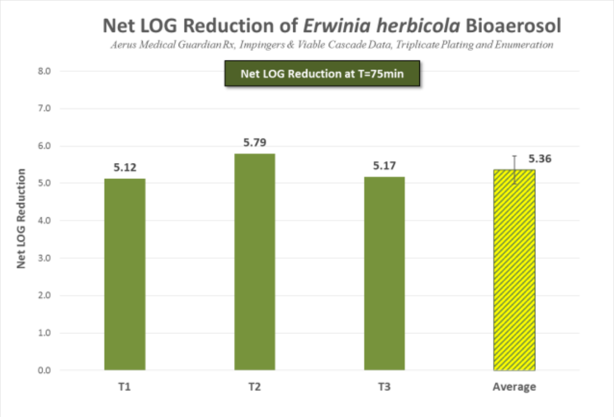

Figure 8: Net LOG *Reduction of Erwinia herbicola.*

MS2 Virus

The bacteriophage MS2 is a single stranded RNA virus that often serves as a surrogate for noroviruses in studies of disease transmission. MS2 infects *E. coli*, which was used as its mechanism of reproduction for this study. Average concentrations of MS2 reached 5.4e^9 pfu/mL in culture pre testing, and chamber concentrations after nebulization averaged 8.1e^5 pfu/L of atmosphere.

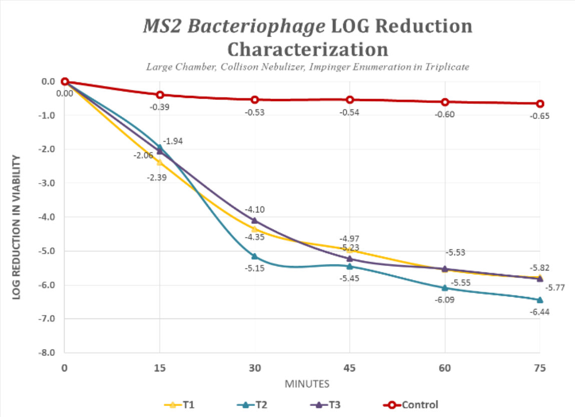

Figure 9: LOG Reduction of MS2 bacteriophage in control and Medical Guardian test trials.

The single control trial saw a slightly increased reduction in viable organisms overtime compared with the vegetative bacterium, reaching a LOG reduction of 0.94 in 90 minutes. With the Medical Guardian activated, an average of 99.999% of the virus had been removed from the chambers atmosphere in 15 minutes. At 60 minutes, there was an average net LOG reduction of MS2 of 5.58 +/- 0.43 with a limits of detection of 6.37. The Net LOG reduction is considered a minimum value because a single pfu was artificially added at 60 minutes in order to show detection limits. There were in reality, no observed viable growth collected at 60 minutes. However, if zeros were to be marked for the plates the logarithmic math does not calculate.

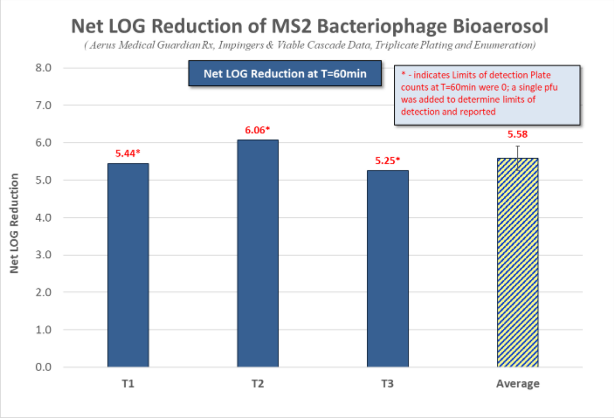

Figure 10: Net LOG Reduction of MS2 bacteriophage

***Bacillus globigii* endospores**

Endospores are an extremely resilient structure formed by some bacteria under unfavorable conditions that are resistant to UV radiation, desiccation, chemical disinfectants and severe temperatures. The *Bacillus*

globigii spores tested served as surrogates for a similar species that causes Anthrax.

Unlike the vegetative bacterium and viruses, the spores did not need to be cultured before testing. Large quantities of spores stored in alcohol can be purchased and are ready for immediate testing. Pre nebulization stock of *B. globigii* was at a concentration of $2.7e^9$ cfu/mL, and chamber concentration post nebulization had an average concentration of $7.95e^5$ cfu/L.

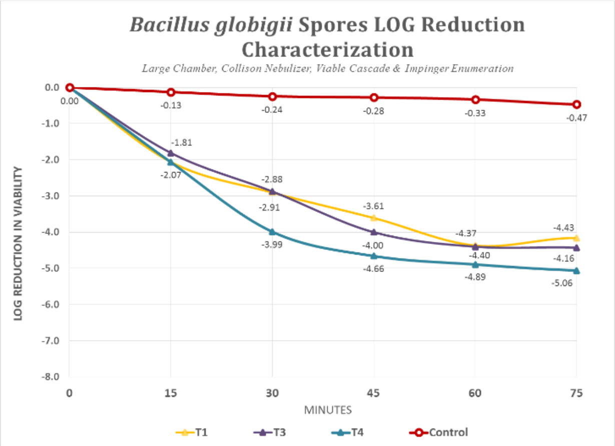

Figure 11: LOG Reduction of *Bacillus globigii* endospores in control and Medical Guardian test trials.

With the Medical Guardian off, the control trial saw a LOG reduction after 90 minutes of 0.49. After 15 minutes with the Medical Guardian activated during the test trials, 1.09% of the spores remained in the chambers atmosphere. After 90 minutes there was a net LOG reduction of 4.23 +/- 0.31 with a limits of detection of 5.70.

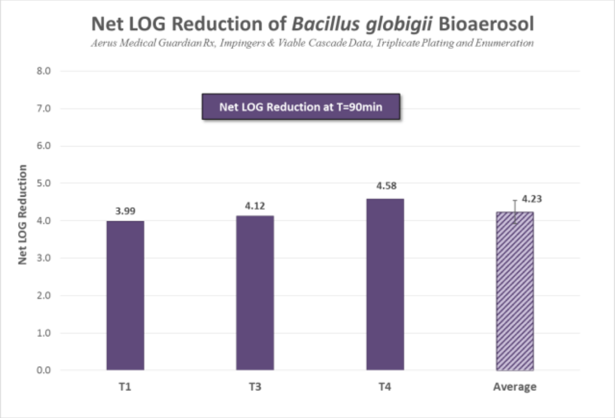

Figure 12: Net LOG Reduction of *Bacillus globigii* endospores

Aspergillus niger Mold Spores

Aspergillus niger, the second spore based organism tested is a fungus rather than bacterial species. It is known for causing black mold disease in fruits and vegetables and is closely related to *Aspergillus fumigatus,* the most common airborne fungal pathogen. Like *B. globigii*, the *A. niger* spores did not require cultivation, but were commercially acquired ready for testing.

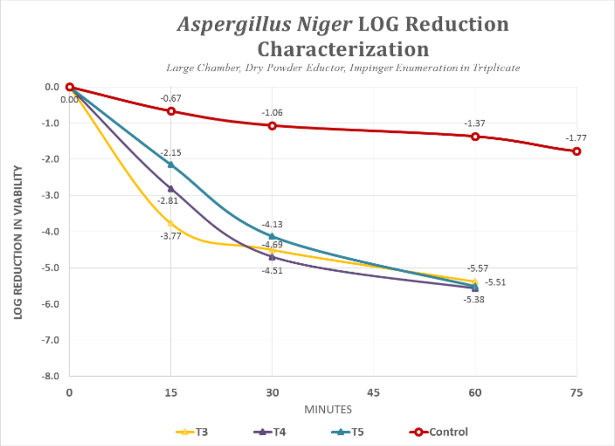

Figure 13: LOG Reduction of *Aspergillus niger* spores in control and Medical Guardian test trials.

Concentrations of *A. niger* inside the chamber after nebulization was less than previous organisms, with an average of $2.61e^4$ cfu/L. The control trial revealed a LOG reduction after 90 minutes of 1.77, which was higher than had been observed previously. With the Medical Guardian activated for 15 minutes during testing trials, an average of only 0.29% of *A. niger* remained inside the chamber. After 60 minutes there was an average net LOG reduction of 4.12 +/- 0.10 with a limits of detection of 5.59.

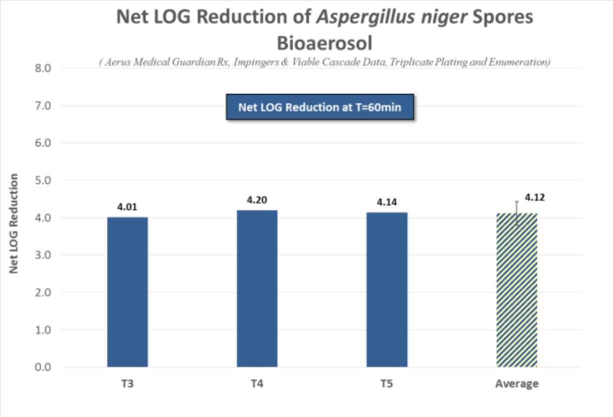

Figure 14: Net LOG Reduction of *Aspergillus niger* spores.

Phi X174 Virus

The Phi X174 bacteriophage testing was planned immediately following MS2 testing, however obstacles arose with culturing the virus to desirable concentrations. The testing was pushed back to the end of the study to give time to develop slight alterations to the growth protocols. Like MS2, Phi X174 infects *E. coli,* however it is a DNA virus with a small genome.

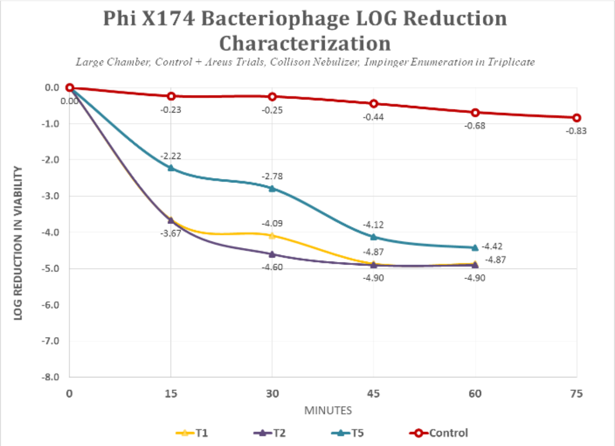

Figure 15: LOG Reduction of Phi X174 bacteriophage in control and Medical Guardian test trials.

Pre-testing cultures of Phi X174 had an average concentration of $4.4e^{8}$ pfu/mL, which was significantly lower than any other organisms tested. Once nebulized, the concentration of the atmosphere inside the testing chamber averaged $7.63e^{3}$ pfu/L. There was a LOG reduction of Phi X174 of 0.89 at 90 minutes during the control trial, and an average net LOG reduction of 4.05 +/- 0.27 with limits of detection of 4.73 during testing trials. There were no plaques observed beyond 30 minutes and a single pfu was added to determine detection limits.

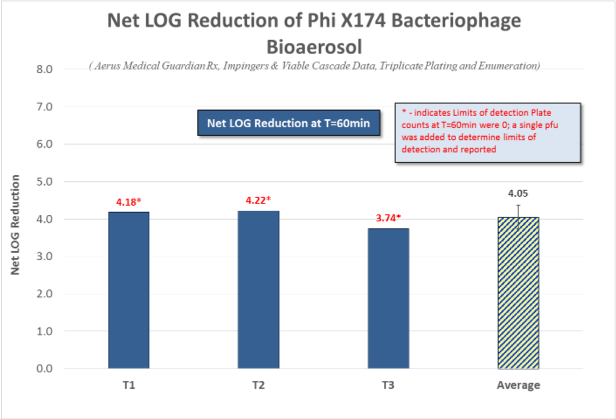

Figure 16: Net LOG Reduction of Phi X174 bacteriophage.

Aerus Medical Guardian Bioaerosol Challenge Summary					Net LOG Reduction Results						Limits of Detection
	Challenge Organism	Surrogate for	ATCC #	Gram	Time (min)	T1	T2	T3	Avg Net LOG Reduction	Standard Deviation	
1	*Staphylococcus epidermidis*	Methicillin Resistant *Staph aureus* (MRSA)	12228	pos	60	5.72	6.35	5.79	5.95	0.34	8.15
2	*Erwinia herbicola*	*Yersinia pestis* (Black Plague)	27155	neg	75	5.12	5.79	5.17	5.36	0.37	7.64
3	MS2 virus	Influenza, Norovirus	15597-B1	NA	60	5.44	6.06	5.25	5.58	0.43	6.37
4	Phi X174 virus	HCV, HBV, HIV	13706-B1	NA	60	4.18	4.22	3.74	4.05	0.27	4.73
5	*Bacillus globigii* endospore	*C. difficile* (spore) & Anthrax (spore)	16404	pos	90	3.99	4.12	4.58	4.23	0.31	5.70
6	*Aspergillis niger* mold spore	Black Mold	13835	NA	60	4.01	4.20	4.14	4.12	0.10	5.59

*Red text = at detection limits, Calculated LOG Reduction values represent a minimum

Table 3: *Net LOG reduction summary for the Medical Guardian air system.*

Summary of Results

Overall, the Medical Guardian's showed an average Net LOG reduction for all organisms tested of 4.80 +/- 0.74. The unit was more effective at removing vegetative bacterium from the testing chambers atmosphere than either the viruses or spores, with an average net LOG reduction for just the two vegetative bacteria of 5.41 +/- 0.07. However testing of both viruses were below detectable limits due there being zero plaques observed on plates after a certain point, meaning that the net LOG reduction for those organisms only represents a minimum value. Spores are known to be tough and highly resilient, and therefore it is not unexpected that their net LOG reductions are not quite as high as the other organisms.

The **Figure 17** below shows each average net LOG reduction for the organisms tested +/- their standard deviations. **Table 3** shows a final summary table of the net LOG reduction results for each organism tested in this study.

The Medical Guardian had an overall Clean Air Delivery Rate of 94.63 cubic feet per minute (cfm), which is explained in detail in **Appendix A.**

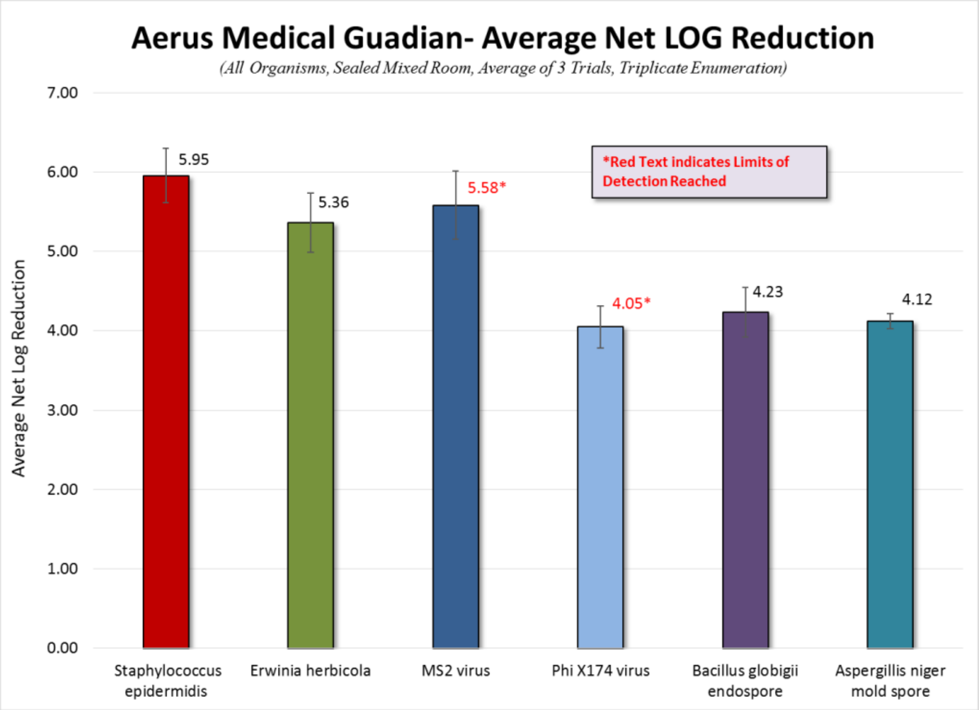

Figure 17: *Average net LOG reduction for each bioaerosol organism tested, average +/- standard deviation for the triplicate trials.*

References

T. Reponen, K. Willeke, V. Ulevicius et al. *Techniques of Dispersion of Microorganisms in Air.* Aerosol Science and TechnoLOGy. 27: 1997. pp. 405-421.

Ding and Wing. *Effects of Sampling Time on the Total Recovery rate of AGI-30 Impingers for E. coli*. Aerosol and Air Quality Research, Vol. 1, No. 1, 2001, pp. 31-36.

J.F. Heildelberg et al. *Effects of Aerosolization on Culturabilty and Viability of Gram-Negative Bacteria*. Applied and Environmental Microbiology. Sept 1997, p 3585-3588.

P Hyman et al. *Practical Methods for Determining Phage Growth Parameters*. Bacteriophages: Methods and Protocols, Vol. 1*: Isolation, Characterization and Interactions.* vol. 501. 2009. pp. 175-201.

Analytical Testing Facility

Aerosol Research and Engineering Labs, Inc.
15320 S. Cornice Street
Olathe, KS 66062

Study Director

Jamie Balarashti
Aerosol Research and Engineering Laboratories

GLP Statement

We, the undersigned, herby certify that the work described herein was conducted by Aerosol Research and Engineering Laboratories in compliance with FDA Good Laboratory Practices (GLP) as defined in 21 CFR, Part 58.

Study Director:

Jamie D. Balarashti
Study Director
ARE Labs, Inc.

3/1/2019
Date

Principal Investigator:

Dan J. Merchant
Principal Investigator
ARE Labs, Inc.

3/1/2019
Date
