# Supplementary material for: Evaluate the safety of a novel photohydrolysis technology used to clean and disinfect indoor air: A murine study
Source: PLoS One. 2024 Oct 9;19(10):e0307031. doi: 10.1371/journal.pone.0307031 (PMC11463749; doi:10.1371/journal.pone.0307031)
Supplement: S7 File — (PDF) [file pone.0307031.s007.pdf]

Medical-Surgical Intensive Care Unit (MSICU)
Case Study

Overview

A prospective study was performed in a suburban Medical-Surgical Intensive Care Unit (MSICU) near New Orleans, Louisiana.

Study Objectives

To examine the effect of a continuous disinfection technology on the microbial burden on surfaces and in the air during the COVID-19 pandemic. Secondary aims included reducing the number of locations found to have greater than 500 Colony Forming Units (CFUs) on a 100cm^2 surface area and reducing the incidence of clinically relevant pathogens, such as Methicillin-Resistant *Staphylococcus aureus* (MRSA).

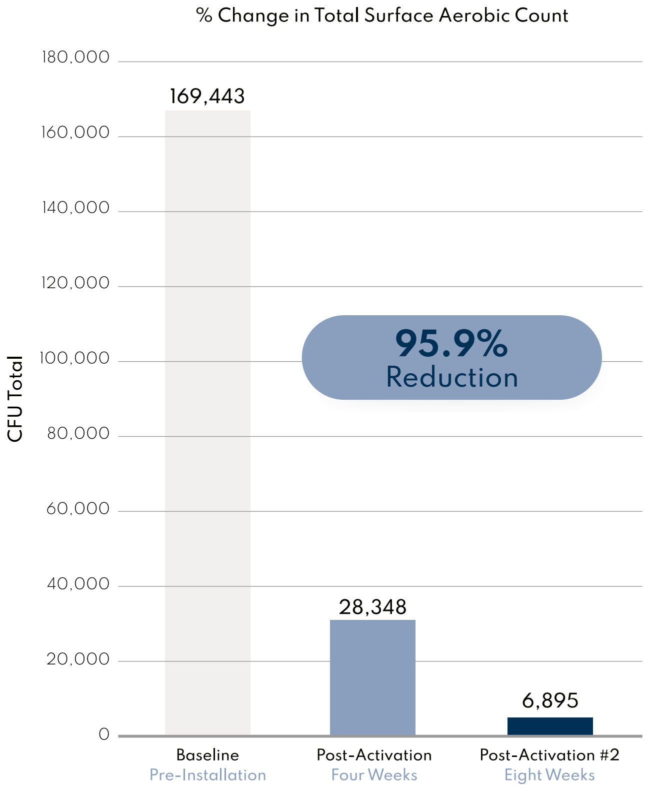

Summary

A baseline sample of 50 environmental cultures and ten air cultures were taken throughout the MSICU, including in patient rooms, nurses' stations, and the physician work area, and were tested for microbial contamination and the presence of Methicillin-Resistant *Staphylococcus aureus* (MRSA). The continuous disinfection technology was then installed into the Heating, Ventilation, and Air Conditioning (HVAC) system and provided a continuous low-level of oxidizing molecules for the entire two-month study period. The facility's normal cleaning and disinfection protocols were followed during this time, and microbial testing occurred every four weeks.

Results

The average CFU count, excluding floors, dropped a statistically significant 95.9% (3,458 average CFU to 140 average CFU) by the second post-activation testing (χ^2, N=49) = 36.954, $p < 0.001$)

There was a 99.8% total decrease in fungal CFUs from baseline to second post-activation test

There was a 99.8% reduction in MRSA CFUs on surfaces from baseline to the second post-activation test

Additionally, there was a 92% decrease in the number of surfaces that had greater than 500 CFUs/100cm^2

Air testing demonstrated a 45% decrease in total bacteria and a 79% decrease in the total fungal CFUs

ActivePure® Medical

This study is still in process but has already demonstrated a statistically significant reduction in microbial burden from baseline.
