# Supplementary material for: Evaluate the safety of a novel photohydrolysis technology used to clean and disinfect indoor air: A murine study
Source: PLoS One. 2024 Oct 9;19(10):e0307031. doi: 10.1371/journal.pone.0307031 (PMC11463749; doi:10.1371/journal.pone.0307031)
Supplement: S8 File — (PDF) [file pone.0307031.s008.pdf]

Surgical ICU (SICU) and Cardiovascular ICU (CVICU)
Case Study

Overview

A prospective quasi-experimental study was performed in a Surgical Intensive Care Units (SICU) and a Cardiovascular Intensive Care Unit (CVICU) in a large urban Canadian hospital.

Study Objectives

To examine the effect of a continuous disinfection technology on the microbial burden on surfaces and in the air.

Summary

Before installing the technology, five surface locations in each ICU were tested for microbial contamination, and air samples were collected in two locations per ICU.

The continuous disinfection technology units were placed in the ICUs and provided a continuous low-level of oxidizing molecules for the entire study period. After seven days, the microbial surface and air testing were repeated.

Results

The SICU data showed a 94% reduction in total bacteria and an 89% reduction in total fungal counts

The CVICU had similar reductions, with a 73% reduction in total bacteria and an 85% reduction in total fungal counts

Additionally, there was an 85% and 64% reduction of total bacteria and fungi in the SICU air

There was a 72% and 68% total bacteria and fungi reduction in the CVICU air

This study showed that the use of a continuously active disinfection technology in two very high acuity ICUs could reduce the microbial burden on surfaces and in the air.

ActivePure® Medical
