# Supplementary material for: Evaluate the safety of a novel photohydrolysis technology used to clean and disinfect indoor air: A murine study
Source: PLoS One. 2024 Oct 9;19(10):e0307031. doi: 10.1371/journal.pone.0307031 (PMC11463749; doi:10.1371/journal.pone.0307031)
Supplement: S9 File — (PDF) [file pone.0307031.s009.pdf]

June 17, 2020

Aerus Medical LLC
℅ Dave Yungvirt
Responsible Third Party Official
Third Party Review Group, LLC
25 Independence Blvd
Warren, New Jersey 07059

Re: K201220
Trade/Device Name: Aerus Medical Guardian, model F170A
Regulation Number: 21 CFR 880.6500
Regulation Name: Medical ultraviolet air purifier
Regulatory Class: Class II
Product Code: FRA
Dated: June 13, 2020
Received: June 16, 2020

Dear Dave Yungvirt:

We have reviewed your Section 510(k) premarket notification of intent to market the device referenced above and have determined the device is substantially equivalent (for the indications for use stated in the enclosure) to legally marketed predicate devices marketed in interstate commerce prior to May 28, 1976, the enactment date of the Medical Device Amendments, or to devices that have been reclassified in accordance with the provisions of the Federal Food, Drug, and Cosmetic Act (Act) that do not require approval of a premarket approval application (PMA). You may, therefore, market the device, subject to the general controls provisions of the Act. Although this letter refers to your product as a device, please be aware that some cleared products may instead be combination products. The 510(k) Premarket Notification Database located at https://www.accessdata.fda.gov/scripts/cdrh/cfdocs/cfpmn/pmn.cfm identifies combination product submissions. The general controls provisions of the Act include requirements for annual registration, listing of devices, good manufacturing practice, labeling, and prohibitions against misbranding and adulteration. Please note: CDRH does not evaluate information related to contract liability warranties. We remind you, however, that device labeling must be truthful and not misleading.

If your device is classified (see above) into either class II (Special Controls) or class III (PMA), it may be subject to additional controls. Existing major regulations affecting your device can be found in the Code of Federal Regulations, Title 21, Parts 800 to 898. In addition, FDA may publish further announcements concerning your device in the Federal Register.

Please be advised that FDA's issuance of a substantial equivalence determination does not mean that FDA has made a determination that your device complies with other requirements of the Act or any Federal statutes and regulations administered by other Federal agencies. You must comply with all the Act's

requirements, including, but not limited to: registration and listing (21 CFR Part 807); labeling (21 CFR Part 801); medical device reporting (reporting of medical device-related adverse events) (21 CFR 803) for devices or postmarketing safety reporting (21 CFR 4, Subpart B) for combination products (see https://www.fda.gov/combination-products/guidance-regulatory-information/postmarketing-safety-reporting-combination-products); good manufacturing practice requirements as set forth in the quality systems (QS) regulation (21 CFR Part 820) for devices or current good manufacturing practices (21 CFR 4, Subpart A) for combination products; and, if applicable, the electronic product radiation control provisions (Sections 531-542 of the Act); 21 CFR 1000-1050.

Also, please note the regulation entitled, "Misbranding by reference to premarket notification" (21 CFR Part 807.97). For questions regarding the reporting of adverse events under the MDR regulation (21 CFR Part 803), please go to https://www.fda.gov/medical-devices/medical-device-safety/medical-device-reporting-mdr-how-report-medical-device-problems.

For comprehensive regulatory information about medical devices and radiation-emitting products, including information about labeling regulations, please see Device Advice (https://www.fda.gov/medical-devices/device-advice-comprehensive-regulatory-assistance) and CDRH Learn (https://www.fda.gov/training-and-continuing-education/cdrh-learn). Additionally, you may contact the Division of Industry and Consumer Education (DICE) to ask a question about a specific regulatory topic. See the DICE website (https://www.fda.gov/medical-devices/device-advice-comprehensive-regulatory-assistance/contact-us-division-industry-and-consumer-education-dice) for more information or contact DICE by email (DICE@fda.hhs.gov) or phone (1-800-638-2041 or 301-796-7100).

Sincerely,

Clarence W. Murray III -S

For Elizabeth Claverie, M.S.
Assistant Director
DHT4B: Division of Infection Control
and Plastic Surgery Devices
OHT4: Office of Surgical
and Infection Control Devices
Office of Product Evaluation and Quality
Center for Devices and Radiological Health

Enclosure

DEPARTMENT OF HEALTH AND HUMAN SERVICES
Food and Drug Administration

Indications for Use

Form Approved: OMB No. 0910-0120
Expiration Date: 06/30/2020
See PRA Statement below.

510(k) Number *(if known)*
K201220

Device Name

Aerus Medical Guardian, model F170A

Indications for Use *(Describe)*
The Aerus Medical Guardian, model F170A is a device intended for medical purposes that is used for the reduction of staphylococcus epidermidis and erwinia herbicola bacteria, MS2 and Phi-X174 viruses and aspergillus niger fungal spores and bacillus globigii bacterial spores from the air in a temperature-controlled professional healthcare environment of 70~71°F, 40~45% RH.

The Aerus Medical Guardian, model F170A demonstrated the reduction of staphylococcus epidermidis and erwinia herbicola bacteria, MS2 and Phi-X174 viruses and aspergillus niger fungal spores and bacillus globigii bacterial spores under the following conditions.

Organism Type	Organism Name	Test Temp/RH	Exposure Time (m)	Avg Log-Reduction
Bacteria	Staphylococcus epidermidis	72°F / 50%	60	5.95
Bacteria	Erwinia herbicola	72°F / 50%	60	5.12
Virus	MS2	72°F / 50%	60	5.58
Virus	Phi-X174	72°F / 50%	60	4.19
Fungal Spore	Aspergillus niger	72°F / 50%	60	4.12
Bacterial Spore	Bacillus globigii	72°F /50%	60	4.22

Type of Use *(Select one or both, as applicable)*

☐ Prescription Use (Part 21 CFR 801 Subpart D)   ☒ Over-The-Counter Use (21 CFR 801 Subpart C)

CONTINUE ON A SEPARATE PAGE IF NEEDED.

This section applies only to requirements of the Paperwork Reduction Act of 1995.

DO NOT SEND YOUR COMPLETED FORM TO THE PRA STAFF EMAIL ADDRESS BELOW.

The burden time for this collection of information is estimated to average 79 hours per response, including the time to review instructions, search existing data sources, gather and maintain the data needed and complete and review the collection of information. Send comments regarding this burden estimate or any other aspect of this information collection, including suggestions for reducing this burden, to:

Department of Health and Human Services
Food and Drug Administration
Office of Chief Information Officer
Paperwork Reduction Act (PRA) Staff
PRAStaff@fda.hhs.gov

"An agency may not conduct or sponsor, and a person is not required to respond to, a collection of information unless it displays a currently valid OMB number."
