# Supplementary material for: Evaluate the safety of a novel photohydrolysis technology used to clean and disinfect indoor air: A murine study
Source: PLoS One. 2024 Oct 9;19(10):e0307031. doi: 10.1371/journal.pone.0307031 (PMC11463749; doi:10.1371/journal.pone.0307031)
Supplement: S10 File — (PDF) [file pone.0307031.s010.pdf]

ASHRAE 241-2023 Standard Testing for the Efficacy of the ActivePure Beyond Guardian Air at Reducing Aerosolized *MS2*

W. Andrew Dexter [a], Richard Ludwick [a], Jamie Balarashti [a]
[a] Aerosol Research and Engineering Laboratories Inc. Olathe KS

Report Info

Submitter:
ActivePure Technologies

Testing Lab:
Aerosol Research and Engineering Laboratories, Inc.
12880 Metcalf Ave
Overland Park, KS 66213

Report History:
ActivePure: Beyond Guardian Air
Aerus: Beyond Guardian Air
Bioaerosol Efficacy
Project Number: 10860.30.1.1
Submitted:

Associated Report:
ActivePure: Beyond Guardian Air
Safety Monitoring
Project Number: 10860.30.1.2
Submitted:

Keywords:
- *ActivePure Beyond Guardian Air*
- *Bioaerosol Efficacy*
- *MS2*
- *ASHRAE 241-2023*
- *AHAM AC-5*

ASHRAE 241-2023 Compliance:
This study was conducted in compliance with ASHRAE 241 and Good Laboratory Practices (GLP) as defined in 21 CFR, Part 58,

Conflict of Interest:
Aerosol Research and Engineering Laboratories, Inc. have no affiliations with, or involvement in any capacity, with ActivePure's financial interests such as membership, employment, stock ownership, or other equity interest.

ABSTRACT

Purpose:

The purpose of this in-vitro study was to measure the efficacy of the ActivePure Beyond Guardian Air and its ability to reduce the bacteriophage MS2 and the gram-positive bacteria *Staphylococcus epidermidis*, per the ASHRAE 241-2023 standard. This device also may be referred to as the Aerus Beyond Guardian Air.

Background:

The ActivePure Beyond Guardian Air is a medical-grade air purifier and surface decontamination device. The Beyond Guardian Air houses a proprietary catalyst coated honeycomb matrix that is struck with 254 nm UV light creating air scrubbing like particulates that work to inactivate various types of pathogens commonly found in an everyday environment. A corresponding filter also aids in the removal of pathogens from the air.

All testing was conducted in a 30m^3 bioaerosol test chamber which housed the Beyond Guardian Air unit. The challenge organism initially selected for this study was the ssRNA bacteriophage MS2. This bacteriophage is a widely utilized and recognized surrogate for more dangerous pathogenic organisms like influenza and SARS-CoV-2. Additional testing was done with *Staphylococcus epidermidis*, a gram-positive bacteria used as a surrogate for MRSA. This study utilizes ASHRAE 241 and AHAM AC-5 testing parameters to determine efficacy. Three separate bioaerosol test trials were performed for the ActivePure Beyond Guardian Air device. While *S. epidermidis* is not a listed organism according to ASHRAE 241, it still provides important information on the efficacy of the device.

Methods:

The ActivePure Beyond Guardian Air was placed at the center of the 30m^3 chamber. MS2 was then aerosolized into the sealed 30m^3 environmental bioaerosol chamber, with the chamber containing the ActivePure Beyond Guardian Air, using a Collison 24-Jet Nebulizer. *MS2* was the microorganism used for all initial aerosol test trials, additional testing was done with *Staphylococcus epidermidis* to provide a better understanding of the CADR seen at various time points throughout the trial. Previously prepared aliquots of *MS2* were used to keep a consistent concentration throughout all testing.

Bioaerosol samples were taken, with impingers, at multiple time points throughout each trial, using ASHRAE 241 and AHAM AC-5 testing parameters, in order to quantify the reduction rate capability of the air purification device. The impinger samples were serially diluted, plated, incubated, and enumerated in triplicate to yield the viable bioaerosol concentration for each sampling time point. Chamber control trial data, or natural decay, was subtracted from the device trial data to yield the net log reduction attributable to the device for each of the bioaerosol challenges.

Results:

The standard ActivePure Beyond Guardian Air device proved to be effective at reducing MS2. ASHRAE 241 focuses heavily on the reporting and calculation of the clean air delivery rate number or CADR. The CADR values were calculated using the net reduction slope multiplied by the chamber volume to get a CADR value. This value was then converted into CFM for a value of 304 CFM for t-0 to t-6 minutes and 193 CFM for t-0 to t-20 minutes. Additionally, *Staphylococcus epidermidis* was also challenged, resulting in a CADR of 340 CFM from t-0 to t-10 and 199 CFM from t-0 to t-60 minute.

Introduction

This study was conducted to evaluate the efficacy of the ActivePure Beyond Guardian Air device for its individual effectiveness at deactivating aerosolized MS2 and *Staphylococcus epidermidis*. The device is specifically designed to be a standalone unit that uses ActivePure technology along with a filter to remove and inactivate various pathogens from the air and surfaces when in operation.

Proprietary and Confidential - Property of ActivePure

On June 24th, 2023, the new ASHRAE 241-2023 guidelines were released to establish a more uniform testing protocol for all air purification devices. This protocol standardized all components of bioaerosol testing for both in duct and standalone devices. This testing protocol established the minimum requirements needed to evaluate all production air purification devices adequately and effectively moving forward.

The ASHRAE standard includes guidelines for proper ventilation, infection risk management, laboratory testing requirements, operation, and maintenance for devices, as well as special requirements needed for residential and health care facilities. With these new guidelines, testing must be done on all air purification devices that are certified as adhering to these ASHRAE 241 standards.

Following these guidelines, the test plan incorporated challenging the ActivePure devices using the ASHRAE 241 and AHAM AC-5 protocols and requirements for a 30 m^3 test chamber. This report will focus on the efficacy of the ActivePure Beyond Guardian Air device.

Study Overview

The effectiveness of the ActivePure Beyond Guardian Air device was evaluated against two separate aerosolized organisms, MS2*,* an ssRNA virus and *Staphylococcus epidermidis* a gram-positive bacterium. This allowed for a reasonable demonstration of the performance of the devices while operating in their intended manner. This study was done in accordance with ASHRAE 241, and AHAM AC-5 testing parameters.

This is one report of two that details the requirements for ASHRAE 241 and AHAM AC-5 testing. This report contains all bioaerosol testing parameters, data, and results, while the other report details the safety information required by ASHRAE 241 and AHAM testing guidelines. A test matrix outlining the testing can be found in **Figure 2**.

Test Device Description

The ActivePure Beyond Guardian Air, **Figure 1**, is a medical-grade air purifier and surface decontamination device. This Beyond Guardian Air houses a proprietary catalyst coated honeycomb matrix that is struck with 254 nm UV light creating air scrubbing like particulates that work to inactivate various types of pathogens commonly found in an everyday environment. A corresponding filter also aids in the removal of these pathogens from the air. This device is designed to be placed into a room in either a hospital, business, or residential home space.

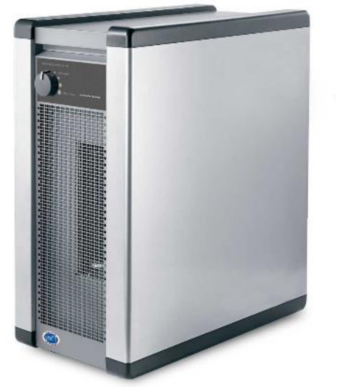

Figure 1: ActivePure Beyond Guardian Air.

Equipment

Bioaerosol Testing Chamber

The test chamber is the main component in bioaerosol testing used for controlled manipulation and testing of microorganisms. It allows for the introduction, sampling, and secure confinement of microorganisms, thus contributing to the precision and reproducibility of testing outcomes. ARE Lab's 30m^3 test chamber adheres to the stringent guidelines in AHAM AC-5 and aligns with both AHAM and ASHRAE 241 criteria.

Bioaerosol Challenge Test Matrix

Trial Number	Test Device	Pathogenic Organism	Surrogate Species Used in Testing	ATCC #	Target Aerosol Particle Size (um)	Challenge Conc. (#/L)	Trial Length (min)	Sampling Time Points (min)	Sampling Equipment	Plating and Enumeration
1<br>2<br>3	Control	*Influenza, Coronaviruses*	*MS2 Bacteriophage (RNA Virus)*	*15597-B1*	<1.0	10^4-10^6	40	0, 1, 2, 3, 4, 5, 6, 7, 8, 12, 16, 20	AGI-30 Impingers	All Samples Plated in Triplicate
4<br>5<br>6	Beyond Guardian Air									
7<br>8<br>9	Control	*Methicillin resistant Staphylococcus aureaus (MRSA)*	*Staphylococcus epidermidis*	*12228*	2.4	10^4-10^6	80	0, 1, 2, 3, 4, 5, 6, 7, 8, 9, 10, 16, 20, 40, 60	AGI-30 Impingers	All Samples Plated in Triplicate
10<br>11<br>12	Beyond Guardian Air									

Figure 2: Test Matrix for Bioaerosol Testing.

Proprietary and Confidential - Property of ActivePure

Structurally, the chamber has dimensions of 30 ± 1.5 cubic meters, or approximately 1060 ft^3, with the width deliberately maintained within 85 to 100% of its length. This dimensional consistency ensures a uniform testing space, which allows for reliable experimentation. Constructed from a non-porous material, the chamber's walls exhibit notable qualities. Beyond its physical attributes, this material emits minimal volatile organic compounds (VOCs), is non-reactive, non-reflective, and has a non-ionizing quenching nature. This creates an environment conducive to reliable and repeatable testing conditions.

Airtight integrity is monitored and controlled, within the chamber achieving a controlled air change rate (ACH) below 0.05, as per the benchmark set by ASTME 741. This characteristic provides the operator with the ability to isolate the testing environment, thus enhancing result reliability.

The chamber is designed to prevent external microbial contamination while maintaining internal atmospheric conditions. These features include an aseptic maintenance system, HEPA filtration, cross-contamination-free item transfer mechanisms, external power control, real-time observation facilitated by multiple viewing windows, and the capability to produce and evenly disperse aerosolized microbes.

Sampling ports, positioned approximately 48 inches from the floor and 12 inches from the walls, ensure optimal sample collection while maintaining prescribed device separation. The chamber's temperature and humidity are maintained, within ASHRAE 241 limits, with a programmable controller.

The incorporation of negative pressure airflow allows for controlled purging, and a HEPA filter adds an additional layer of protection, inhibiting potential contamination. The 30m^3 testing chamber at ARE Labs fulfills both AHSRAE 241 and AHAM AC-5 requirements. **Figure 3** shows the bioaerosol chamber used for all testing in this study. A Magnehelic gauge (Dwyer instruments, Michigan City IN), with a range of -0.5 to 0.5 inches of H_2O, is used to monitor and balance the system pressure during aerosol generation, aerosol purge, and testing cycles. A general flow diagram of the aerosol test system is shown in **Figure 4** below.

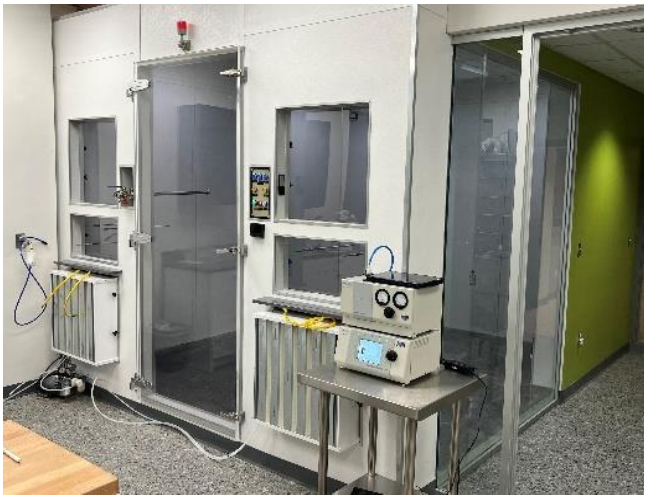

Figure 3: The 30 m^3 bioaerosol testing chamber at ARE Labs adheres to AHAM AC-5 standards and ASHRAE 241 criteria.

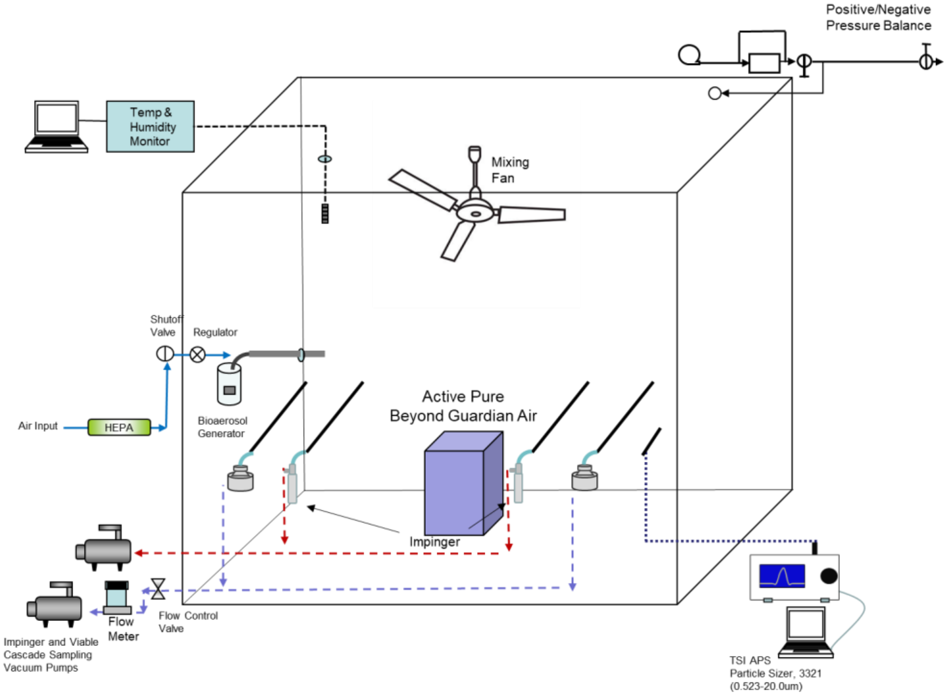

Figure 4: 30m^3 Environmental Test Chamber Flow Diagram. Chamber includes bioaerosol induction, multiple bioaerosol sampling ports, particle size monitoring, internal mixing fans, and temperature and humidity controls. Main system HEPA evacuation system not pictured.

Proprietary and Confidential - Property of ActivePure

Bioaerosol Generation System

As per the AHAM AC-5 requirements, the Collison nebulizers are able to produce 0.05 um to 5 um particles from microbial suspensions using compressed air to generate aerosols. The nebulizer fluid is a mixture of the test microorganism, distilled water, phosphate buffer solution (PBS), and an antifoaming agent. A ceiling fan is used in the chamber to allow for homogenous mixing.

A 24-Jet Collison (BGI Inc. Waltham MA), similar to the one shown in **Figure 5** below, was used during testing to introduce the properly sized particulates into the test chamber. The biologic was mixed with half PBS, half fresh Tryptic Soy Broth (TSB), both made with distilled water and 100uL of antifoam A concentrate. The aerosolization of bioaerosols was driven by dry, filtered house air. A pressure regulator allowed for control of disseminated particle size, use rate, and sheer force generated within the Collison nebulizer.

Prior to testing, the Collison nebulizer flow rate and use rate were checked using an air supply pressure of approximately 40-60 psi, which produced an output volumetric flow rate of 50-80 L/min with a fluid dissemination rate of approximately 1.25 mL/min. The Collison nebulizer was flow characterized using a calibrated TSI model 4040 mass flow meter (TSI Inc., St Paul MN).

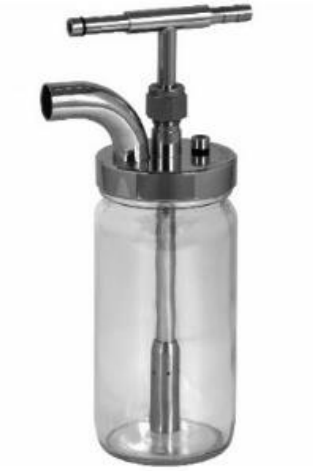

Figure 5. 6-Jet Collison nebulizer. Glass and 304 stainless steel construction, made by BGI Industries.

Bioaerosol Sampling System

Two AGI-30 impingers (Ace Glass Inc. Vineland NJ) were used for bioaerosol collection to determine chamber concentrations. These two AGI-30 Impingers were placed at opposite sides of the chamber in order to better represent the entire room. The mixing fan inside the chamber worked to ensure a homogenous air mixture inside the chamber. A picture of the AGI-30 is shown in **Figure 6** below.

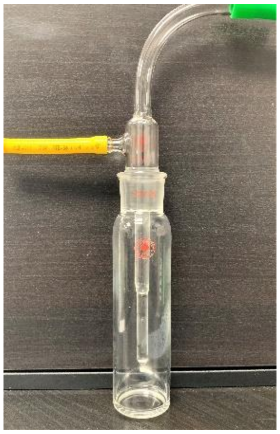

Figure 6: AGI-30 Impinger, Ace Glass Inc. Vineland NJ.

The AGI-30 impinger vacuum source was maintained at a negative pressure of -18 inches of Hg during all characterization and test sampling to assure critical flow conditions. The AGI-30 impingers sample at a rate of 12.5 LPM impinger flows were characterized using a calibrated TSI model 4040 mass flow meter.

Temperature and Humidity Monitor/Controller

The temperature and humidity within the chamber are monitored and controlled with an AC Infinity Controller 69. This controller allows for real-time monitoring and control of the temperature in the 30m^3 bioaerosol chamber used for testing. Temperature and humidity control is essential for the stability of aerosolized micro-organisms during testing.

ASHRAE 241 and AHAM AC-5 both have temperature and humidity requirements for temperature and humidity inside of the bioaerosol chamber during testing. The required range for humidity is 50% $\pm$ 10% while the temperature range is 73°F + 5° (23°C + 3°C). A picture of the controller is shown in **Figure 7** below.

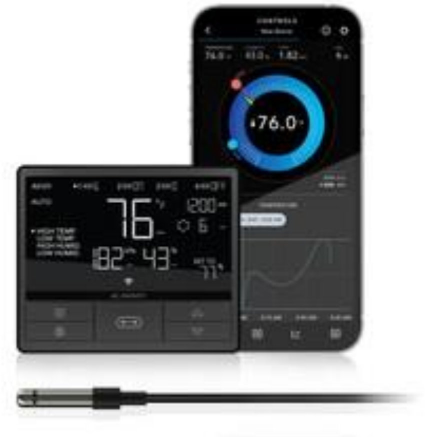

Figure 7: AC Infinity Controller 69 Temperature and Humidity Controller.

Ion Monitor

The COM ion meter, **Figure 8** below, measures ion concentrations in real time and was used during testing to ensure the ion concentrations were consistent inside the chamber. The ion meter measures ions using the Gerdien capacitor method and can detect positive and negative ions down to 10 per cubic centimeter.

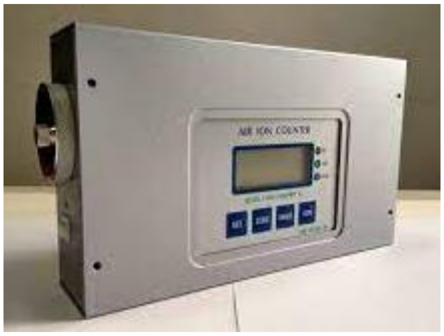

Figure 8: COM 3200Pro II ion meter used for ion measurements of the PA663 ionizer.

TSI Aerodynamic Particle Sizer (APS)

A TSI model 3321 Aerodynamic Particle Sizer (APS) (TSI Inc., Shoreview, MN) was used to measure aerosol concentrations and the particle size distribution within the chamber during the test trials. The APS provided real-time aerodynamic particle characterization with a size range from 0.54-20.0 µm with 52 size bins of resolution. Sampling is continuous with a data export interval of 1 second. The APS has a continuous flow rate of 5 liters per minute (LPM). A picture of the APS is shown in **Figure 9** below.

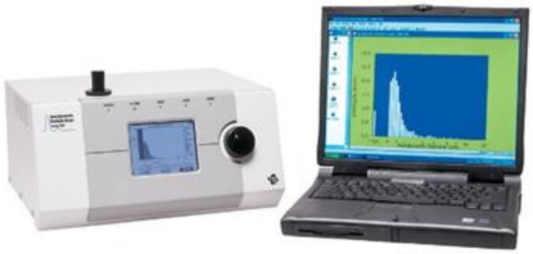

Figure 9. TSI Aerodynamic Particle Sizer (APS) model 3321 used to measure total particle concentration and particle size distribution of the challenge bioaerosol. It has a range of 0.54-20.0 µm aerodynamic diameter, with 1 particle/L detection limits.

Chamber Validation

Validating a bioaerosol chamber is a crucial process to ensure its accuracy and reliability in maintaining controlled experiments. This involves thorough assessments to confirm that the chamber met the strict standards for conducting bioaerosol studies. Factors such as chamber homogeneity, ionization assessment, air exchange rates, and control stability are rigorously tested to ensure consistent and accurate results. Validation assures researchers that the chamber functions properly, enabling them to conduct reliable bioaerosol studies that contribute to informed decision-making in areas like indoor air quality and infectious disease research.

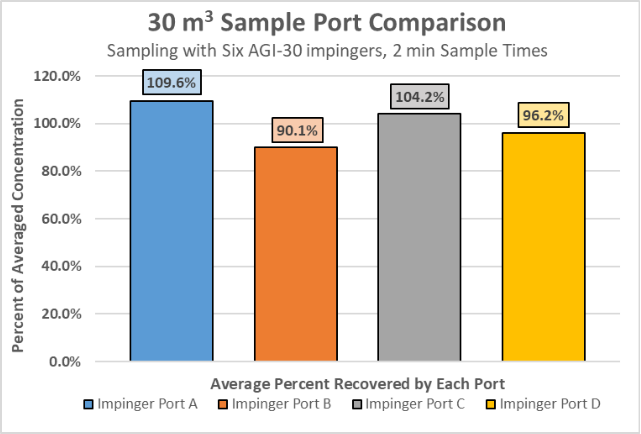

Figure 10: Impinger port-to-port comparison. Percent averages are calculated by taking the count for each port divided by the average plate count for the four ports.

Homogeneity

One key component of the chamber validation process is the bioaerosol homogeneity test. This test validates the homogeneity of the chamber, making sure that the atmosphere within the chamber is well mixed.

Six AGI-30 impingers were used for this chamber validation. The impingers were systematically rotated through all four impinger ports to generate a matrix of impinger tests against all ports. Each port was tested with each impinger a minimum of two times during this validation.

These impinger samples were plated in triplicate by two technicians to reduce plating discrepancies. Each set of plate counts generated by each technician were compared to one another and a port-to-port comparison was created. This showed that each port of the 30m^3 chamber produced a similar result to one another validating the chamber homogeneity during trials. A graphical representation of the average measured for each port is shown in **Figure 10**.

Ionization Validation

To measure the baseline ion concentration, present in the sealed 30 m^3 chamber over 4 hours, a COM 3200 Pro II ion meter was used. The chamber had an average net ion concentration of -143.39 +/- 55.64 ions per cubic centimeter. Testing shows that the net ion concentration is essentially neutral in regard to the charge within the chamber. See ion data graph from trial in **Figure 11**. The total production of ions naturally occurring in the chamber is nominal.

Proprietary and Confidential - Property of ActivePure

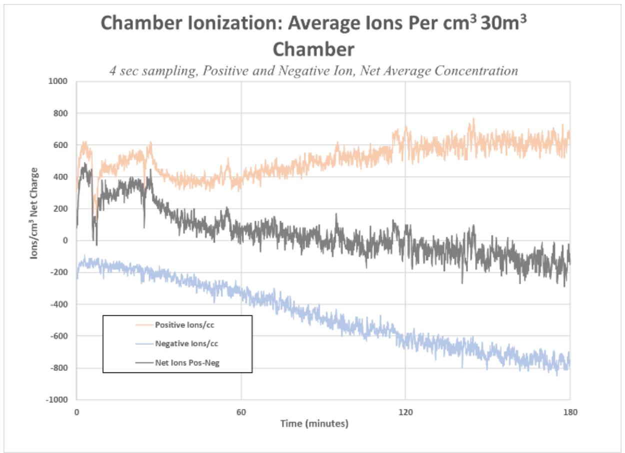

Figure 11. Total baseline level of ions present in the 30m³ chamber.

Chamber Environmental Controls

Chamber controls involve assessing the natural decay rate of the microbes within the test chamber over a defined time period without the air cleaner operation. The duration of this time period aligns with the intended operational testing time of the air cleaner, with multiple sampling points set at intervals of twenty minutes to establish a robust natural decay curve.

Microbes are collected using an impinger filled with phosphate-buffered saline (PBS) solution with 0.005% of the surfactant tween 80, ensuring a representative and homogeneous sample. The sampling rate and volume are precisely defined. If necessary, multiple impingers can be employed in series to enhance collection efficiency.

The samples collected in the impingers are then carefully processed through serial dilution, plating, and enumeration in triplicate (see plating and enumeration section for more information). This meticulous analysis provides viable bioaerosol concentrations at each sampling point and contributes to accurate data interpretation.

For increased stability of bioaerosols, the relative humidity inside the chamber was kept at 50% +/- 10% using a PID humidity controller in combination with an ultra-sonic humidifier to nebulize filtered DI water. Temperature controls maintain chamber trial conditions at typical ambient conditions of 73°F +/- 5°F.

These control tests implement the ANSI/AHMA AC-5 2022 guidelines, ensuring a thorough and precise assessment of air cleaner performance in reducing airborne microbes. The methodical approach, from preparation to measurement and analysis, underscores the importance of consistent and accurate testing procedures.

Testing

Air Cleaner Efficacy Evaluation Procedure

The process of evaluating the efficacy of air cleaners in reducing airborne microbial concentrations is similar to control tests, but the test chamber contains the air cleaner being tested. A suspension of test microbes is nebulized into the chamber air, and an initial measurement of the microbial concentration is taken before activating the air cleaner.

Once the baseline is set, the air cleaner is activated, with the operation time varying according to the specific characteristics of the unit. See **Figure 12**, on the bottom of this page, for an example sampling timeline. For air cleaners with higher Clean Air Delivery Rates (CADR), the operation time could be as brief as 10 minutes, while those with lower CADR might necessitate up to 60 minutes of operation. During the air cleaner's operation, air samples are collected from the chamber at 1-minute intervals over a 20-minute duration. These samples are pivotal in assessing the air cleaner's effectiveness in reducing the microbial concentration. Depending on the capabilities of the air cleaner, supplementary samples can be obtained in 30 and 45 minutes, ensuring a minimum of five valid sampling points.

The collected air samples undergo the following procedure: Serial dilution of the samples is followed by plating, and the viable bioaerosols are enumerated (see plating and enumeration section for more information regarding plating). This analysis yields the microbial concentration at each time point, providing a quantifiable measure of the air cleaner's performance. It's worth noting that, in cases where the microbial concentration becomes exceedingly low, an extension of the sampling duration beyond the originally planned 2-minute sampling may be implemented, although this adjustment should be considered for its potential mathematical implications.

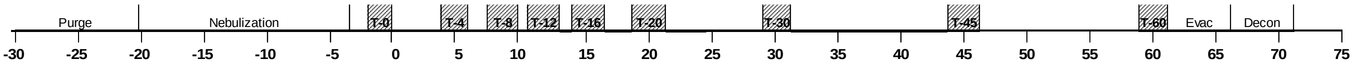

Figure 12: Standard ASHRAE 241 Sampling Times for a 1 Hour Trial. While these were the standard ASHRAE sampling times, these trials were done sampling every minute up to 10 minutes and then carried out for either 20 minutes or an hour.

For air cleaners with exceptionally high CADR ratings, an alternative sampling approach is recommended. This entails obtaining air samples every minute over relevant test period during the air cleaner's operation. Additional sampling points can then be incorporated at various intervals, extending out to 60 minutes.

In adhering to the ASHRAE 241/AHAM protocol, the real-world efficacy of air cleaners across varying operating conditions and CADR levels can be established, thus producing more accurate conclusions regarding indoor air quality management.

Bioaerosol Challenge Particle Size Testing

Bioaerosol challenge particle size distributions were measured with a TSI Aerodynamic Particle Sizer model 3321 (APS) for all challenge species. The particle size distribution was taken shortly after aerosolization for each species via sampling through a sample probe into the test chamber. The APS has a dynamic measurement range of 0.54 to 20.0 µm and was programmed to take consecutive real-time one-minute aerosol samples. Data was logged in real-time to an Acer laptop computer, regressed, and plotted. A graphical representation of MS2 Particle Size Distribution can be found in **Figure 13** along with the *Staphylococcus epidermidis* in **Figure 14**.

Species Selection

Due to safety concerns for bioaerosol testing, organism selection was based on Biological Safety Level 1 (BSL1) species which serve as surrogates for more dangerous pathogens. The ASHRAE 241/AHAM AC-5 guidelines for biological species selection require the use of MS2. MS2, is a ssRNA virus and is very commonly used for bioaerosol testing given its small size and hearty resilience to aerosolization and other disinfecting processes.

In addition to the use of MS2 as the challenge organism, a triplet set of tests were conducted with Methicillin Resistant *Staphylococcus epidermidis* (MRSE) (ATCC 12228). *Staphylococcus epidermidis* is a gram-positive bacterium and BSL1 simulant for a wider range of medically significant pathogens including Methicillin Resistant *Staphylococcus aureus* (MRSA). While this organism is not officially approved by the ASHRAE 241 guidance document as an accepted challenge organism, there is still valuable information that can be gained by challenging the Beyond Guardian Air device with other organisms. This information will be supplemental to the MS2 results which are the major focus of this report and of the AHRAE 241 guidance document as a whole.

Vegetative Cells Culture & Preparation

Pure strain seed stocks were purchased from ATCC (American Type Culture Collection, Manassas VA). For ATCC reference number see Figure 8. Working stock cultures were prepared using aseptic techniques in a class 2 biological safety cabinet and followed standard preparation methodologies. Approximately 250mL of biological stock was prepared in tryptic soy liquid broth media, and incubated for 24-48 hours with oxygen infusion (1cc/min) at 37°C. Biological stock concentrations were approximately 1 x 10^{10} cfu/ml. Stock cultures were centrifuged for 10 minutes at 3000 rpm in an LD-3 centrifuge in sterile 15mL conical tubes, growth media was removed, and the cells re-suspended in sterile PBS buffer for aerosolization. Aliquots of these suspensions were enumerated on tryptic soy agar plates (Hardy Diagnostics, Cincinnati OH) for viable counts and stock concentration calculation. For each organism, test working stocks were grown in sufficient volume to satisfy use quantities for all tests conducted using the same culture stock material.

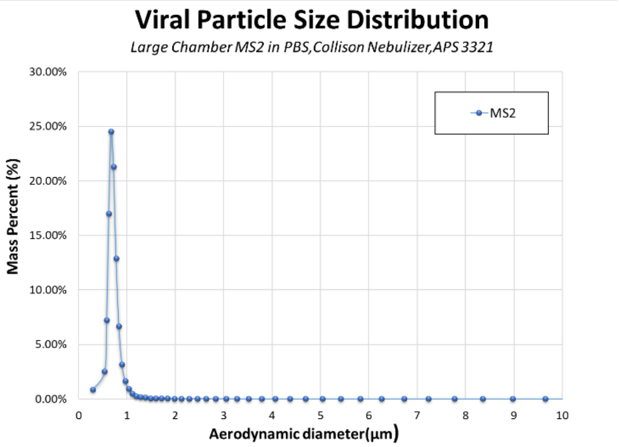

Figure 13: Aerodynamic Particle Size Distribution of the RNA virus MS2 in the test chamber. The MMAD for this viral species averaged approximately 0.7 µm.

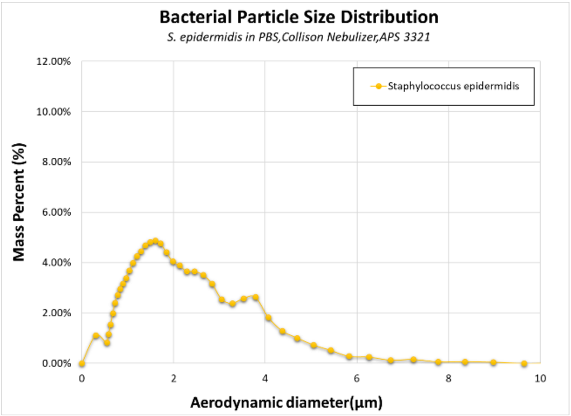

Figure 14: Aerodynamic Particle Size Distribution of *S. epidermidis*. The MMAD for each species was approximately 1-3 µm.

Plating and Enumeration

Impinger and stock biological cultures were serially diluted and plated in triplicate. (Multiple drop samples for

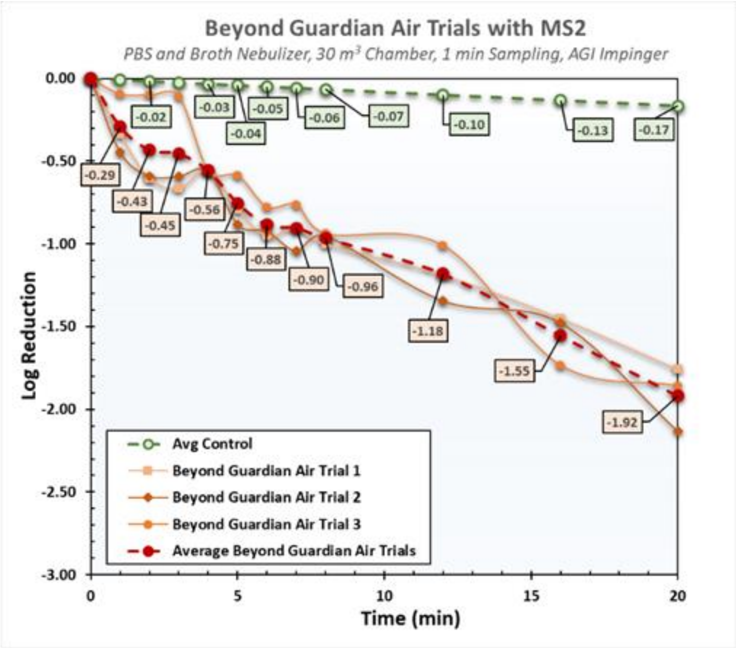

Figure 15: Log Reduction of Aerosolized *MS2* by the Beyond Guardian Air.

each dilution) using a standard drop plate technique onto tryptic soy agar plates.

The drop plate technique is a widely utilized method in microbiology for determining bacterial or viral concentrations in liquid samples. In this technique, known volumes of the liquid sample are serially diluted, and each dilution is carefully dispensed onto solid agar plates. These plates provide a nutrient-rich environment that supports bacterial growth. Once the drops are evenly spread across the surface, the plates are incubated for 24-48 hours, depending on the species, then enumerated and recorded. If using a virus for testing the host organism is added to each tube to allow for viral replication and plaque formation.

The number of colonies or plaques that form on the plates is counted and used to calculate the original bacterial concentration in the liquid sample. The drop plate technique offers a practical and straightforward approach for quantifying bacterial populations, making it a fundamental tool in various research, clinical, and industrial settings for assessing microbial abundance and studying bacterial or viral growth dynamics.

Post-Testing Decontamination and Prep

After the completion of each testing session, a series of post-test actions were carried out to ensure the integrity and cleanliness of the testing environment. The interior of the test chamber underwent decontamination using a UV-C lamp or an appropriate disinfectant solution, such as 70% ethanol, bleach, or vaporous hydrogen peroxide (35%) to ensure the elimination of any residual bioaerosols in accordance with ANSI/AHAM AC-5-2022 guidelines (Section 5.1.14).

The chamber underwent a minimum of twenty minutes of air flow evacuation/purging to restore baseline particle concentration levels, as assessed by the Aerosol Particle Spectrometer (APS). Special care was taken to ensure the thorough removal of any contaminants, with an emphasis on preventing residue buildup on surfaces and in the air. Adequate air exchanges were employed to facilitate the decontamination process, and this step was particularly rigorous when transitioning between different test microbes to mitigate cross-contamination risks.

Data Analysis

Results from the control trials were graphed and plotted to show natural viability loss over time in the chamber. These control trials served as the basis for determining the reduction of both ActivePure Beyond Guardian Air devices over an hour trial above the natural losses from the control runs. The control and trials are plotted showing log reduction in viable bioaerosol for MS2. All data is normalized with time zero enumerated concentrations. Subsequent samples are normalized and plotted to show the loss of viability over time. CADR values were calculated using the graphical method shown in Figure 19 on page 10.

Proprietary and Confidential - Property of ActivePure

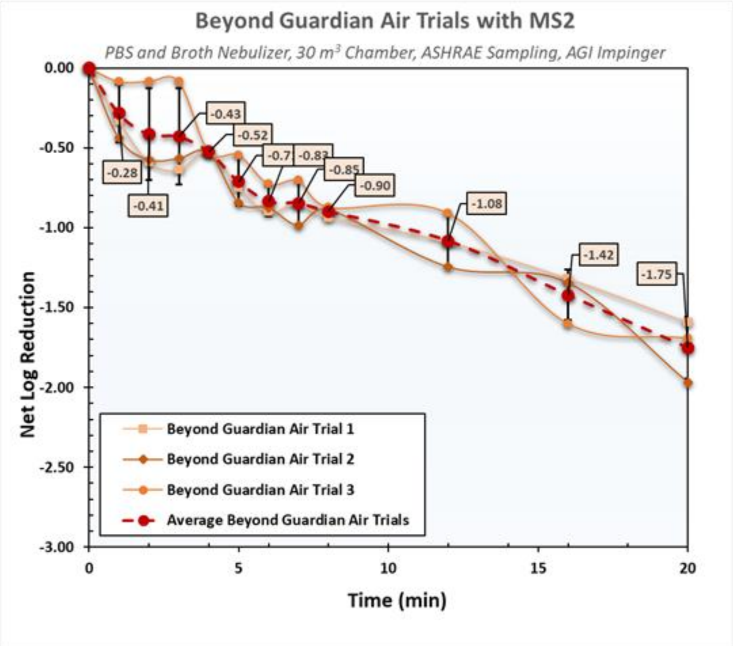

Figure 16: Net log Reduction of Aerosolized *MS2* by the Beyond Guardian Air.

Active Pure Beyond Guardian Air Summary

Bioaerosol Type	Species (description)	Trial Name	Reduction Type	Trial Time (minutes)										
				1	2	3	4	5	6	7	8	12	16	20
Virus	*MS2* (RNA Virus)	*Beyond Guardian Air Trial 1*	*Net Log Reduction*	-0.33	-0.59	-0.64	-0.52	-0.75	-0.90	-0.85	-0.94	-1.09	-1.32	-1.59
			Net % Reduction	53.1468%	74.1833%	76.9727%	69.8198%	82.2247%	87.4556%	85.7918%	88.4132%	91.8677%	95.1873%	97.4473%
Virus	*MS2* (RNA Virus)	*Beyond Guardian Air Trial 2*	*Net Log Reduction*	-0.44	-0.57	-0.56	-0.53	-0.84	-0.87	-0.99	-0.88	-1.24	-1.34	-1.97
			Net % Reduction	63.5938%	73.2797%	72.7620%	70.1775%	85.5861%	86.6426%	89.6517%	86.9528%	94.3048%	95.4687%	98.9201%
Virus	*MS2* (RNA Virus)	*Beyond Guardian Air Trial 3*	*Net Log Reduction*	-0.08	-0.08	-0.08	-0.52	-0.54	-0.73	-0.70	-0.87	-0.91	-1.60	-1.69
			Net % Reduction	17.7450%	17.3492%	17.5795%	70.1272%	71.4516%	81.2457%	80.2231%	86.5600%	87.6646%	97.4928%	97.9696%
All Trial Averages +/- St. Dev.			***Net Log Reduction***	***-0.28 +/- 0.18***	***-0.41 +/- 0.29***	***-0.43 +/- 0.3***	***-0.52 +/- 0***	***-0.71 +/- 0.15***	***-0.83 +/- 0.09***	***-0.85 +/- 0.14***	***-0.9 +/- 0.03***	***-1.08 +/- 0.17***	***-1.42 +/- 0.16***	***-1.75 +/- 0.19***
			Net % Reduction	*44.829% +/- 24.03%*	*54.937% +/- 32.555%*	*55.771% +/- 33.142%*	*70.042% +/- 0.194%*	*79.754% +/- 7.384%*	*85.1146% +/- 3.3752%*	*85.2222% +/- 4.74%*	*87.3086% +/- 0.9765%*	*91.279% +/- 3.359%*	*96.0496% +/- 1.2577%*	*98.1123% +/- 0.7467%*

Figure 17: Summary of the MS2 net log and associated percent reduction values for the Beyond Guardian Air.

Results

The standard Beyond Guardian Air achieved an average of a 1.75 net log reduction in 20 minutes of operation, see **Figures 15 and 16** for a total graphical overview of both log and net log reduction while **Figure 17** presents a table summarizing the results.

Since ASHRAE 241 relies heavily on the calculated CADR value, it was calculated at various points during all the Beyond Guardian Air trials and during pre-testing. The pretests showed a relatively variable data set of the CADR calculations depending on the time points over which it was calculated. Initial CADR values are always considerably higher than the CADR values calculated later in the trial. Theoretically, this could be because of the technology associated with the Beyond Guardian Air unit. The efficacy of this technology is partially dependent on the production of ActivePure™ molecules (ions) that saturate the indoor environment. Saturation of the catalyst may occur during the test trials, since the contaminant concentration in the chamber is very high, thus limiting the production of ions. Ultimately, the true cause of this phenomenon is a bit unclear, but this trend caused the modification of the sampling times used for the test trials.

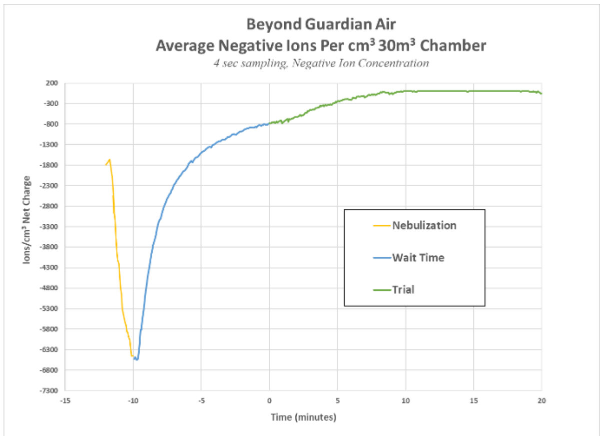

Figure 18: Summary of the MS2 net log and associated percent reduction values for the Beyond Guardian Air.

Ion Monitoring

Using the same ion monitor as in previous measurements, we tracked the production of negative ions throughout the ASHRAE 241 trial. **Figure 18** presents a clear view of how free negative ions accumulated and dissipated in the chamber during the trial. Different colors represent distinct phases of the test. Nebulization introduced the bioaerosol, and the waiting period allowed the chamber to mix before activating

the Beyond Guardian Air device. The trial reveals the behavior of negative ions, especially when the device is in operation showing an average of approximately – 485 from 0 to 6 minutes and - 300 from 0 to 20 minutes.

Deviations and Acceptance Criteria

No deviations from the ASHRAE 241 protocol were used. The chamber was tested every minute for 8 minutes and then at 12, 16 and 20 minutes. All final endpoints were ≤0.30 standard deviations from the mean. In accordance with ARE Lab's standard practices, and in compliance with GLP, all data was verified for accuracy. Neither ASHRAE 241 nor AHAM AC-5 have specific guidelines regarding standard deviation across triplicate trials.

Clean Air Delivery Rate Calculations (CADR)

The Clean Air Delivery Rate (CADR) is a measure of the efficiency of an air purifier for removing specific airborne pollutants from the indoor air. Expressed in cubic feet per minute (CFM), CADR provides information on the volume of clean air that an air purifier can deliver for various pollutants: tobacco smoke, pollen, dust, and biological pathogens. The CADR rating is determined through standardized testing procedures, and a higher CADR indicates a more effective air purifier in removing those contaminants from the air.

For CADR calculations, a linear fit trendline corresponding to a specific time interval was applied to both the trial and the control lines. The difference between these slopes was calculated to determine the equivalent air exchange rate. The time interval to calculate the linear regression slope of the test trials was determined using the t-0 and all time points through t-6 minutes, additionally the CADR was also calculated from t-0 and all time points for the entire trial out to t-20 minutes. The CADR was then calculated by multiplying this equivalent air exchange rate by the volume of the test chamber (30 m^3).

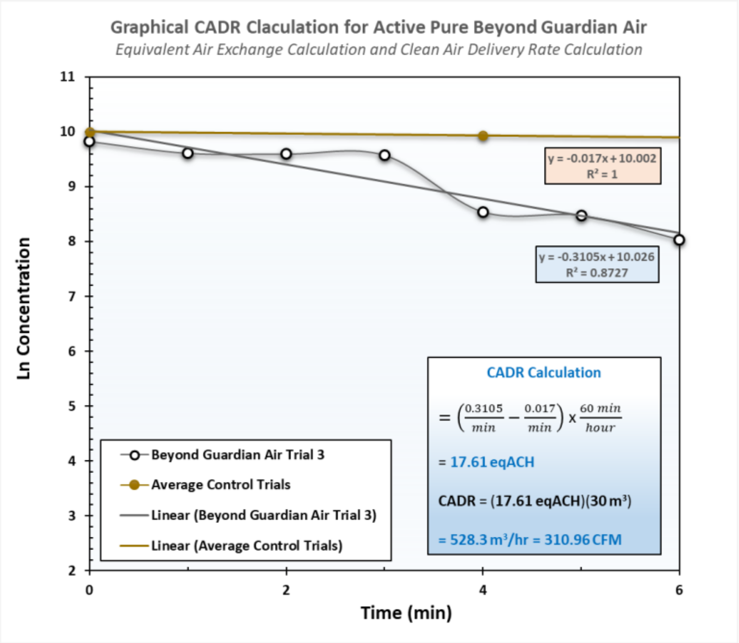

Figure 19: CADR Example Plot and Calculation for the Beyond Guardian Air.

Times used for CADR Calculations	T1	T2	T3	Average	STDEV
**0-6**	305.35	296.75	310.85	304.32	7.11
**0-20**	163.67	195.07	220.28	193.01	28.36

Figure 20: CADR summary for the Beyond Guardian Air showing the CADR calculated through a single time point against MS2.

CADR Observations

While testing the device against MS2, a pattern emerged where at the beginning of the test would result in a high CADR while the CADR calculations at the end of the trial would yield lower CADR numbers. Recognizing this, the test methodology was modified to sample faster earlier to capture and evaluate this pattern. Due to the device's mechanism of action using ionization as well as HEPA filtration, it was determined that the device provided a higher initial reduction and then leveled off more as the ions were consumed. This high initial reduction seems to be the result of the ions inactivating the organisms because HEPA filtration typically takes longer to have an effect because of the airflow passing through the device required to capture the bioaerosol. This justified the faster sampling method as well as the need to report two separate CADR values to capture the full effect of the ActivePure Beyond Guardian Air device.

CADR Result Summary

The CADR was analyzed across all trials with MS2 and *Staphylococcus epidermidis* for a triplicate set. The Beyond Guardian Air Trial 2 against MS2 had the highest CADR at 310.85 CFM from t-0 to t-6, but all the trials averaged to 304 CFM at that same time interval. However, the 0–20-minute CADR had a value of approximately 193 CFM as an average for all three trials. A graphical visualization of the CADR calculation with actual trial results can be found in **Figure 19** with a table breaking down the CADR at two separate time points in **Figure 20**.

S. epidermidis exhibited a similar trend to the MS2 trials having higher initial CADR values at the beginning of the trials, but then dropping as the trial went on to represent more of a steady state CADR. Specifically, trial 2 from t-0 to t-10 minutes, peaked at 406 CFM, although the average CADR across all trials using all time points was 340 CFM. By the end of the 60 minute duration, these trials averaged a steady state CADR value of 199 CFM and averaged all trial data points.

MS2 Results

The Beyond Guardian Air device performed well at removing viable MS2 from the air. It had a total reduction of 1.75 ± 0.19 net log, equaling a percent reduction of 98.1123%, from the air in 20 minutes of the device in operation and averaged a steady state CADR of 193 CFM over that same time frame. The first portion of the testing from t-0 to t-6 minutes had an average initial CADR averaging at 304 CFM. Both the initial and steady state CADR values tell a story about the technology utilized in the ActivePure Beyond Guardian Air unit and how it helps remove and inactivate air borne pathogens. Overall, the initial calculated CADR from the device surpassed the device's baseline flow rate of approximately 220 CFM for both organisms. A table breakdown of the MS2 CADR values at all time intervals is shown in **Figure 20**.

Since the Beyond Guardian Air is designed to be in continuous operation, the compounding effect of the ActivePure™ molecules should provide additional reduction in areas where standard pathogens are far below the tested concentration. This should result in a highly effective inactivation and removal of viable microorganisms from a given space.

Staphylococcus epidermidis Results

While the Beyond Guardian Air device was able to reduce the viability of MS2, an additional set of triplicate tests were performed with the challenge organism *Staphylococcus epidermidis*. These results show a higher recorded CADR and an overall better reduction of viable Staphylococcus in the chamber. The triplicate tests show an average of 5.12 ± 0.06 net log reduction of Staphylococcus from the chamber which is equivalent of a 99.99924% reduction after 60 minutes of device operation. Using the same methodology to calculate the CADR as used previously with MS2, the CADR was calculated from the t-0 to t-10 minutes of the triplicate trial. These results show an average steady state CADR of 340.55 CFM making it more effective at reducing *Staphylococcus epidermidis* than MS2. A small table showing the CADR for all 3 trials from t-0 to the t-6, t-10, and t-60 timepoints. These results can be found below in **Figure 21**.

Time Frame Used To Calculate	T1	T2	T3	Average	STDEV
**0-6**	359.6	320.2	251.2	310.33	54.87
**0-10**	340.83	406.62	274.20	340.55	66.21
**0-60**	180.01	208.09	209.34	199.14	16.58

Figure 21: CADR summary for the Beyond Guardian Air showing the CADR calculated through a single time point against *Staphylococcus epidermidis*.

Proprietary and Confidential - Property of ActivePure

References

AHAM. (2023). AHAM AC-5: Performance Evaluation of Room Air Cleaners. Association of Home Appliance Manufacturers.

ASHRAE 241. (July 2023). Control of Infection Aerosols. American Society of Heating, Refrigerating, and Air Conditioning Engineers.

Ding and Wing. (2001). Effects of Sampling Time on the Total Recovery Rate of AGI-30 Impingers for E. coli. Aerosol and Air Quality Research, Vol. 1, No. 1, pp. 31-36.

Feller, W. (1950). An Introduction to Probability Theory and Its Applications. Wiley.

T. Reponen, K. Willeke, V. Ulevicius et al. (1997). Techniques of Dispersion of Microorganisms in Air. Aerosol Science and Technology, 27, pp. 405-421.

U.S. Department of Health and Human Services Food and Drug Administration. (March 2009). Enforcement Policy for Sterilizers, Disinfectant Devices, and Air Purifiers During the Coronavirus Disease 2019 (COVID-19) Public Health Emergency Guidance for Industry and Food and Drug Administration Staff.

Dietrich, Watts L., et al. (2020). Laboratory Modeling of SARS-CoV-2 Exposure Reduction Through Physically Distanced Seating in Aircraft Cabins Using Bacteriophage Aerosol — November 2020. Morbidity and Mortality Weekly Report, 69(46), pp. 1744-1750.

Analytical Testing Facility

Aerosol Research and Engineering Labs, Inc.
12880 Metcalf Ave
Overland Park, KS 66213

Project

10860.30.1.1

Study Director

Richard Ludwick
Aerosol Research and Engineering Laboratories

GLP Statement

We, the undersigned, hereby certify that the work described herein was conducted by Aerosol Research and Engineering Laboratories in compliance with ASHRAE 241, AHAM AC-5, and Good Laboratory Practices (GLP) as defined in 21 CFR, Part 58.

Conflict of Interest Statement

Aerosol Research and Engineering Laboratories, Inc. have no affiliations with, or involvement in any capacity, with ActivePure's financial interests such as; membership, employment, stock ownership, or other equity interest.

Study Director:

Richard K. Ludwick [signature]

Richard Ludwick
Study Director
ARE Labs, Inc.

01/08/2024
Date

Principal Investigator:

W. Andrew Dexter [signature]

W. Andrew Dexter M.S.
Staff Research Scientist
ARE Labs, Inc.

01/08/2024
Date

APPENDIX A: Bio Aerosol Raw Data

Trial Information

TEST DATE: Wednesday, November 15, 2023
TRIAL PERFORMED BY: ZT
TRIAL NUMBER: T1
TEST ORGANSIM: MS2
TRIAL NAME ID (GRAPHS/TABLES): MS2 T1

Device Information

MANUFACTURER: ActivePure
UNIT MODEL: Beyond Guardian Air
FAN SPEED (CFM):
UNIT SERIAL #:
FITER ID #:
FILTER LOT #:

General Testing Conditions (Can Be User Defined)

TEST CHAMBER VOLUME (m^3): 30
NEBULIZER CONDITIONS: Collison 24-Jet; approx. 2 min neb
SAMPLING METHOD: Impinger
CHAMBER MIXING FAN: yes
TEMP (F): 74
RH (%): 70
OTHER INSTRUMENTS:
TRIAL COMMENTS/NOTES

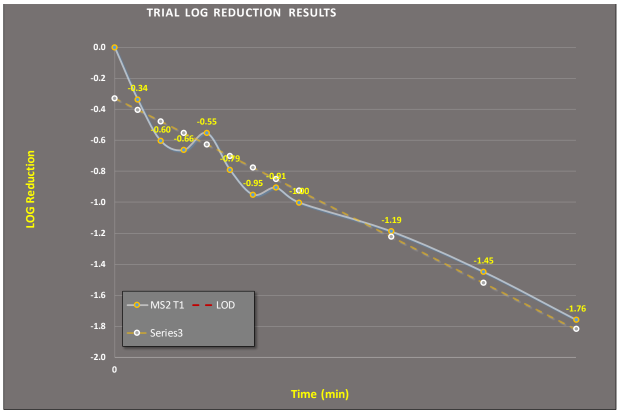

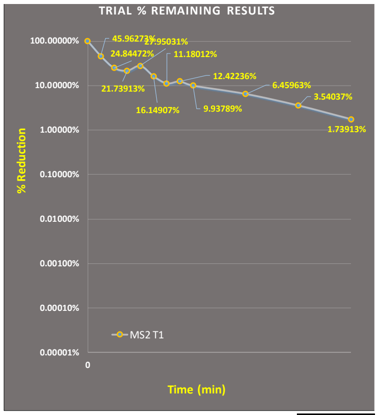

BIOAEROSOL Sample ID and Summary Data	S1	S2	S3	S4	S5	S6	S7	S7	S8	S9	S10	S8	LOD
SAMPLE TIME (min)	0	1	2	3	4	5	6	7	8	12	16	20	LOD
IMPINGER USED (y / n)	y	y	y	y	y	y	y	y	y	y	y	y	y
VIABLE CASCADE USED (y / n)	n	n	n	n	n	n	n	n	n	n	n	n	n
CHAMBER IMPINGER BIOAEROSOL CONCENTRATION (cfu pfu/L Air)	1.717E+04	7.893E+03	4.267E+03	3.733E+03	4.800E+03	2.773E+03	1920.000	2133.333	1706.667	1109.333	608.000	298.667	1.422E-01
CHAMBER VIABLE BIOAEROSOL CONCENTRATION (cfu or pfu/L Air)													
IMPINGER DILUTION CONSISTENCY CHECKS (% agreement)	40.59%	31.82%	0.00%	25.00%	87.50%		20.00%			7.41%	10.00%	25.00%	100.00%
VIABLE CONSISTENCY CHECKS (% agreement)													
IMP & VIABLE CROSS CHECK (% agreement)													
**CHAMBER BIOAEROSOL CONCENTRATION (cfu or pfu/L Air)**	**1.717E+04**	**7.893E+03**	**4.267E+03**	**3.733E+03**	**4.800E+03**	**2.773E+03**	**1.920E+03**	**2.133E+03**	**1.707E+03**	**1.109E+03**	**6.080E+02**	**2.987E+02**	**0.1422**
**RELATIVE PERCENT REMAINING FROM T=0 (%)**	**100.0000%**	**45.9627%**	**24.8447%**	**21.7391%**	**27.9503%**	**16.1491%**	**11.1801%**	**12.4224%**	**9.9379%**	**6.4596%**	**3.5404%**	**1.7391%**	**0.0008%**
**RELATIVE PERCENT REMOVAL FROM T=0 (%)**	**0.0000%**	**54.0373%**	**75.1553%**	**78.2609%**	**72.0497%**	**83.8509%**	**88.8199%**	**87.5776%**	**90.0621%**	**93.5404%**	**96.4596%**	**98.2609%**	**99.9992%**
**LOG REDUCTION FROM T=0 (log_{10})**	**0.00**	**-0.34**	**-0.60**	**-0.66**	**-0.55**	**-0.79**	**-0.95**	**-0.91**	**-1.00**	**-1.19**	**-1.45**	**-1.76**	**-5.08**

Impinger Sampling Conditions

	SAMPLE TIME (min)	0	1	2	3	4	5	6	7	8	12	16	20	LOD
	IMPINGER FILL VOL (ml)	20.0	20.0	20.0	20.0	20.0	20.0	20.0	20.0	20.0	20.0	20.0	20.0	20.0
	IMPINGER SAMPLING TIME (min)	1.0	1.0	1.0	1.0	1.0	1.0	1.0	1.0	1.0	1.0	1.0	1.0	5.0
	IMPINGER FLOW RATE (lpm)	12.5	12.5	12.5	12.5	12.5	12.5	12.5	12.5	12.5	12.5	12.5	12.5	12.5
Dilution Range #1	**DILUTION RATIO (10^x)**	**-3**	**-3**	**-3**	**-3**	**-3**	**-3**	**-3**	**-3**	**-3**	**-1**	**-1**	**0**	**0**
	**DROPLET SIZE (µl)**	**750**	**750**	**750**	**750**	**750**	**750**	**750**	**750**	**750**	**750**	**750**	**750**	**750**
	ENUMERATED PLATE COUNTS (# / drop)	6	3	2	2	4		1			54	27	160	1<br>0<br>0
	PLATE AVERAGE COUNT (# / drop)	6.00	3.00	2.00	2.00	4.00		1.00			54.00	27.00	160.00	0.33
	IMPINGER CONCENTRATION (cfu or pfu/ml)	8,000	4,000	2,667	2,667	5,333		1,333			720	360	213	0
	CHAMBER BIOAEROSOL CONCETRATION (cfu or pfu/L Air)	**1.28E+04**	**6.40E+03**	**4.27E+03**	**4.27E+03**	**8.53E+03**		**2.13E+03**			**1.15E+03**	**5.76E+02**	**3.41E+02**	**1.42E-01**
Dilution Range #1	**DILUTION RATIO (10^x)**	**-2**	**-2**	**-2**	**-2**	**-2**	**-2**	**-2**	**-2**	**-2**	**-2**	**-2**	**-1**	
	**DROPLET SIZE (µl)**	**750**	**750**	**750**	**750**	**750**	**750**	**750**	**750**	**750**	**750**	**750**	**750**	
	ENUMERATED PLATE COUNTS (# / drop)	101	44	20	15	5	13	8	10	8	5	3	12	
	PLATE AVERAGE COUNT (# / drop)	101.00	44.00	20.00	15.00	5.00	13.00	8.00	10.00	8.00	5.00	3.00	12.00	
	IMPINGER CONCENTRATION (cfu or pfu/ml)	13,467	5,867	2,667	2,000	667	1,733	1,067	1,333	1,067	667	400	160	
	CHAMBER BIOAEROSOL CONCETRATION (cfu or pfu/L Air)	**2.15E+04**	**9.39E+03**	**4.27E+03**	**3.20E+03**	**1.07E+03**	**2.77E+03**	**1.71E+03**	**2.13E+03**	**1.71E+03**	**1.07E+03**	**6.40E+02**	**2.56E+02**	

Figure 1A: *ActivePure Beyond Guardian Air Raw Data T1.*

Trial Information

TEST DATE: Wednesday, November 15, 2023
TRIAL PERFORMED BY: ZT
TRIAL NUMBER: T2
TEST ORGANSIM: MS2
TRIAL NAME ID (GRAPHS/TABLES): MS2 T2

Device Information

MANUFACTURER: ActivePure
UNIT MODEL: Beyond Guardian Air
FAN SPEED (CFM):
UNIT SERIAL #:
FITER ID #:
FILTER LOT #:

General Testing Conditions (Can Be User Defined)

TEST CHAMBER VOLUME (m^3): 30
NEBULIZER CONDITIONS: Collison 24-Jet; approx. 2 min neb
SAMPLING METHOD: Impinger
CHAMBER MIXING FAN: yes
TEMP (F): 74
RH (%): 70
OTHER INSTRUMENTS:
TRIAL COMMENTS/NOTES

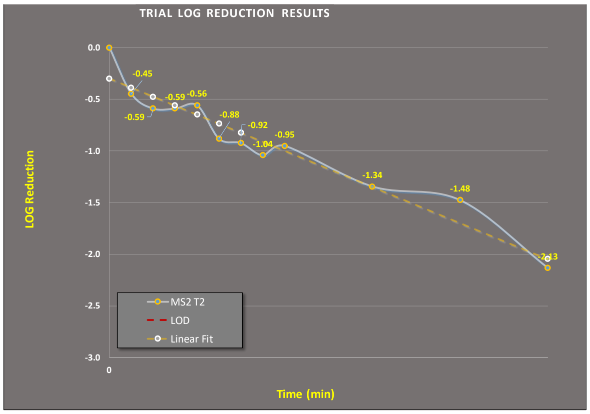

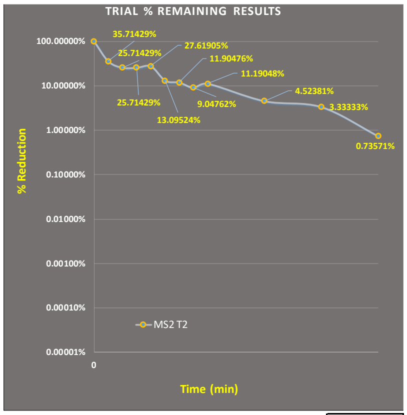

BIOAEROSOL Sample ID and Summary Data	S1	S2	S3	S4	S5	S6	S7	S7	S8	S9	S10	S8	LOD
SAMPLE TIME (min)	0	1	2	3	4	5	6	7	8	12	16	20	LOD
IMPINGER USED (y / n)	y	y	y	y	y	y	y	y	y	y	y	y	y
VIABLE CASCADE USED (y / n)	n	n	n	n	n	n	n	n	n	n	n	n	n
CHAMBER IMPINGER BIOAEROSOL CONCENTRATION (cfu pfu/L Air)	4.480E+04	1.600E+04	1.152E+04	1.152E+04	1.237E+04	5.867E+03	5333.333	4053.333	5013.333	2026.667	1493.333	329.600	1.422E-01
CHAMBER VIABLE BIOAEROSOL CONCENTRATION (cfu or pfu/L Air)													
IMPINGER DILUTION CONSISTENCY CHECKS (% agreement)	9.09%	50.00%	13.79%	20.00%		42.86%	33.33%	10.00%	88.10%	10.00%	60.00%	6.88%	100.00%
VIABLE CONSISTENCY CHECKS (% agreement)													
IMP & VIABLE CROSS CHECK (% agreement)													
**CHAMBER BIOAEROSOL CONCENTRATION (cfu or pfu/L Air)**	**4.480E+04**	**1.600E+04**	**1.152E+04**	**1.152E+04**	**1.237E+04**	**5.867E+03**	**5.333E+03**	**4.053E+03**	**5.013E+03**	**2.027E+03**	**1.493E+03**	**3.296E+02**	**0.1422**
**RELATIVE PERCENT REMAINING FROM T=0 (%)**	**100.0000%**	**35.7143%**	**25.7143%**	**25.7143%**	**27.6190%**	**13.0952%**	**11.9048%**	**9.0476%**	**11.1905%**	**4.5238%**	**3.3333%**	**0.7357%**	**0.0003%**
**RELATIVE PERCENT REMOVAL FROM T=0 (%)**	**0.0000%**	**64.2857%**	**74.2857%**	**74.2857%**	**72.3810%**	**86.9048%**	**88.0952%**	**90.9524%**	**88.8095%**	**95.4762%**	**96.6667%**	**99.2643%**	**99.9997%**
**LOG REDUCTION FROM T=0 (log_{10})**	**0.00**	**-0.45**	**-0.59**	**-0.59**	**-0.56**	**-0.88**	**-0.92**	**-1.04**	**-0.95**	**-1.34**	**-1.48**	**-2.13**	**-5.50**

Impinger Sampling Conditions

SAMPLE TIME (min)	0	1	2	3	4	5	6	7	8	12	16	20	LOD
IMPINGER FILL VOL (ml)	20.0	20.0	20.0	20.0	20.0	20.0	20.0	20.0	20.0	20.0	20.0	20.0	20.0
IMPINGER SAMPLING TIME (min)	1.0	1.0	1.0	1.0	1.0	1.0	1.0	1.0	1.0	1.0	1.0	1.0	5.0
IMPINGER FLOW RATE (lpm)	12.5	12.5	12.5	12.5	12.5	12.5	12.5	12.5	12.5	12.5	12.5	12.5	12.5

Dilution Range #1													
**DILUTION RATIO (10^x)**	**-3**	**-3**	**-3**	**-3**	**-3**	**-3**	**-3**	**-3**	**-3**	**-3**	**-3**	**-1**	**0**
**DROPLET SIZE (µl)**	**750**	**750**	**750**	**750**	**750**	**750**	**750**	**750**	**750**	**750**	**750**	**750**	**750**
ENUMERATED PLATE COUNTS (# / drop)	22	5	5	6		2	3	2	0<br>1	1	1	16	1<br>0<br>0
PLATE AVERAGE COUNT (# / drop)	22.00	5.00	5.00	6.00		2.00	3.00	2.00	0.50	1.00	1.00	16.00	0.33
IMPINGER CONCENTRATION (cfu or pfu/ml)	29,333	6,667	6,667	8,000		2,667	4,000	2,667	667	1,333	1,333	213	0
CHAMBER BIOAEROSOL CONCETRATION (cfu or pfu/L Air)	**4.69E+04**	**1.07E+04**	**1.07E+04**	**1.28E+04**		**4.27E+03**	**6.40E+03**	**4.27E+03**	**1.07E+03**	**2.13E+03**	**2.13E+03**	**3.41E+02**	**1.42E-01**

Dilution Range #1													
**DILUTION RATIO (10^x)**	**-4**	**-4**	**-2**	**-2**	**-2**	**-2**	**-2**	**-2**	**-2**	**-2**	**-2**	**0**	
**DROPLET SIZE (µl)**	**750**	**750**	**750**	**750**	**750**	**750**	**750**	**750**	**750**	**750**	**750**	**750**	
ENUMERATED PLATE COUNTS (# / drop)	2	1	58	48	58	35	20	18	42	9	4	149	
PLATE AVERAGE COUNT (# / drop)	2.00	1.00	58.00	48.00	58.00	35.00	20.00	18.00	42.00	9.00	4.00	149.00	
IMPINGER CONCENTRATION (cfu or pfu/ml)	26,667	13,333	7,733	6,400	7,733	4,667	2,667	2,400	5,600	1,200	533	199	
CHAMBER BIOAEROSOL CONCETRATION (cfu or pfu/L Air)	**4.27E+04**	**2.13E+04**	**1.24E+04**	**1.02E+04**	**1.24E+04**	**7.47E+03**	**4.27E+03**	**3.84E+03**	**8.96E+03**	**1.92E+03**	**8.53E+02**	**3.18E+02**	

Figure 2A: *ActivePure Beyond Guardian Air Raw Data T2.*

Proprietary and Confidential - Property of ActivePure

Trial Information

TEST DATE: Wednesday, November 15, 2023
TRIAL PERFORMED BY: ZT
TRIAL NUMBER: T3
TEST ORGANSIM: MS2
TRIAL NAME ID (GRAPHS/TABLES): MS2 T3

Device Information

MANUFACTURER: ActivePure
UNIT MODEL: Beyond Guardian Air
FAN SPEED (CFM):
UNIT SERIAL #:
FITER ID #:
FILTER LOT #:

General Testing Conditions (Can Be User Defined)

TEST CHAMBER VOLUME (m^3): 30
NEBULIZER CONDITIONS: Collison 24-Jet; approx. 2 min neb
SAMPLING METHOD: Impinger
CHAMBER MIXING FAN: yes
TEMP (F): 74
RH (%): 70
OTHER INSTRUMENTS:
TRIAL COMMENTS/NOTES

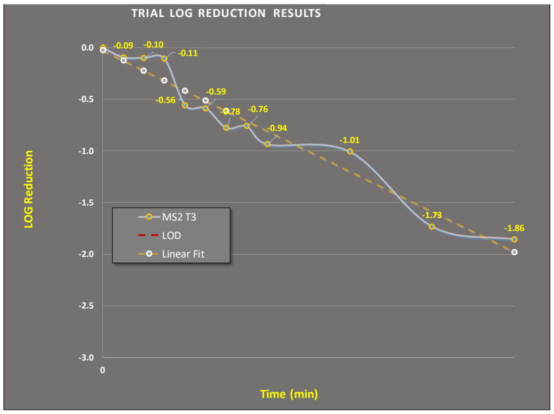

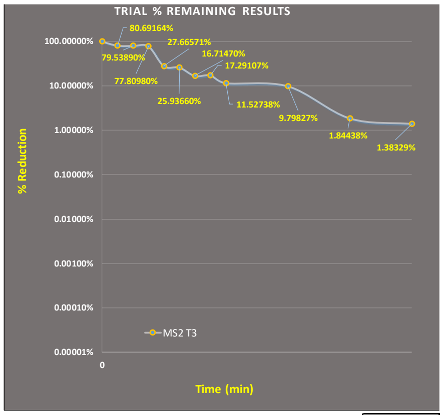

BIOAEROSOL Sample ID and Summary Data

	S1	S2	S3	S4	S5	S6	S7	S7	S8	S9	S10	S8	LOD
SAMPLE TIME (min)	0	1	2	3	4	5	6	7	8	12	16	20	LOD
IMPINGER USED (y / n)	y	y	y	y	y	y	y	y	y	y	y	y	y
VIABLE CASCADE USED (y / n)	n	n	n	n	n	n	n	n	n	n	n	n	n
CHAMBER IMPINGER BIOAEROSOL CONCENTRATION (cfu pfu/L Air)	1.851E+04	1.493E+04	1.472E+04	1.440E+04	5.120E+03	4.800E+03	3093.333	3200.000	2133.333	1813.333	341.333	256.000	1.422E-01
CHAMBER VIABLE BIOAEROSOL CONCENTRATION (cfu or pfu/L Air)													
IMPINGER DILUTION CONSISTENCY CHECKS (% agreement)	7.22%	60.00%		20.00%	28.57%	20.00%	47.37%	50.00%		58.33%			100.00%
VIABLE CONSISTENCY CHECKS (% agreement)													
IMP & VIABLE CROSS CHECK (% agreement)													
**CHAMBER BIOAEROSOL CONCENTRATION (cfu or pfu/L Air)**	1.851E+04	1.493E+04	1.472E+04	1.440E+04	5.120E+03	4.800E+03	3.093E+03	3.200E+03	2.133E+03	1.813E+03	3.413E+02	2.560E+02	0.1422
**RELATIVE PERCENT REMAINING FROM T=0 (%)**	100.0000%	80.6916%	79.5389%	77.8098%	27.6657%	25.9366%	16.7147%	17.2911%	11.5274%	9.7983%	1.8444%	1.3833%	0.0008%
**RELATIVE PERCENT REMOVAL FROM T=0 (%)**	0.0000%	19.3084%	20.4611%	22.1902%	72.3343%	74.0634%	83.2853%	82.7089%	88.4726%	90.2017%	98.1556%	98.6167%	99.9992%
**LOG REDUCTION FROM T=0 (log_{10})**	0.00	-0.09	-0.10	-0.11	-0.56	-0.59	-0.78	-0.76	-0.94	-1.01	-1.73	-1.86	-5.11

Impinger Sampling Conditions

SAMPLE TIME (min)	0	1	2	3	4	5	6	7	8	12	16	20	LOD
IMPINGER FILL VOL (ml)	20.0	20.0	20.0	20.0	20.0	20.0	20.0	20.0	20.0	20.0	20.0	20.0	20.0
IMPINGER SAMPLING TIME (min)	1.0	1.0	1.0	1.0	1.0	1.0	1.0	1.0	1.0	1.0	1.0	1.0	5.0
IMPINGER FLOW RATE (lpm)	12.5	12.5	12.5	12.5	12.5	12.5	12.5	12.5	12.5	12.5	12.5	12.5	12.5

Dilution Range #1	0	1	2	3	4	5	6	7	8	12	16	20	LOD
**DILUTION RATIO (10^x)**	-3	-3	-3	-3	-3	-3	-3	-3	-3	-3	-1	-1	0
**DROPLET SIZE (µl)**	750	750	750	750	750	750	750	750	750	750	750	750	750
ENUMERATED PLATE COUNTS (# / drop)	9	4		6	2	2	1	1	1	1<br>0	16	12	1<br>0<br>0
PLATE AVERAGE COUNT (# / drop)	9.00	4.00		6.00	2.00	2.00	1.00	1.00	1.00	0.50	16.00	12.00	0.33
IMPINGER CONCENTRATION (cfu or pfu/ml)	12,000	5,333		8,000	2,667	2,667	1,333	1,333	1,333	667	213	160	0
CHAMBER BIOAEROSOL CONCETRATION (cfu or pfu/L Air)	1.92E+04	8.53E+03		1.28E+04	4.27E+03	4.27E+03	2.13E+03	2.13E+03	2.13E+03	1.07E+03	3.41E+02	2.56E+02	1.42E-01

Dilution Range #1	0	1	2	3	4	5	6	7	8	12	16	20	LOD
**DILUTION RATIO (10^x)**	-2	-4	-2	-2	-2	-2	-2	-2	-2	-2	-2	-2	
**DROPLET SIZE (µl)**	750	750	750	750	750	750	750	750	750	750	750	750	
ENUMERATED PLATE COUNTS (# / drop)	84<br>83	1	69	75	28	25	19	20		12			
PLATE AVERAGE COUNT (# / drop)	83.50	1.00	69.00	75.00	28.00	25.00	19.00	20.00		12.00			
IMPINGER CONCENTRATION (cfu or pfu/ml)	11,133	13,333	9,200	10,000	3,733	3,333	2,533	2,667		1,600			
CHAMBER BIOAEROSOL CONCETRATION (cfu or pfu/L Air)	1.78E+04	2.13E+04	1.47E+04	1.60E+04	5.97E+03	5.33E+03	4.05E+03	4.27E+03		2.56E+03			

Figure 3A: *ActivePure Beyond Guardian Air Raw Data T3.*

Proprietary and Confidential - Property of ActivePure

Appendix B: Calculations

To evaluate the viable aerosol delivery efficiency and define operation parameters of the system, calculations based on (theoretical) 100% efficacy of aerosol dissemination were derived using the following steps:

- Plating and enumeration of the biological to derive the concentration of the stock suspension (C_s) in pfu/mL or cfu/mL, or cfu/g for dry powder.
- Collison 24 jet nebulizer use rate (R_{neb}) (volume of liquid generated by the nebulizer/time) at 28 psi air supply pressure = 1.0 mL/min.
- Collison 24 jet Generation time (t) = 20 or 30 minutes, test dependent.
- Chamber volume (V_c) = 15,993 Liters

Assuming 100% efficiency, the quantity of aerosolized viable particles (V_P) per liter of air in the chamber for a given nebulizer stock concentration (C_s) is calculated as:

$$\text{Nebulizer: } V_P = \frac{C_s \cdot R_{neb}}{V_c} t$$

Plating and enumeration of the biological to derive the concentration of the dry powder (C_p) in cfu/g.

- Eductor use rate (M_p) (Mass of powder generated by the eductor in grams)
- Chamber volume (V_c) = 15,993 Liters

Assuming 100% efficiency, the quantity of aerosolized viable particles (V_P) per liter of air in the chamber for a given dry powder stock concentration (C_p) is calculated as:

$$\text{Eductor: } V_P = \frac{C_p \cdot M_p}{V_c}$$

AGI – 30 impinger or 47mm filter collection calculation:

- Viable aerosol concentration collection (C_a) = cfu or pfu/L of chamber air.
- Viable Impinger concentration collection (C_{Imp}) = cfu or pfu/mL from enumeration of impinger sample or filter sample.
- Impinger sample collection volume (I_{vol}) = 20 mL collection fluid/impinger, or extraction fluid for filter.
- AGI–30 impinger or filter sample flow rate (Q_{imp}) = 12.5 L/min.
- AGI–30 impinger or filter sample time (t) = 5 or 10 minutes, test dependent.

For viable impinger or filter aerosol concentration collection (C_a) = cfu or pfu/L of chamber air:

$$C_a = \frac{C_{Imp} \cdot I_{vol}}{Q_{imp}} t$$

The aerosol system viable delivery efficiency (expressed as %) is:

$$Efficiency = \frac{C_a}{V_p} \cdot 100$$

The table below is based on the principle that, as the number of viable particles being impinged on a given plate increases, the probability of the next particle going into an “empty hole” decreases.
This can be corrected statistically by using the conversion formula of Feller [4]:

Pr = N [1/N + 1/N-1 + 1/N-2 + ... 1/N-r+1]

N is the number of holes (400) in the sampling head.
For easy use of this formula please refer to the table in chapter 17.2
For each colony count **r** a statistically corrected total count **Pr** can be easily seen in the table.
