# Supplementary material for: Evaluate the safety of a novel photohydrolysis technology used to clean and disinfect indoor air: A murine study
Source: PLoS One. 2024 Oct 9;19(10):e0307031. doi: 10.1371/journal.pone.0307031 (PMC11463749; doi:10.1371/journal.pone.0307031)
Supplement: S11 File — (PDF) [file pone.0307031.s011.pdf]

intertek
Total Quality. Assured.

Aerus Medical LLC
OZONE TEST REPORT

SCOPE OF WORK
Ozone Emissions Testing of Household Electrostatic Air Cleaners for Model: F170A Aerus Medical Guardian

REPORT NUMBER
103842941CRT-001

ISSUE DATE
28-Feb-2019

PAGES
13

QUOTE NUMBER
Qu-00956900-5

DOCUMENT CONTROL NUMBER
GFT-OP-10o (16-Oct-2017)
© 2019 INTERTEK

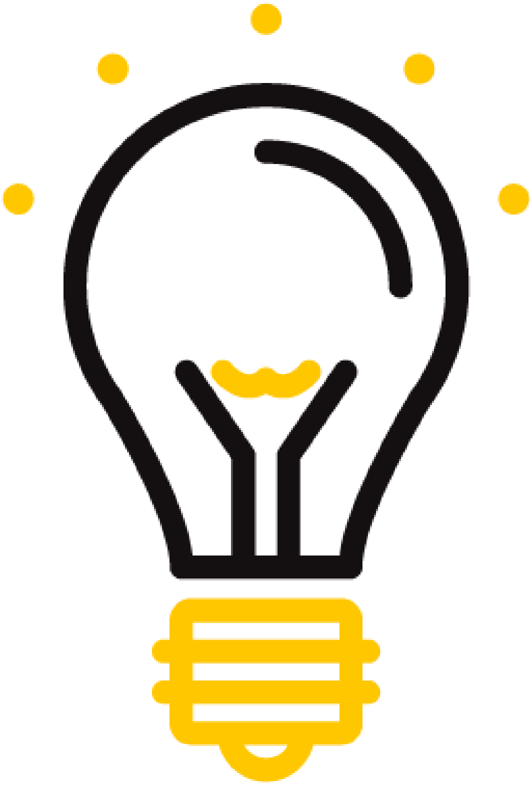

intertek
Total Quality. Assured.

3933 US Route 11
Cortland, NY 13045

Telephone: +01 607 753 6711
Facsimile: +01 607 756 9891
www.intertek.com

TEST REPORT FOR AERUS MEDICAL LLC

Report No.: 103842941CRT-001
Date: February 28, 2019

Lester Wise
Aerus Medical LLC
300 East Valley Drive
Bristol, VA 24201
USA

Phone: 276-645-2762
Email: lwise@aerusonline.com

SECTION 1

SUMMARY

The representative sample(s) have been tested, investigated, and found to comply with the requirements of the following Standard(s):

> *Electrostatic Air Cleaners, UL 867*, ***Section 40***, *Fifth Edition, August 4, 2011 revision: September 16, 2016.*

The equipment identified in this report has been found to meet the criteria for emittance of ozone not exceeding a concentration of 0.050 ppm. Furthermore, a second sample was not required to be tested, according to UL 867, as the first sample's maximum emissions were less than 0.030 ppm, which satisfies the exception in the Section 40.1.1.

This report completes our evaluation covered by Intertek Project Number G103842941which has been authorized by Intertek quote number: Qu-00956900-5. If there are any questions regarding the results contained in this report, or any of the other services offered by Intertek, please do not hesitate to contact the undersigned.

OZONE EMISSIONS SUMMARY

FAN SPEED	FILTER(S)	03/VOLTAGE SETTING	$C(t)_{max}$ [ppm]
High	YES	-	0.000
Low	YES	-	0.001
Low	NO	-	0.001

Completed by:	Joseph Hartley	Reviewed by:	Jacob Langenbacher
Title:	Technician III	Title:	Engineer
Signature:		Signature:	
Date	2/27/2019	Date:	2/28/2018

This report is for the exclusive use of Intertek's Client and is provided pursuant to the agreement between Intertek and its Client. Intertek's responsibility and liability are limited to the terms and conditions of the agreement. Intertek assumes no liability to any party, other than to the Client in accordance with the agreement, for any loss, expense or damage occasioned by the use of this report. Only the Client is authorized to permit copying or distribution of this report and then only in its entirety. Any use of the Intertek name or one of its marks for the sale or advertisement of the tested material, product or service must first be approved in writing by Intertek. The observations and test results in this report are relevant only to the sample tested. This report by itself does not imply that the material, product, or service is or has ever been under an Intertek certification program.

SECTION 2

INDEX

Section Names	PAGE
1: Summary/Signatures	2
2: Index/ Chamber Equipment Information	3
3: Unit under test information	4
4: Peak test	5
5: Max Test(s) Information	7
6: Appendices	10
7: Revisions	13

CHAMBER EQUIPMENT INFORMATION

TEST EQUIPMENT LIST

Instrument	Model	Intertek Ctrl #	Cal Due Date
Teledyne – Advanced Pollution Instrumentation Ozone Calibrator	703E	O204	09-28-2019
Teledyne – Advanced Pollution Instrumentation Ozone Monitor	400E	O202	*
Vaisala – Temperature & Humidity Transducer	HMD-70Y	T1307	06-05-2019
Fluid Components International- Flow meter	ST75V	D713	08-28-2019

* The 400E Ozone Monitor is calibrated using the 703E calibrator.

SECTION 3

UNIT UNDER TEST INFORMATION

MODEL INFORMATION			
Manufacturer:	Aerus Medical LLC	Pre-Filter:	No
Model Number:	F170A	HEPA Filter:	Yes
Production/Prototype/ Design	Production	ESP Filter:	No
Fan Speeds:	4	Carbon Filter:	Yes
O3/Voltage Settings:	-	UV Light:	Yes
O3 Monitor:	=	Ionizer:	Yes
Model Notes:	Model is representative of the Medical Guardian Model F170A		

RUN-IN TEST			
FIRST SAMPLE			
Run-in Start:	4:30 PM 2/20/2019	Run-in End:	4:30 PM 2/22/2019
Run-in Temperature:	77 ± 4 degF	Tracking Number:	CRT1902201607-001
Serial Number:	00003	Manufacture Date:	2/15/2019
Sample Notes:			
SECOND SAMPLE			
Run-in Start:	NA	Run-in End:	NA
Run-in Temperature:	NA	Tracking Number	CRT1902201607-002
Serial Number	NA	Manufacture Date:	2/15/2019
Sample Notes:	Per the exception listed under clause 40.1.1 of UL 867, the second sample was not required to be tested.		

SECTION 4

PEAK OZONE TEST

GRILL AND AIR PERIPHERY DIMENSIONS			
		Date of Test:	2/22/2019
Grill Height:	13.500	Air Periphery Height:	13.500
Grill Width:	9.500	Air Periphery Width:	9.500
Estimated Grill Area:	128.250	Est. Air Periphery Area:	128.250
Notes:	Measurements are in Inches		

PEAK LOCATION

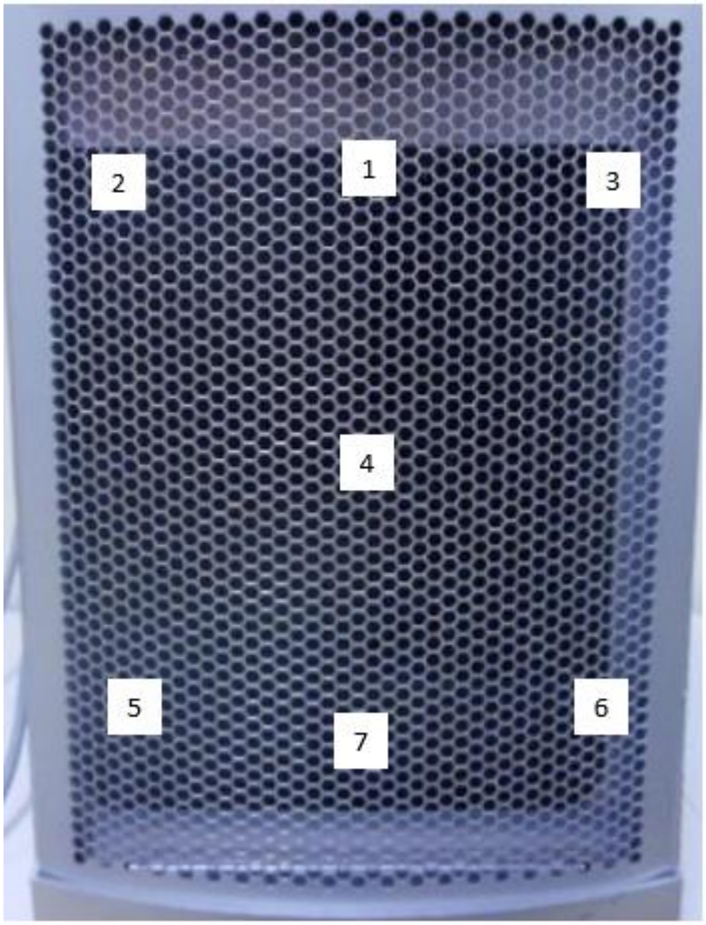

Loc.	X	Y
-	[inches]	[inches]
1	0	4.25
2	-4	4
3	4	4
4	0	0
5	-4	-4
6	4	-4
7	0	-4.8750

* Location measurements are coordinates in reference to the center point.

PEAK OZONE CONCENTRATIONS (ppm)				
Location	*With Filter(s)*		*Without Filter(s)*	
	Highest	*Lowest*	*Lowest*	
1	0.0001	0.0015	0.0001	
2	0.0004	0.0016	0.0006	
3	0.0005	0.0013	0.0001	
4	0.0006	0.0006	0.0002	
5	0.0004	0.0013	0.0007	
6	**0.0008**	**0.0019**	**0.0008**	
7	0.0006	0.0006	0.0001	

Note: Peak Ozone Test concentrations are shown with background subtracted.

SECTION 5

MAX OZONE TEST

START DATE OF TEST: 2/22/2019
SAMPLE: First Sample
FAN SPEED: 4 (Highest)
FILTER(S): Carbon and HEPA Filter installed, UV light and Ionizer ON

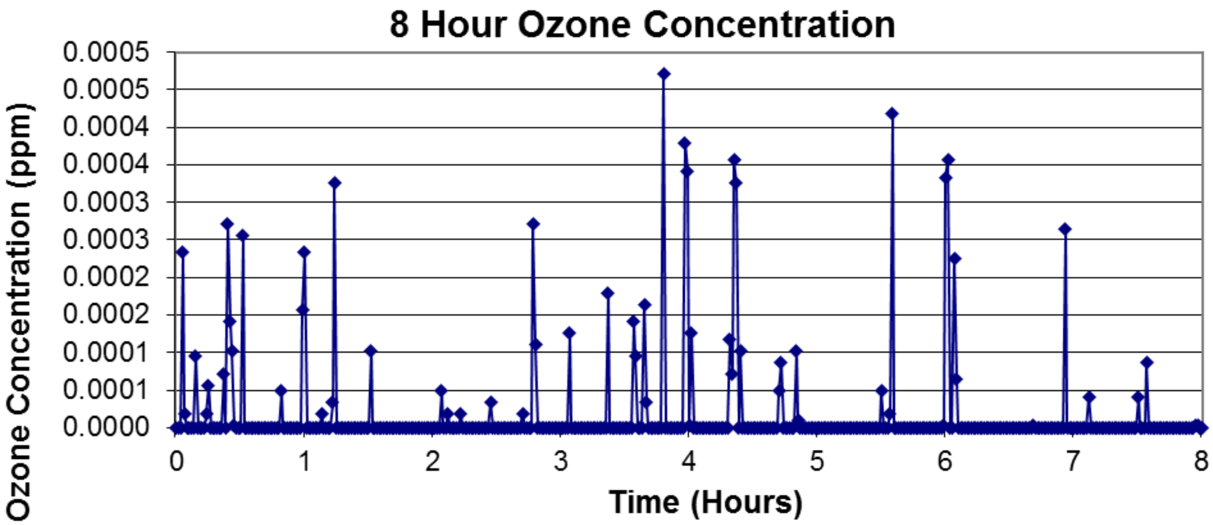

MAXIMUM OZONE TEST RESULTS							
	**UL Ref.**	**Pass/Fail**	**Mean**	**Min**	**Max**	**Delta**	**Units**
Background C(t) O3:	40.4.3	PASS	0.001	0.000	0.002	0.002	[ppm]
Test 1min C(t) O3:	40.1.2	PASS	0.000	0.000	**0.000**	0.000	[ppm]
Test 5min C(t) O3:	40.1.2	PASS	0.000	0.000	0.001	0.001	[ppm]
Chamber Temperature:	40.4.2	PASS	77	77	78	1	[degF]
Chamber Humidity:	40.4.2	PASS	50	49	52	3	[%RH]
Chamber Static Pressure:	-	PASS	0.02	0.01	0.03	0.03	["H2O]
Chamber Supply Air Flow:	-	-	20	19	20	0	[SCFM]
Required to Test 2nd Sample:	40.1.1	NO					
Test Duration:	*40.4.6	8 hours					

NOTES: Peak Test Location 6

MAX OZONE TEST

START DATE OF TEST: 2/23/2019
SAMPLE: First Sample
FAN SPEED: 1 (Lowest)
FILTER(S): Carbon and HEPA Filter installed, UV light and Ionizer ON

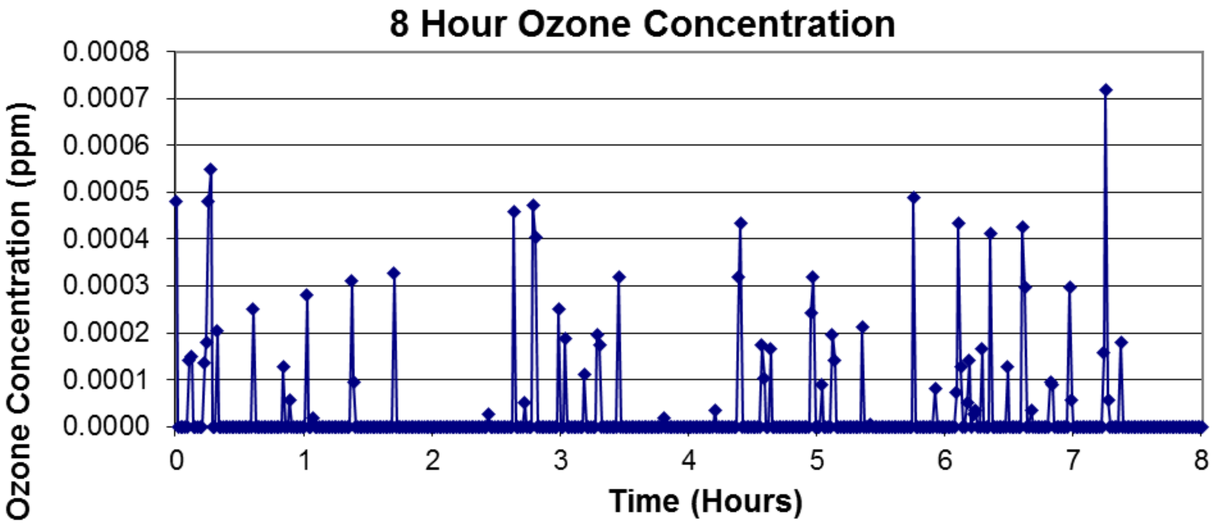

MAXIMUM OZONE TEST RESULTS							
	UL Ref.	Pass/Fail	Mean	Min	Max	Delta	Units
Background C(t) O3:	40.4.3	PASS	0.001	0.001	0.002	0.001	[ppm]
Test 1min C(t) O3:	40.1.2	PASS	0.000	0.000	**0.001**	0.001	[ppm]
Test 5min C(t) O3:	40.1.2	PASS	0.000	0.000	0.001	0.001	[ppm]
Chamber Temperature:	40.4.2	PASS	77	77	77	1	[degF]
Chamber Humidity:	40.4.2	PASS	50	49	52	3	[%RH]
Chamber Static Pressure:	-	PASS	0.02	0.00	0.03	0.03	["H2O]
Chamber Supply Air Flow:	-	-	20	19	20	0	[SCFM]
Required to Test 2nd Sample:	40.1.1	NO					
Test Duration:	*40.4.6	8 hours					

NOTES: Peak Test Location 6

MAX OZONE TEST

START DATE OF TEST: 2/24/2019
SAMPLE: First Sample
FAN SPEED: 1 (Lowest)
FILTER(S): Carbon and HEPA Filter removed, UV light and Ionizer ON

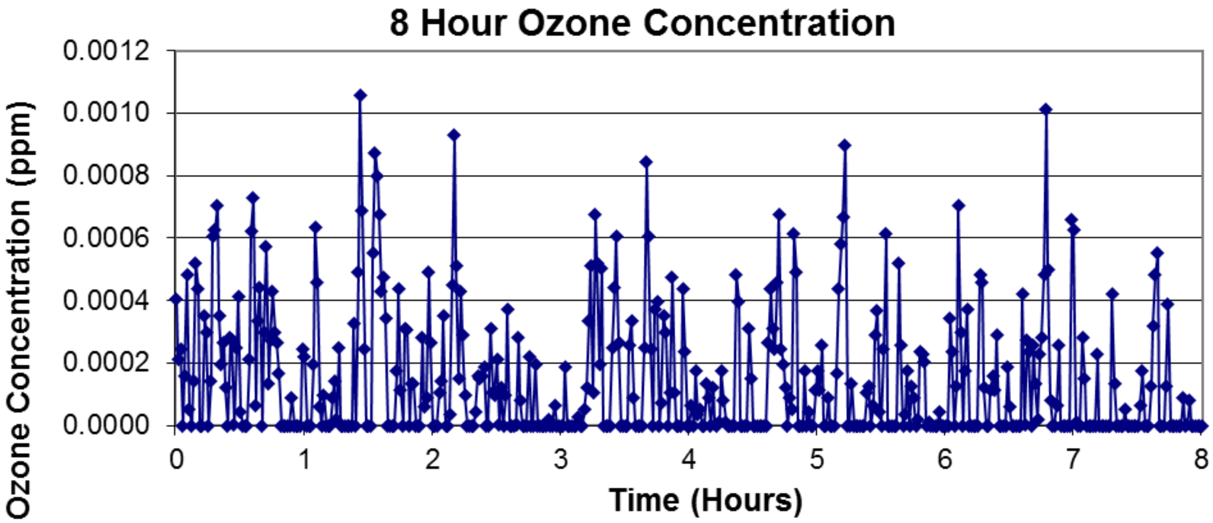

MAXIMUM OZONE TEST RESULTS							
	UL Ref.	Pass/Fail	Mean	Min	Max	Delta	Units
Background C(t) O3:	40.4.3	PASS	0.001	0.000	0.001	0.001	[ppm]
Test 1min C(t) O3:	40.1.2	PASS	0.000	0.000	**0.001**	0.001	[ppm]
Test 5min C(t) O3:	40.1.2	PASS	0.000	0.000	0.001	0.001	[ppm]
Chamber Temperature:	40.4.2	PASS	77	77	77	0	[degF]
Chamber Humidity:	40.4.2	PASS	50	49	52	3	[%RH]
Chamber Static Pressure:	-	PASS	0.02	-0.02	0.07	0.09	["H2O]
Chamber Supply Air Flow:	-	-	20	19	20	1	[SCFM]
Required to Test 2nd Sample:	40.1.1	NO					
Test Duration:	*40.4.6	8 hours					

NOTES: Peak Test Location 6

SECTION 6
APPENDIX

DATA FILES

TEST NAME	RAW DATA FILE
Model Half Life	3816 Halflife ozonelog.csv
Max Ozone: High Speed w/ Filter	3817 Max HIWIF ozonelog.csv
Max Ozone: Low Speed w/ Filter	3818 Max LOWIF ozonelog.csv
Max Ozone: Low Speed w/o Filter	3819 Max LOWOF ozonelog.csv

ATTACHMENT DOCUMENTS

DOCUMENT	SOFT-COPY FILE NAME
ARB Application	NA
Chain of Custody: Sample 1	COC_CRT1902201607-001,002.pdf
Chain of Custody: Sample 2	COC_CRT1902201607-001,002.pdf

UUT PHOTOGRAPHS

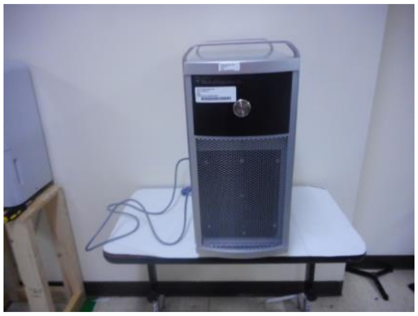

UUT

UUT PHOTOGRAPHS: PEAK TEST

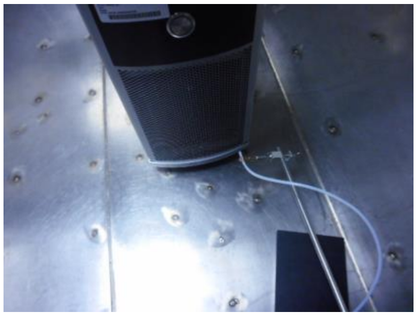

Location 6

HIGH SPEED w/ FILTER

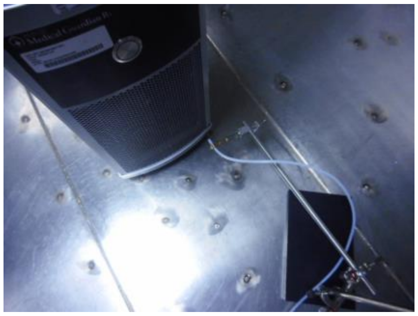

Location 6

LOW SPEED w/ FILTER

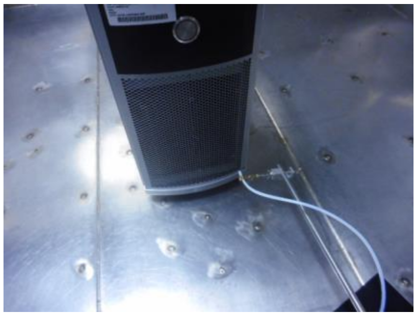

Location 6

LOW SPEED w/o FILTER

UUT PHOTOGRAPHS: MAX OZONE TESTS

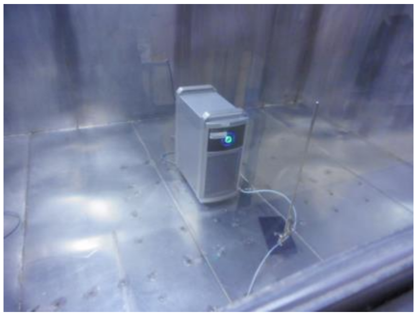

Location 6

HIGH SPEED w/ FILTER

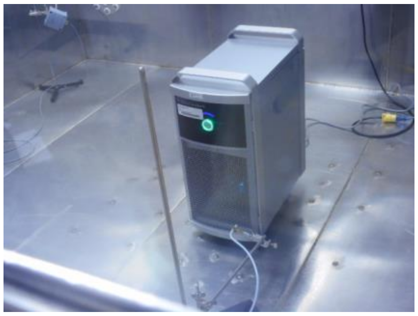

Location 6

LOW SPEED w/ FILTER

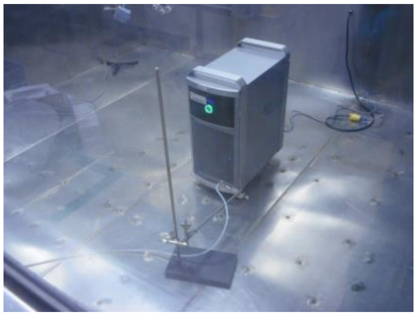

Location 6

LOW SPEED w/o FILTER
