# Supplementary material for: Evaluate the safety of a novel photohydrolysis technology used to clean and disinfect indoor air: A murine study
Source: PLoS One. 2024 Oct 9;19(10):e0307031. doi: 10.1371/journal.pone.0307031 (PMC11463749; doi:10.1371/journal.pone.0307031)
Supplement: S12 File — (PDF) [file pone.0307031.s012.pdf]

14841 Dallas Parkway
Suite 500, The Aberdeen Bldg.
Dallas, Texas 75254 USA

Ozone and Organic Oxidization By-products

Ozone and organic oxidation by-products are sometimes formed because of the irradiation of ambient moisture and oxygen in the air by UVC radiation with wavelengths in the range of 100~285nm. The Aerus Medical Guardian, model F170A was tested to determine if any organic oxidation by-products would be created by the device in operation.

The device was evaluated in a 3,000 cubic foot non-ventilated, environmentally controlled test area. Temperature was 73°F with a humidity level of 87%. The humidity level was increase beyond what would be normally found in a professional healthcare environment to ensure the ambient air was sufficiently saturated to promote oxidation by-products. Air samples were collected hourly over an 8-hour period, then 1 air sample was collected at 24 hours and another sample at 48 hours to determine if by-products were present and if so, at what level. The device was operated on the lowest fan speed.

Collected air samples were evaluated for the presents of the following chemicals;

Carbon monoxide
Formaldehyde
Acetaldehyde
Benzaldehyde
Toluene
TVOC*

"*" TVOC is a grouping of a wide range of organic chemical compounds to simplify reporting when these are present in ambient air or emissions.

Results of oxidation by-products testing performed is documented in the table on the following page.

14841 Dallas Parkway
Suite 500, The Aberdeen Bldg.
Dallas, Texas 75254 USA

**Test Date:**	11-May-19
**Device Tested:**	Aerus Medical Guardian, Model F170A
**Test Duration:**	48 hours
**Fan Speed**	Lowest Speed
**Environmental Test Room Size:**	3,000 ft3
**Environmental Test Room Temperature:**	73°F
**Environmental Test Room Humidity:**	87%
**Environmental Test Room Air:**	Non-ventilated
**Test Equipment:**	RAE Systems ppbRAE 3000

**VOC**	Carbon monoxide	Formaldehyde	Acetaldehyde	Benzaldehyde	Toluene	TVOC
Scale	**ppm**	**ppm**	**ppm**	**ppm**	**ppm**	**mg/m3**
Baseline Level	0.000	0.032	0.000	0.000	0.010	0.21
Hour 1	0.000	0.024	0.000	0.000	0.008	0.14
Hour 2	0.000	0.032	0.000	0.000	0.005	0.16
Hour 3	0.000	0.032	0.000	0.000	0.010	0.16
Hour 4	0.000	0.032	0.000	0.000	0.010	0.19
Hour 5	0.000	0.032	0.000	0.000	0.010	0.16
Hour 6	0.000	0.024	0.000	0.000	0.010	0.15
Hour 7	0.000	0.032	0.000	0.000	0.007	0.17
Hour 8	0.000	0.032	0.000	0.000	0.007	0.17
Hour 24	0.000	0.032	0.000	0.000	0.010	0.16
Hour 48	0.000	0.032	0.000	0.000	0.010	0.16
Peak Level	0.000	0.032	0.000	0.000	0.010	0.19
Average Level	0.00	0.030	0.00	0.00	0.01	0.16

Analysis of air samples has shown the Aerus Medical Guardian, model F170A in operation under the stated conditions does not create carbon monoxide, formaldehyde, acetaldehyde, benzaldehyde, toluene or TVOC's when compared to baseline levels.
